# Exploration of nonlinear optical enhancement in acceptor–π–donor indacenodithiophene based derivatives *via* structural variations: a DFT approach†

**DOI:** 10.1039/d3ra04858f

**Published:** 2023-09-22

**Authors:** Saba Abid, Muhammad Khalid, Muhammad Sagir, Muhammad Imran, Ataualpa A. C. Braga, Suvash Chandra Ojha

**Affiliations:** a Institute of Chemistry, Khwaja Fareed University of Engineering & Information Technology Rahim Yar Khan 64200 Pakistan muhammad.khalid@kfueit.edu.pk Khalid@iq.usp.br; b Centre for Theoretical and Computational Research, Khwaja Fareed University of Engineering & Information Technology Rahim Yar Khan 64200 Pakistan; c Institute of Chemical and Environmental Engineering, Khwaja Fareed University of Engineering & Information Technology Rahim Yar Khan 64200 Pakistan; d Department of Chemistry, Faculty of Science, King Khalid University P.O. Box 9004 Abha 61413 Saudi Arabia; e Departamento de Química Fundamental, Instituto de Química, Universidade de São Paulo Av. Prof. Lineu Prestes, 748 São Paulo 05508-000 Brazil; f Department of Infectious Diseases, The Affiliated Hospital of Southwest Medical University Luzhou 646000 China suvash_ojha@swmu.edu.cn

## Abstract

Herein, a series of indacenodithiophene-based derivatives (TNPD1–TNPD6) were designed having D–π–A architecture *via* end capped acceptor modulation of a reference molecule (TNPR) to investigate nonlinear optical (NLO) behavior. Quantum chemical calculations were accomplished to examine electronic, structural and optical properties utilizing a density functional theory (DFT) approach at M06 functional with 6-311G(d,p) basis set. For this, natural bond orbitals (NBOs), density of states (DOS), frontier molecular orbitals (FMOs), transition density matrix (TDM) and non-linear optical (NLO) analyses were performed for TNPR and TNPD1–TNPD6. The structural modifications revealed a significant electronic contribution in tuning the HOMOs and LUMOs of the derivatives with lowered energy gaps and wider absorption spectra. FMOs findings revealed that compound TNPD5 was found with the lowest energy gap (1.692 eV) and with the highest softness (0.591 eV^−1^) among the derivatives. Furthermore, a UV-Vis study also disclosed that maximum absorption (*λ*_max_ = 852.242 nm) was exhibited by TNPD5 in chloroform solvent. All the derivatives exhibited significant NLO results; in particular, TNPD5 showed the highest first hyper-polarizability (*β*_tot_ = 4.653 × 10^−27^ esu) and second hyper-polarizability (*γ*_tot_ = 9.472 × 10^−32^ esu). These DFT findings revealed that the end-capped substituents play a key role in enhancing the NLO response of these push–pull chromophores and the studied derivatives can be utilized as efficient NLO materials.

## Abstract 2

Herein, a series of indacenodithiophene-based derivatives (TNPD1–TNPD6) were designed having D–π–A architecture *via* end capped acceptor modulation of a reference molecule (TNPR) to investigate nonlinear optical (NLO) behavior.

## Graphical abstract

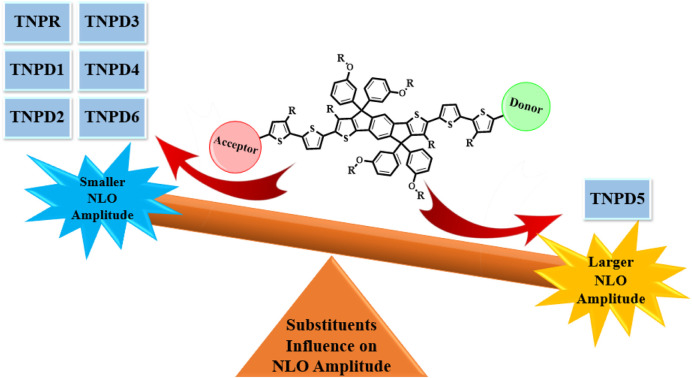

## Introduction

Nonlinear optical (NLO) materials play a significant role in modern technologies, as they possess the ability to change the frequency and phase of interacting light. There is a rapidly increasing interest in creating high-performance nonlinear optical (NLO) materials due to their many uses in optical computing,^1^ optical communication, fiber optics, data transformation, dynamic image processing and photonics.^2^ Some inorganic crystalline materials were the first to exhibit nonlinear optical (NLO) phenomena because their refractive indexes altered in response to an applied electric field.^3^ Following the discovery of the second harmonic generation (SHG) in an organic crystal in 1965, a number of promising researches have been conducted as chemists of that era have started to synthesize novel organic compounds with unique NLO properties.^4^ During the previous two decades, organic materials have been proposed as viable possibilities for future NLO uses *i.e.*, in a variety of photonic devices, due to their large nonlinearity, unique electronic and optical spectra, rapid response time, limited dispersion in refractive index and high structural versatility as compared to inorganic compounds. The NLO compounds may have high microscopic molecular nonlinearity (*β*), robust thermal stability, optimum photo stability, minimal optical absorption and low electrical interactions between molecules in a specific host matrix in order to produce acceptable device functioning.^5^ Organic nonlinear optical materials are preferably used because of their extended π-electron systems. The donor–π–acceptor configuration with excellent polarization in organic molecules can be used to infer the intra-molecular charge transfer process, which exhibits significant NLO features.^6^ Generally, the conjugated π-linker connecting the electron-donor (D) and acceptor (A) moieties in a molecule, is mainly responsible for the charge transfer (CT).^7^ The first hyper-polarizability (*β*) emerges from the NLO analysis and coincides with CT, which occurs from the donor to the acceptor with the aid of π-spacers. Earlier researchers have discovered a variety of D–π–A organic molecules tailored with various acceptors to establish a strong push–pull mechanism.^8^ These push–pull configurations influence charge dispersion, increase the range of penetration at greater wavelengths, amplify the distribution of electrons asymmetrically and reduce the energy band gap (*E*_LUMO_ − *E*_HOMO_), all of which increase the NLO response.^9^ In recent years, non-fullerene (NF) based compounds have attained significant growth in the field of photonic materials due to their distinctive optical and electrical properties, tunable energy levels, broad absorption spectra and reduced energy gap (*E*_gap_).^10^ Additionally, NFs with planar structures and configurable energy states exhibit significantly greater stability than organic compounds.^11^ By using the powerful donor and acceptor components in conjugated molecules, the NLO response of NF molecules can be enhanced by establishing a suitable π-conjugated systems.^12^ We selected *N*,*N*-diphenylnaphthalen-1-amine as a donor moiety due to its unique electrochemical and photo physical properties, high thermal stability and flexibility to structural modification makes it a suitable charge transporting material.^13^ Herein, we designed six A–π–D configured compounds (TNPD1–TNPD6) with indacenodithiophene core for NLO applications. DFT approach is accomplished to understand the electronic properties of these tailored compounds. It is anticipated that these fabricated molecules may be considered as significant NLO materials.

## Computational methodology

Quantum chemical calculations were performed at M06/6-311G(d,p)^14^ level of DFT^15^ to investigate the NLO properties, while time dependent density functional theory (TD-DFT)^16^ was used to investigate the electronic properties (DOS, FMO, TDM and GRPs) and absorption spectra (UV-Vis) of TNPR and TNPD1–TNPD6. Gaussian 09 program^17^ was utilized to accomplish the current study, and output files were developed by using Gauss View 6.0 (ref. 18) software. By using the conductor-like polarizable continuum model (CPCM),^19^ the effect of solvent (chloroform) on absorption properties was studied. For the selection of suitable functional for computational study, TNPR was optimized at M06,^20^ B3LYP,^21^ CAM-B3LYP,^22^ MPW1PW91,^21^ and *ω*B97XD,^23^ levels followed by the absorption spectral analysis in chloroform, and obtained *λ*_max_ findings were found as: 706.78, 798.40, 484.44, 782.92 and 454.63 nm, respectively (Tables S1–S5†). Among these, M06 functional showed *λ*_max_ (706.78 nm) in close concordance with experimental *λ*_max_ (682 nm) as described in Fig. 2. The NBOs, FMOs, GRPs, UV-Vis investigation, nonlinear optical (NLO) properties, TDM and DOS analyses were also accomplished at M06/6-311G(d,p) level of theory by using the optimized geometries of studied molecules. HOMO–LUMO energy band gaps were further utilized to examine the global reactivity parameters (GRPs) like kinetic stability, energy and reactivity of designed compounds. The interpretation of the output files was carried out using various software tools, including Gauss View 6.0,^18^ Avogadro,^24^ PyMOlyze 1.1,^25^ Multiwfn 3.8,^26^ GaussSum,^27^ Argus Labs,^28^ and Chemcraft 1.8.^29^

## Results and discussion

**Fig. 1:**
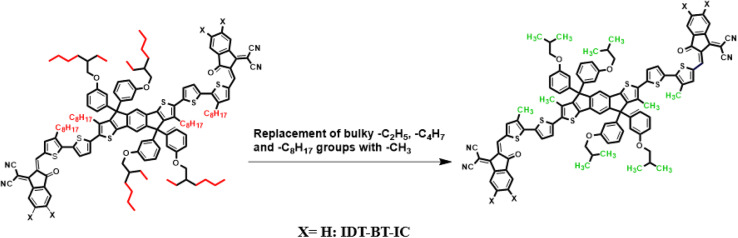
Modification of parent molecule (IDT-BT-IC) into TNPR by replacing bulky alkyl groups with –CH_3_ group.

**Scheme 1:**
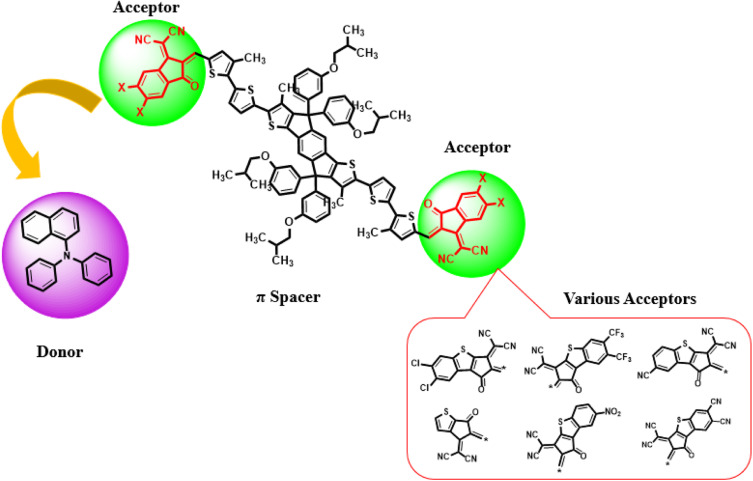
Schematic depiction of entitled chromophores TNPR and TNPD1–TNPD6.

Current study focused on the computational investigation of NLO properties of a series of indacenodithiophene based chromophores (TNPR and TNPD1–TNPD6). For this study synthesized parent molecule (IDT-BT-IC)^30^ having A–D–A configuration was modified *via* structural tailoring to develop reference (TNPR) compound as shown in Fig. 1. In order to attain a simple push–pull A–π–D configuration, one of the terminal acceptor of TNPR is replaced with a donor group *i.e.*, *N*,*N*-diphenylnaphthalen-1-amine, while other terminal acceptor is replaced by 2-(3-oxo-2,3,3*a*,8*b*-tetrahydro-1*H*-benzo[*b*]cyclopenta[*d*]thiophen-1-ylidene)malononitrile possessing one cyano group in TNPD1. In derivatives, TNPD2–TNPD5, two chloro, two fluoro, one nitro and two cyano groups are attached, with benzene ring of the acceptor group, respectively. In TNPD6, the utilized acceptor moiety is 2-(2-methylene-3-oxo-2,3-dihydro-1*H*-inden-1-ylidene)malononitrile (Scheme 1). The structural and optimized views of the title compounds are displayed in Fig. S3 and S4,† respectively. Cartesian co-ordinates and IUPAC names of the newly designed compounds are presented in Tables S6–S12† and S40,† respectively. To study the effect of structural tailoring on NLO properties, HOMO–LUMO band gaps, optical properties, average linear polarizability 〈*α*〉, first hyper-polarizability (*β*_tot_) and second hyper-polarizability (*γ*_tot_) values are computed. This research paper will prove a remarkable addition in the field of nonlinear optics and might surely urge the experimental researcher to synthesize these chromophores with improved NLO properties.

**Fig. 2:**
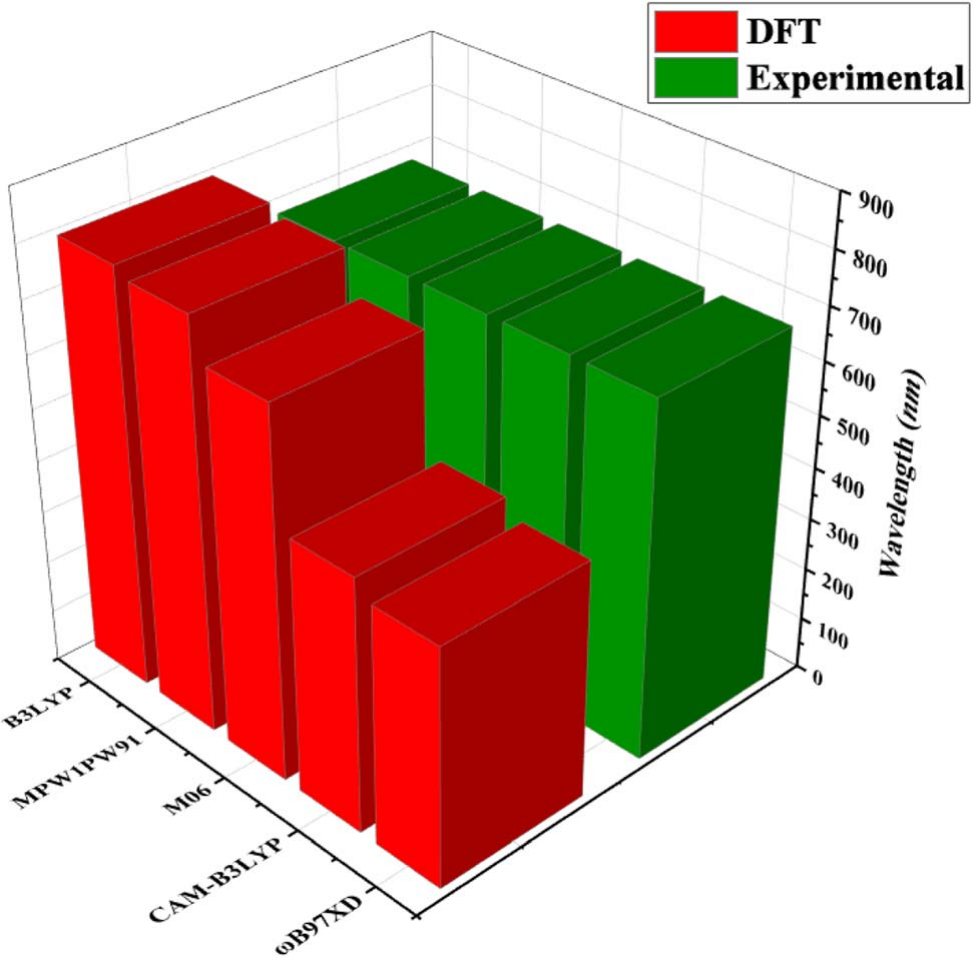
Comparison of absorption maxima (*λ*_max_) values of TNPR between experimental and theoretical results simulated at five different functionals.

### Natural bond orbitals (NBOs) analysis

NBOs analysis is an invaluable method for investigating charge delocalization, molecular stability, electronic structure and hybridization. It provides valuable insights into the interactions between occupied and unoccupied orbitals, as well as intra-molecular and intermolecular hydrogen bonding.^31,32^ NBOs analysis of TNPR and its derivative (TNPD1–TNPD6) is done by using the NBO 6.0 (ref. 33) program at the same functional and the resulting data, including characteristic transitions, have been recorded in Tables S13–S19.† However, some selected transitions with their respective energy values are presented in Table 1. To calculate the stabilization energies in the NBOs, we employed a second order perturbation process and utilized eqn (1).^34,35^1
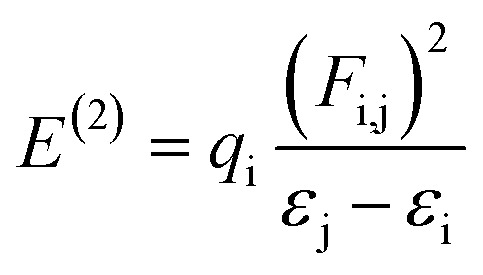
In eqn (1), (*E*^(2)^) defines the energy of stabilization in NBOs, where “i” and “j” represent donor and acceptor orbitals, correspondingly. The occupancy of the orbitals is denoted by “*q*_i_”. The off-diagonal NBO Fock matrix is represented as “*F*_i,j_” and the diagonal NBO Fock matrix is indicated by “*ε*_j_” and “*ε*_i_”.

**Table 1:** NBOs results of investigated chromophores for TNPR and TNPD1–TNPD6

Compounds	Donor (i)	Type	Acceptor (j)	Type	*E* ^(2)^ [kcal mol^−1^]	*E*(j) − *E*(i) [a.u]	*F*(i,j) [a.u]
TNPR	C38–C39	π	C112–C113	π*	31.17	0.31	0.088
	C16–C17	π	C63–C64	π*	0.79	0.33	0.015
	C109–H111	σ	S43–C45	σ*	10.29	0.73	0.077
	C59–H60	σ	C10–C11	σ*	0.50	1.07	0.021
	O154	LP(2)	C78–C80	π*	33.27	0.36	0.104
	O141	LP(2)	C115–C136	σ*	20.82	0.76	0.114
TNPD1	C44–C45	π	C109–C110	π*	30.87	0.31	0.088
	C120–N121	π	C122–N123	π*	0.63	0.47	0.015
	C109–H111	σ	S43–C45	σ*	10.01	0.73	0.076
	C120–N121	σ	C116–C118	σ*	0.50	1.65	0.026
	O127	LP(2)	C78–C80	π*	33.18	0.36	0.104
	O119	LP(2)	C112–C117	σ*	21.58	0.75	0.116
TNPD2	C44–C45	π	C109–C110	π*	30.53	0.31	0.087
	C120–N121	π	C122–N123	π*	0.63	0.47	0.015
	C109–H111	σ	S43–C45	σ*	9.97	0.73	0.076
	C120–N121	σ	C116–C118	σ*	0.50	1.65	0.026
	O127	LP(2)	C78–C80	π*	33.17	0.36	0.078
	O119	LP(2)	C112–C117	σ*	21.51	0.76	0.115
TNPD3	C44–C45	π	C109–C110	π*	31.36	0.31	0.088
	C120–N121	π	C122–N123	π*	0.63	0.47	0.015
	C109–H111	σ	S43–C45	σ*	10.06	0.73	0.077
	C120–N121	σ	C116–C118	σ*	0.50	1.65	0.026
	O127	LP(2)	C78–C80	π*	33.2	0.36	0.104
	O119	LP (2)	C112–C117	σ*	21.74	0.75	0.116
TNPD4	C44–C45	π	C109–C110	π*	31.01	0.31	0.088
	C20–N121	π	C122–N123	π*	0.63	0.47	0.015
	C109–H111	σ	S43–C45	σ*	10.01	0.73	0.076
	C120–N121	σ	C116–C118	σ*	0.50	1.65	0.026
	O127	LP (2)	C78–C80	π*	33.17	0.36	0.104
	O119	LP(2)	C112–C117	σ*	21.69	0.75	0.116
TNPD5	C44–C45	π	C109–C110	π*	32.04	0.31	0.089
	C122–N123	π	C120–N121	π*	0.63	0.47	0.015
	C109–H111	σ	S43–C45	σ*	10.08	0.73	0.077
	C120–N121	σ	C116–C118	σ*	0.50	1.64	0.026
	O127	LP (2)	C78–C80	π*	33.18	0.36	0.104
	O119	LP(2)	C112–C117	σ*	21.74	0.75	0.116
TNPD6	C44–C45	π	C109–C110	π*	29.78	0.31	0.087
	C122–N123	π	C120–N121	π*	0.62	0.47	0.015
	C109–H111	σ	S43–C45	σ*	9.94	0.73	0.076
	C120–N121	σ	C116–C118	σ*	0.50	1.64	0.026
	O127	LP(2)	C78–C80	π*	33.19	0.36	0.104
	O119	LP(2)	C112–C117	σ*	21.56	0.76	0.116

The NBOs analysis reveals the presence of four distinct types of transitions observed in TNPR and its derivatives (TNPD1–TNPD6). These transitions include π to π*, σ to σ*, lone pair (LP) to σ*, and LP to π* transitions. The π → π* transitions, specifically π(C38–C39) → π*(C112–C113) and π(C16–C17) → π*(C63–C64) play a crucial role in stabilization of TNPR. These transitions provide the highest and lowest stabilization values of 31.17 and 0.79 kcal mol^−1^, respectively. Similarly, the σ to σ* transitions; σ(C109–H111) → σ*(S43–C45) with the maximum energy value of 10.29 kcal mol^−1^ and σ(C59–H60) → σ*(C10–C11) with the minimum energy value of 0.50 kcal mol^−1^ are observed in TNPR. The other transitions *i.e.*, LP2(O154) → π*(C78–C80) and LP2(O141) → σ*(C115–C136) have the maximum energy values of 33.27 and 20.82 kcal mol^−1^, correspondingly, for TNPR.

For TNPD1, TNPD2 and TNPD3, π → π* same transitions such as π(C44–C45) → π*(C109–C110) with highest stabilization energies *i*.*e*., 30.87, 30.53 and 31.36 kcal mol^−1^ and same electronic transitions with lowest stabilization energies such as π(C120–N121) → π*(C122–N123) with a value of 0.63 kcal mol^−1^, respectively. The σ → σ*, same transitions; σ(C109–H111) → σ*(S43–C45) with highest stabilization energies such as 10.01, 9.97 and 10.06 kcal mol^−1^ and σ(C1120–N121) → σ*(C116–C118) with smallest stabilization energy 0.50 kcal mol^−1^ are observed in TNPD1, TNPD2 and TNPD3, respectively. LP → π* transition such as: LP2(O127) → π*(C78–C80) have highest energy values as 33.18, 33.17 and 33.2 kcal mol^−1^ for TNPD1, TNPD2 and TNPD3, respectively. While LP → σ* same transitions, LP2(O119) → σ*(C112–C117) have uppermost energies as 21.58, 21.51 and 21.74 kcal mol^−1^ for TNPD1, TNPD2 and TNPD3, correspondingly.

In TNPD4, TNPD5 and TNPD6, π → π* same electronic transitions such as π(C44–C45) → π*(C109–C110) with highest stabilization energies as 31.01, 32.04 and 29.78 kcal mol^−1^ and π(C120–N121) → π*(C122–N123), π(C122–N123) → π*(C120–N121) and π(C122–N123) → π*(C120–N121) electronic transitions with lowest stabilization energies as 0.63, 0.63 and 0.59 kcal mol^−1^ are noticed, respectively. The σ → σ*, same transitions as σ(C109–H111) → σ*(S43–C45) with highest stabilization energies as 10.01, 10.08 and 9.94 kcal mol^−1^ and σ(C120–N121) → σ*(C116–C118) with lowest stabilization energy 0.50 kcal mol^−1^ for TNPD4, TNPD5 and TNPD6, respectively, are recorded. LP → π* transition: LP2(O127) → π*(C78–C80) have highest values as 33.17, 33.18 and 33.19 kcal mol^−1^ are found for TNPD4, TNPD5 and TNPD6, correspondingly. While, LP → σ* same transitions, LP2(O119) → σ*(C112–C117) have highest energies as 21.69, 21.74 and 21.56 kcal mol^−1^ are observed in TNPD4, TNPD5 and TNPD6, respectively.

The above findings demonstrated that TNPD5 exhibited exceptional stability (32.04 kcal mol^−1^) as compared to the other derivatives, which can be attributed to its prolonged hyper-conjugation. Shortly, the NBOs analysis of all the studied compounds revealed that the transfer of charge and extended hyper-conjugation play key role in stabilization of TNPD1–TNPD6, ultimately leading to their distinct nonlinear optical properties.

### Electronic properties

The exploration of electronic properties in the studied compounds is effectively accomplished through the investigation of frontier molecular orbitals (FMOs), which consist of two main orbitals: LUMO responsible for electron acceptance and HOMO exhibiting electron donation ability.^36^ The determination of the energy gap (*E*_gap_) between HOMO and LUMO through FMOs analysis, plays a crucial role in assessing various global reactivity characteristics *i.e.*, dynamic stability, chemical hardness, softness and reactivity, providing valuable insights into the investigated systems^37^ and optical properties of the studied systems.^38^Table 2 presents the calculated results for *E*_HOMO_, *E*_LUMO_ and *E*_LUMO_ − *E*_HOMO_ (*E*_gap_) for TNPR and TNPD1–TNPD6 compounds.

**Table 2:** *E*_HOMO_, *E*_LUMO_ and energy gap (*E*_gap_ = *E*_LUMO_ − *E*_HOMO_) of entitled molecules^a^

Systems	*E*_(HOMO)_	*E*_(LUMO)_	*E*_gap_
TNPR	−5.499	−3.303	2.196
TNPD1	−5.294	−3.428	1.866
TNPD2	−5.291	−3.411	1.880
TNPD3	−5.308	−3.501	1.807
TNPD4	−5.297	−3.445	1.825
TNPD5	−5.303	−3.611	1.692
TNPD6	−5.297	−3.218	2.079

a Units in eV.

**Fig. 3:**
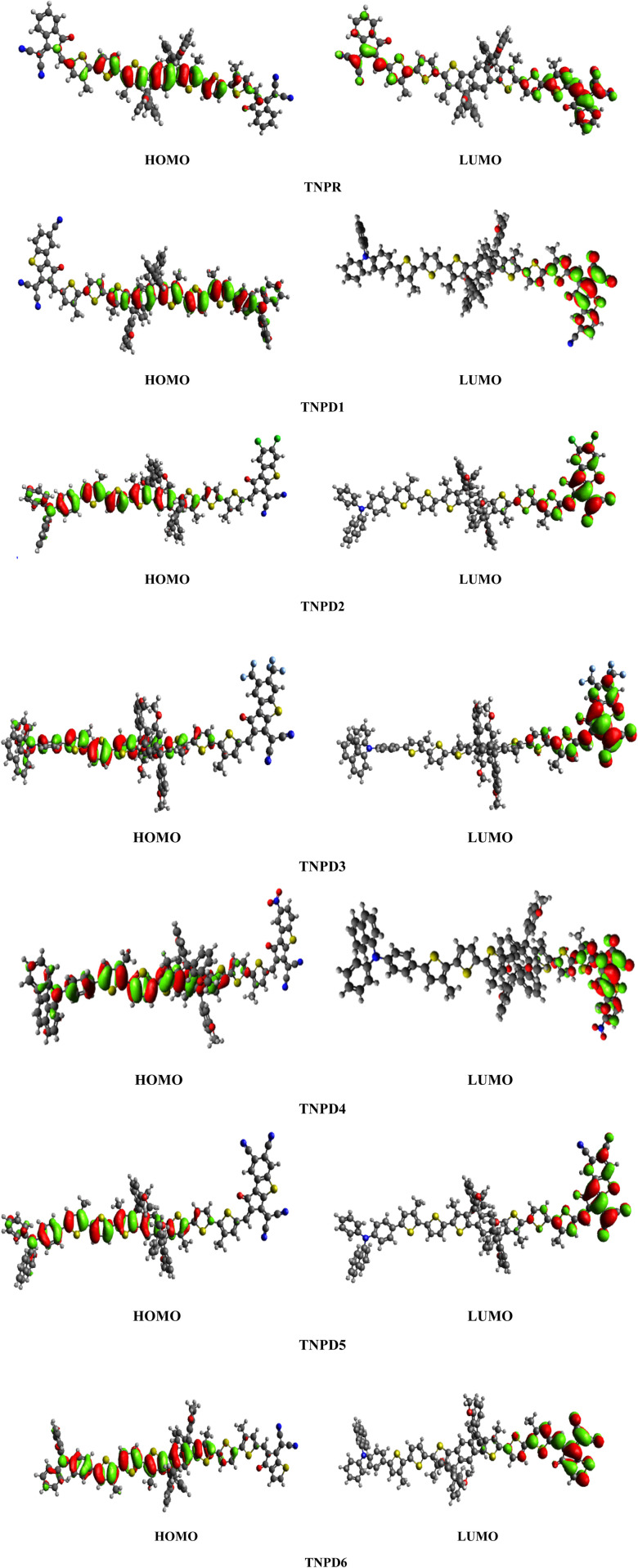
The contour surface diagram of HOMOs and LUMOs of entitled compounds.

Table 2 shows that the HOMO/LUMO energies (−5.499/−3.303 eV) and band gap (*E*_gap_ = 2.196 eV) of the reference chromophore (TNPR) is in good agreement with the experimental values (−5.25/−3.82 eV and *E*_gap_ = 1.43 eV).^39^ In TNPD1–TNPD6, a decrease in the *E*_gap_ is observed, possibly attributed to the electron capturing nature of substituents (chloro, fluoro and cyano) located at acceptor group in comparison to the TNPR. However, TNPD5 demonstrates the smallest *E*_gap_ value of 1.692 eV, owing to the presence of two cyano groups at the terminal acceptor. The electron-withdrawing nature of the cyano groups substituted at the end-capped acceptor moiety contributes to the reduction in energy band gap.^40^ Decreasing order of *E*_gap_ for investigated compounds is as follows: TNPR (2.196) > TNPD6 (2.079) > TNPD2 (1.880) > TNPD1 (1.866) > TNPD4 (1.825) > TNPD3 (1.807) > TNPD5 (1.692) in eV. The energy band gap plays a crucial role in facilitating the charge transfer process, as a smaller band gap leads to higher charge transfer. Fig. 3 illustrates contour surface diagrams of the HOMOs and LUMOs, demonstrated that the HOMOs primarily located over the π-spacer with minimal influence on the donor part. Conversely, the LUMOs exhibit a significant charge density on the acceptor part, with a lesser contribution from the π-spacer, among all the studied compounds. The presence of a π-linker facilitates efficient CT amongst electron donating and electron accepting parts of the molecule, highlighting the potential of these molecular systems as NLO materials. Table S20† provides the values for HOMO−1, LUMO+1, HOMO−2, and LUMO+2, and Fig. S2† presents the corresponding counter surface diagrams.

### Global reactivity parameters (GRPs)

The reactivity and stability of TNPR and TNPD1–TNPD6 were investigated by computing the GRPs using the energy gap (*E*_gap_) among HOMOs and LUMOs.^41^ The *E*_gap_ serves as a dynamic indicator for determining various GRPs, including hardness (*η*), ionization potential (IP), chemical potential (*μ*), electronegativity (*X*), electrophilicity index (*ω*), softness (*σ*) and electron affinity (EA). The GRPs of entitled compounds are computed at M06 level and outcomes are displayed in the Table 3.^42–46,53^

**Table 3:** Global reactivity descriptors for TNPR and TNPD1–TNPD6^a^

Compounds	IP	EA	*X*	*η*	*μ*	*ω*	*σ*	Δ*N*_max_
TNPR	5.499	3.303	4.401	1.098	−4.401	8.820	0.455	4.008
TNPD1	5.294	3.428	4.361	0.933	−4.361	10.192	0.535	4.674
TNPD2	5.291	3.411	4.351	0.940	−4.351	10.069	0.531	4.628
TNPD3	5.308	3.501	4.404	0.903	−4.404	10.735	0.553	4.877
TNPD4	5.297	3.445	4.371	0.926	−4.371	10.316	0.539	4.720
TNPD5	5.303	3.611	4.457	0.846	−4.457	11.740	0.591	5.268
TNPD6	5.297	3.218	4.257	1.039	−4.257	8.718	0.481	4.097

a Unit = eV.

Electron affinity (EA = −*E*_LUMO_) and ionization potential (IP = −*E*_HOMO_) are calculated by using provided formulas. The electronegativity [*X* = (IP + EA)/2], chemical potential [*μ* = (*E*_HOMO_ + *E*_LUMO_)/2] and chemical hardness [*η* = (IP − EA)] are determined based on Koopman's theorem.^47^ Parr *et al.* introduced an electrophilicity index proposed by the equation (*ω* = *μ*^2^/2*η*). The global softness (*σ* = 1/*η*) is computed. Δ*N*_max_ = −*μ*/*η*,^48^ is the ability of a compound to absorb the more electrical charge from its surrounding. The electron affinity (EA) and ionization potential (IP) represent the tendencies for electron accepting and electron donation, respectively, and correspond to energy required to add or remove an electron from valence band (HOMOs). The chemical stability and reactivity of the chromophores are influenced by their chemical potential (*μ*). The interrelationships between hardness, energy gap, stability and chemical potential of a molecule are direct, while they exhibit an inverse relationship with reactivity.^49^ The molecular stability is influenced by electronegativity of substituents and their position relative to electronegative fragments. Electronegativity plays a role in determining the molecule's ability to accept the incoming electrons. A molecule with greater (*E*_gap_) possesses higher hardness, increased stability and decreased reactivity.^50^ Additionally, IP and EA are supplementary parameters that provide insights into the reactivity of molecules due to their direct correlation with polarizability. The TNPR and TNPD1–TNPD6 exhibit elevated ionization potential and reduced electron affinity, as indicated in Table 3. Among all the derivatives, IP of TNPR is found to be maximum at 5.499 eV. The decreasing order of IP is as: TNPD3 > TNPD5 > TNPD6 = TNPD4 > TNPD1 > TNPD2 with values as: 5.308 > 5.303 > 5.297 = 5.297 > 5.294 > 5.291 > eV, correspondingly. The relationship between softness (*σ*) and hardness (*η*) of a molecule is related to its *E*_gap_ and offers valuable information regarding the reactivity of the system. Hardness is directly linked to the band gap, while it exhibits an inverse correlation with reactivity. A larger band gap corresponds to higher hardness, resulting in reduced intra-molecular charge transfer and lower reactivity. On the other hand, a smaller energy gap (*E*_gap_) leads to higher softness and polarizability of the chromophores, indicating increased reactivity. Table 3 illustrates that among all the derivatives, TNPD6 reveals the highest hardness value (1.039 eV), while lowest softness value (0.481 eV^−1^). The decreasing order of hardness is as: TNPD6 > TNPD2 > TNPD1 > TNPD4 > TNPD3 > TNPD5. In addition, the chemical potential is utilized to evaluate stability and reactivity of chromophores, which is supported by the direct relation with stability and inverse with reactivity of molecules. Among all the derivatives, TNPD5 exhibits the maximum value (−4.457 eV) of chemical potential. Furthermore, the decreasing order of Δ*N*_max_ is; TNPD5 > TNPD3 > TNPD4 > TNPD1 > TNPD2 > TNPD6 > TNPR. The calculated GRPs data for listed molecules is related to the *E*_LUMO_ − *E*_HOMO_, where compounds with smaller *E*_gap_ exhibit lower chemical potential and hardness but greater softness. Remarkably, TNPD5 demonstrates the maximum softness value (0.591 eV^−1^) and is considered the highest polarizable species among all the investigated chromophores, displaying prospective, nonlinear optical (NLO) properties.

### UV-Vis analysis

**Fig. 4:**
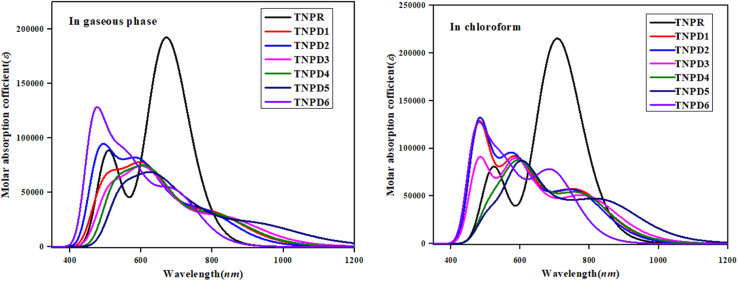
UV-Vis plots for TNPR and TNPD1–TNPD6 in gaseous phase and chloroform solvent.

UV-Vis investigation provides crucial electronic information regarding the types of electronic transitions, configurations influencing transitions and the ability of molecules to transmit charge.^51^ It also shows a relation between chemical structure of derivatives and their suitability as nonlinear optical (NLO) materials. Table 4 displays the molecular transitions of TNPR and TNPD1–TNPD6, at same level of TD-DFT along with transition energy (*E*), oscillator strength (*f*_os_), and maximum absorption wavelength (*λ*_max_). UV-Vis investigation is performed using both chloroform solvent and gaseous phase and the simulated absorption spectra of the studied compounds is shown in Fig. 4. The six lowest singlet–singlet transitions are displayed in Tables S21–S34.†

**Table 4:** Excitation energies (*E*), oscillator strength (*f*_os_), wavelength (*λ*) and contributions of various molecular orbitals of compounds TNPR, TNPD1–TNPD6 in gas and solvent phase

	Compounds	DFT *λ* (nm)	*E* (eV)	*f*_os_	MO contributions
A^a^	TNPR	671.128	1.847	2.637	H → L (86%), H−1 → L+1 (5%)
	TNPD1	807.241	1.536	0.387	H → L (93%), H−1 → L (5%)
	TNPD2	783.668	1.582	0.407	H → L (92%), H−1 → L (6%)
	TNPD3	843.602	1.470	0.339	H → L (94%), H−1 → L (5%)
	TNPD4	816.814	1.518	0.369	H → L (94%), H−1 → L (5%)
	TNPD5	931.302	1.331	0.264	H → L (96%), H−1 → L (3%)
	TNPD6	697.442	1.778	0.653	H → L (90%), H−1 → L (7%)
B^b^	TNPR	706.785	1.754	2.960	H → L (86%), H−1 → L+1 (8%)
	TNPD1	774.127	1.602	0.704	H → L (83%), H−2 → L (3%)
	TNPD2	766.754	1.617	0.690	H → L (82%), H−2 → L (3%)
	TNPD3	799.279	1.551	0.627	H → L (85%), H−2 → L (2%)
	TNPD4	780.315	1.589	0.665	H → L (84%), H−2 → L (3%)
	TNPD5	852.242	1.455	0.553	H → L (87%), H−1 → L (10%)
	TNPD6	694.745	1.785	0.997	H → L (79%), H−2 → L (3%)

a A = gas phase.

b B = solvent phase (chloroform).

Table 4 reveals that the computed *λ*_max_ for TNPR is 706.785 nm, which shows excellent agreement with experimental absorption peak observed at 682 nm.^39^ The *λ*_max_ values for TNPD1–TNPD6 range from 694.745 to 852.242 nm, with corresponding transition energies in the range of 1.455 to 1.785 eV and oscillation strengths ranging from 0.553 to 0.997 in chloroform solvent. These transitions arise from the H → L interactions governed by the A–π–D configuration in the studied chromophores. The presence of this type of CT is responsible for the NLO behavior exhibited by the studied chromophores. Increasing *λ*_max_ order observed in chloroform is as follows: TNPD5 (852.242 nm) > TNPD3 (799.279 nm) > TNPD4 (780.315 nm) > TNPD1 (774.127 nm) > TNPD2 (766.754 nm) > TNPD6 (694.745 nm). Amongst all the derivatives, TNPD5 exhibits the highest *λ*_max_ value of 852.242 nm, accompanied by a transition energy of 1.455 eV and an oscillator strength of 0.553. This is attributed to a dominant H → L (87%) electronic transition, complete CT and the polarity of solvent. On the other hand, TNPD6 demonstrates the lowest absorption properties among the designed chromophores due to its limited charge-withdrawing ability, resulting in hindered charge delocalization in acceptor region. In the gaseous phase, the *λ*_max_ range is found to be 697.442–931.302 nm. The decreasing order of the designed chromophores based on *λ*_max_ in the gaseous phase is: TNPD5 > TNPD3 > TNPD4 > TNPD1 > TNPD2 > TNPD6.

TNPD5 stands out among the derivatives by displaying a significant wavelength of 931.302 nm in the gaseous phase. The distinctive contribution of different orbitals in the electronic transitions is due to the structural modifications involving various acceptor groups in TNPR. The essential excited states in the studied compounds arise from electron shifts between the HOMO and LUMO. Interestingly, a consistent decrease in *λ*_max_ values is observed in both solvent and gaseous phases across all derivatives. This trend can be attributed to the distinct acceptor groups and the positioning of electron withdrawing substituents attached to the acceptor part. Overall, a blue shifted absorption spectra are observed for most molecules in the gaseous phase. Previous literature supports the idea that the absorption maxima strongly depend on polarity and nature of solvent, as evidenced by notable bathochromic shift (red-shifted) observed, with chloroform solvent showing the highest shift among all the A–π–D configured compounds.^52^

### Transition density matrix (TDM) analysis

The analysis of TDM provides a powerful approach to assess and interpret electronic excitation mechanisms in molecular systems. The study of the TDM in a many-body system offers a spatial heat map that depicts the scattering patterns of electron hole pairs during transitions between two eigen states. This analysis allows for the identification of the extent of delocalization and coherence lengths involved in these transitions.^53,54^

**Fig. 5:**
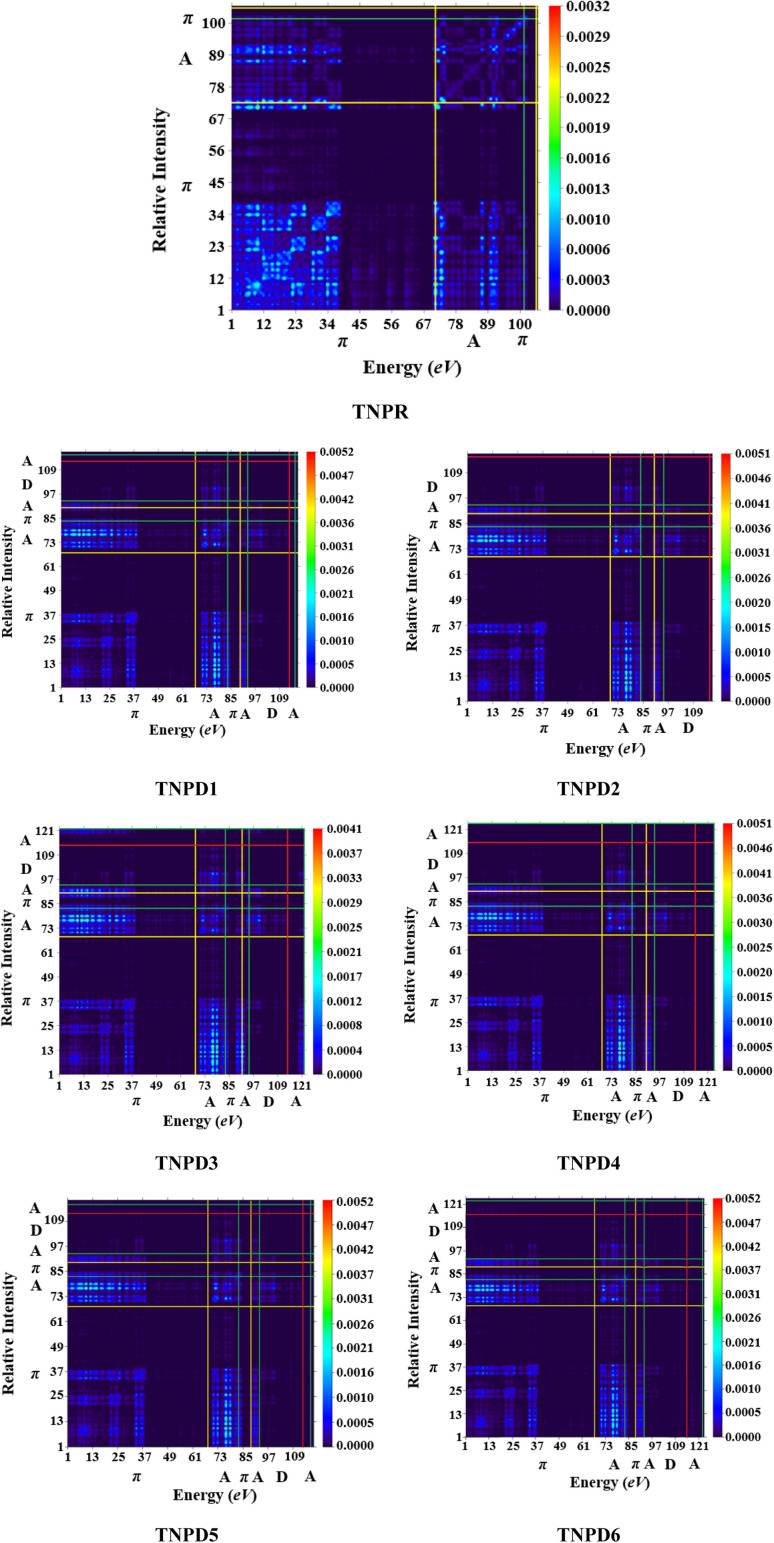
TDM pictographs of the entitled molecules (TNPR and TNPD1–TNPD6); in above diagrams, “A” stands for acceptor; “D” stands for donor “π” stands for π-spacer.

For the TDM analysis in this study, the reference compound (TNPR) is divided into two sections: the terminal acceptor (A) and the central π-spacer. Similarly, the derivatives (TNPD1–TNPD6) are divided into three sections: central π-spacer, donor (D) group and acceptor (A) group as shown in Fig. 5. In this study, the influence of hydrogen atoms on the transitions ware considered negligible; therefore, their impact were ignore. The transition density matrix (TDM) of TNPR and TNPD1–TNPD6 were obtained using the specified level of density functional theory (DFT), and their corresponding pictographs are presented in Fig. 5.

The FMOs reveals that charge density is predominantly concerted in π-spacer and acceptor (A) groups, resulting in significant variations in the TDM pictographs. Fig. 5 illustrates the efficient diagonal transmission of electron density from π-bridge to acceptor, with minimal influence from the donor segment in all the derivatives. This enables efficient charge transfer without encountering any trapping phenomena. The TDM pictographs demonstrate effortless and enhanced charge separation from the ground state (S0) to the excited state (S1). The TDM heat maps for TNPD1–TNPD6 suggest a well-defined, easier and improved exciton separation in excited state, indicating potential applications in the future.

### Exciton binding energy (*E*_b_) analysis

The binding energy (*E*_b_) is a crucial parameter for predicting the effectiveness of different materials, particularly in terms of their charge mobility and nonlinear optical (NLO) response^55^ The binding energies of reference molecule and designed molecules (TNPD1–TNPD6), were investigated using the M06/6-311G(d,p) level by employing the eqn (2).

2 *E*_b_ = *E*_L–H_ − *E*_opt_

In eqn (2), *E*_opt_ represents the first excitation energy from the ground state (S0) → first singlet excited state (S1).^56^ On the other hand, *E*_L–H_ denotes *E*_gap_ between the HOMO and LUMO, which is used to estimate the binding energy.^57^ Theoretical calculations for the binding energy are summarized in Table 5. These calculations provide valuable insights into the exciton binding energies of TNPR and TNPD1–TNPD6, shedding light on their potential efficacy as materials for various applications.

**Table 5:** The calculated values for the HOMO–LUMO *E*_gap_, the initial singlet excitation energy (*E*_opt_), and the exciton binding energy^a^

Compounds	TNPR	TNPD1	TNPD2	TNPD3	TNPD4	TNPD5	TNPD6
*E*_L–H_	2.196	1.866	1.880	1.807	1.825	1.692	2.079
*E*_opt_	1.754	1.602	1.617	1.551	1.589	1.455	1.785
*E*_b_	0.442	0.264	0.263	0.256	0.236	0.273	0.294

a Units in eV.

According to the results, the binding energy of a reference compound TNPR is 0.442 eV. Interestingly, all the derivatives (TNPD1–TNPD6) binding energy values as 0.264, 0.263, 0.256, 0.273 and 0.294 have lower *E*_b_ values when contrasted with the reference molecule (TNPR). The chromophore with the least value of binding energy, TNPD4, has 0.236 eV, indicating that it has a lot of charges and is simple to separate them into distinct charges. The decreasing order of binding energy for all examined compounds in a good agreement with the TDM analysis is: TNPR > TNPD6 > TNPD5 > TNPD1 > TNPD2 > TNPD3 > TNPD4.

### Density of states (DOS) analysis

DOS analysis is accomplished to further validate the results of FMOs analysis (Fig. 3). To facilitate DOS research, the compounds (TNPR and TNPD1–TNPD6) were divided into three distinct segments such as: donor, π-spacer and acceptor represented by green, blue and red colored lines, respectively, as presented in Fig. S1.† This graphical depiction of the DOS provides additional empirical evidence and enhances the understanding of the electronic structure of the studied compounds. In these graphs, negative values correspond to the HOMO, also known as the valence band, while positive values represent the LUMO, which acts as the conduction band. The energy gap, representing the separation between HOMO and LUMO, is depicted along the *x*-axis.^58^ By analyzing the percentages of density of states surrounding the LUMO and HOMO, the investigation of frontier molecular orbitals (FMOs) revealed that the charge transport pattern on molecular orbitals can be altered by the inclusion of various electron-deficient groups.^59^ The percentages of density of states associated with the LUMO and HOMO are presented in Table S35.† The donor's electronic contribution in HOMO for designed compounds (TNPD1–TNPD6) is shown here as 20.8, 21.3, 20.3, 19.5, 22.7 and 19.4%, whereas, the contribution to LUMO is 0.0, 0.0, 0.0, 0.0, 0.0, and 0.0% respectively. Similarly, for TNPR and TNPD1–TNPD6, the contributions of the π-spacer at HOMO are 92.4, 78.1, 77.7, 78.7, 79.4, 76.3 and 79.6%, while at LUMO they are 37.4, 18.0, 17.5, 17.1, 17.3, 16.0 and 24.6%, respectively (Table S35†). Acceptor showed participation for the examined compounds at HOMO as 7.6, 1.1, 1.0, 1.0, 1.1, 1.0 and 1.0% and LUMO as 62.6, 82.0, 82.5, 82.9, 82.7, 84.0 and 75.4%, respectively. The analysis of proposed compounds (TNPD1–TNPD6) reveals a noteworthy observation, the presence of charge delocalization and a substantial transfer of charge from the electron rich donor to the electron-withdrawing end-capped acceptor moiety through π-bridge. This phenomenon is consistent across all investigated compounds, establishing the framework for efficient charge transportation.

### Nonlinear optical (NLO) properties

The increasing demand for nonlinear optical (NLO) materials in nuclear sciences, signal manipulation and optoelectronic devices has significant interest in recent years.^60^ Pull–push configurations of compounds are developed to produce the NLO response; their robustness depends on the types of donor and acceptor moieties that are linked together through the π-linker.^61^ The electronic properties of these materials are closely related to their average linear polarizability (〈α〉), first hyper-polarizability (*β*_tot_) and second hyper-polarizability (*γ*_tot_) responses, which also contribute to their optical activity. Hence, to estimates the effect of donor and acceptor groups on the linear and nonlinear response of TNPR and TNPD1–TNPD6, their dipole moment, 〈α〉, *β*_tot_ and *γ*_tot_ were calculated and the results are displayed in Tables S36–S39† and major tensors are listed in Table 6. The dipole moment (*μ*_total_),^62^ average linear polarizability 〈*α*〉^63^ and first hyper-polarizability (*β*_tot_)^64^ values were calculated by the using eqn (3)–(5).

3 *μ* = (*μ*_*x*_^2^ + *μ*_*y*_^2^ + *μ*_*z*_^2^)^1/2^

4 〈*α*〉 = 1/3(*α*_*xx*_ + *α*_*yy*_ + *α*_*zz*_)

5 *β*_tot_ = (*β*_*x*_^2^ + *β*_*y*_^2^ + *β*_*z*_^2^)^1/2^

where, *β*_*x*_ = *β*_*xxx*_ + *β*_*xyy*_ + *β*_*xzz*_, *β*_*y*_ = *β*_*yxx*_ + *β*_*yyy*_ + *β*_*yzz*_ and *β*_*z*_ = *β*_*zxx*_ + *β*_*zyy*_ + *β*_*zzz*_.

**Table 6:** The computed NLO properties for TNPR and TNPD1–TNPD6^a^

Systems	*μ*_total_	〈*α*〉 × 10^−22^	*β*_tot_ × 10^−27^	*γ*_tot_ × 10^−32^
TNPR	2.5852	3.532	0.286	8.014
TNPD1	9.0370	3.491	3.420	6.168
TNPD2	10.5486	3.510	3.202	5.835
TNPD3	12.4305	3.494	3.717	6.944
TNPD4	9.2421	3.485	3.455	6.312
TNPD5	15.2931	3.613	4.653	9.472
TNPD6	9.5013	3.251	2.522	4.157

a *μ*_total_ units = Debye (D), while, 〈*α*〉, *β*_tot_ and *γ*_tot_ units = esu.

Second-hyper-polarizability (*γ*_tot_) was determined by employing eqn (6).^65^6
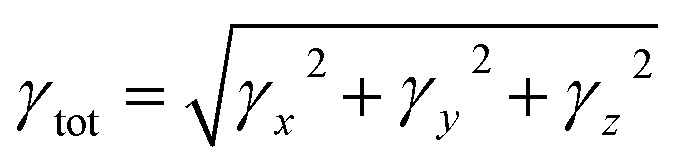
where, 
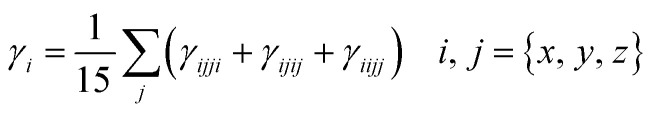
.

The dipole moments (*μ*_total_) arise due to differences in electronegativity (E.N.) between atoms, where a larger E.N. difference leads to a larger dipole moment.^66^ Furthermore, the polarity of molecules significantly influences the dipole moment, resulting in higher nonlinear values.^67^

According to Table 6, TNPD5 exhibits the highest dipole moment (*μ*_total_) of 15.2931 D among all the derivatives. The increased dipole moment in TNPD5 can be attributed to the presence of a cyano group, which is more electronegative than carbon. This electronegativity difference causes the bonded electrons to be pulled towards the cyano group, creating polarity in the molecule. The trend of decreasing dipole moments is as follows: TNPD5 > TNPD3 > TNPD2 > TNPD6 > TNPD4 > TNPD1 > TNPR.

The average linear polarizability 〈*α*〉 describes the linear response of the system. The examination of various acceptor groups and their impact on 〈α〉 provides valuable insights into the structural nonlinear optical properties in relation to the functional electric field. It is fascinating to observe and understand how different acceptor groups influence these properties.^68^ Table S36† provides the computed values of 〈*α*〉 and its corresponding tensor components in esu. The *α*_*xx*_ tensor component displays higher values, indicating polarization along the *x*-axis. Amongst the derivatives, TNPD5 exhibits the highest value of 〈*α*〉 at 3.613 × 10^−22^ esu, which is due to the presence of a cyano substituent in the acceptor moiety, which enhances its electron-withdrawing nature through inductive effects and extended conjugation. Decreasing order of 〈*α*〉 values for the molecules is as follows: TNPD5 > TNPR > TNPD2 > TNPD3 > TNPD1 > TNPD4 > TNPD6.

First hyper-polarizability (*β*_tot_), describes the NLO responses of chromophores, is calculated along with its contributing tensor components using the specified functional and basis set. Amongst all the derivatives, TNPD5 shows the highest (4.653 × 10^−27^ esu) *β*_tot_ amplitude due to the presence of 1-(dicyanomethylene)-3-oxo-2,3,3*a*,8*b*-tetrahydro-1*H*-benzo[*b*]cyclopenta[*d*]thiophene-6,7-dicarbonitrile acceptor group. A strong correlation is observed between the molecular structures and the values of *β*_tot_. The *β*_tot_ factor tends to increase with the electron-withdrawing (EWD) nature of the groups attached to the acceptor moieties, such as chloro (–Cl), nitro (NO_2_), trifluoromethyl (–CF_3_) radical and cyano (–CN), as they contribute to molecular nonlinearity. Moreover, the effect of extended conjugation on *β*_tot_ is also influenced by the substitution.^69^ The decreasing order of *β*_tot_ for all the designed chromophores is as follows: TNPD5 > TNPD3 > TNPD4 > TNPD1 > TNPD2 > TNPD6 > TNPR. Between the individual tensor components, *β*_*xxx*_ exhibits the highest values, indicating a more efficient charge transfer along the *x*-axis.

The second hyper-polarizability (*γ*_tot_) plays a crucial role in estimating the NLO response.^70^ Among all the derivatives, the maximum value of *γ*_tot_ is observed in TNPD5 (9.472 × 10^−32^ esu) which is due to the electron withdrawing nature of the end capped acceptor moiety. The charge transfer is increased in TNPD5 which is due to the presence of four electron withdrawing cyano (–CN) groups at the end capped acceptor moiety, which pulls the electron strongly. The decreasing order of *γ*_tot_ is: TNPD5 > TNPR > TNPD3 > TNPD4 > TNPD1 > TNPD2 > TNPD6. Between the individual tensor components, *γ*_*x*_ shows dominance and significantly higher values compared to other tensors, as shown in Table S36.† Notably, TNPD5 demonstrates the highest *γ*_*x*_ value of 9.307 × 10^−32^ esu amongst all the investigated molecules, indicating a strong charge shifting process along the *x*-axis and highlighting its pronounced diagonal tensor behavior. These findings suggest that the compounds' electron-accepting nature plays a crucial role in generating notable nonlinear responses.

## Conclusion

In summary, we fabricated a new series (TNPD1–TNPD6) of indacenodithiophene (IDIC) based derivatives from a synthesized non-fullerene acceptor molecule (IDT-BT-IC) for higher efficacy NLO materials. All the derivatives possessed lower energy gap than reference compound in following order TNPR (2.196 eV) > TNPD6 (2.079 eV) > TNPD2 (1.880 eV) > TNPD1 (1.866 eV) > TNPD4 (1.825 eV) > TNPD3 (1.807 eV) > TNPD5 (1.692 eV). All the designed structures (TNPD1–TNPD6) exhibited a high value of exciton dissociation owing to their lower *E*_b_ (0.236–0.294 eV) than TNPR. Furthermore, DOS and TDM analyses displayed a significant CT from the D to A region through the π-spacer which is also supported by FMOs (*E*_b_ = 0.442 eV). Among all the derivatives, TNPD5 displayed a maximum bathochromic shift (*λ*_max_ = 852.242 nm) with a lowest transition energy (*E* = 1.455 eV) in chloroform solvent. Due to these unique characteristics, TNPD5 exhibits the highest values for average linear polarizability 〈α〉 and non-linear hyper-polarizabilities (*β*_tot_ and *γ*_tot_) as 3.613 × 10^−22^, 4.653 × 10^−27^ and 9.472 × 10^−32^ esu, respectively among all the fabricated chromophores. In conclusion, structural tailoring with terminal electron withdrawing units can be utilized to obtained higher efficacy NLO materials.

## Data availability

Cartesian co-ordinates, UV-Vis data (wave lengths, excitation energies and oscillator strengths), NBOs analysis, dipole moments, linear polarizabilities with major contributing tensors, the first hyperpolarizabilities (*β*_tot_) and second hyperpolarizabilities (*γ*_tot_) with their contributing tensors, ChemDraw structures and their IUPAC names, optimized geometries, DOS plots and FMOs (HOMO−1, LUMO+1, HOMO−2, LUMO+2) of the reported compounds were calculated using M06/6-311G(d,p) and represented in ESI.†

## Conflicts of interest

There are no conflicts of interest to declare.

## Supplementary Material

RA-013-D3RA04858F-s001
