# Supplementary material for: Exploration of nonlinear optical enhancement in acceptor–π–donor indacenodithiophene based derivatives via structural variations: a DFT approach
Source: RSC Adv. 2023 Sep 22;13(40):28076–88. doi: 10.1039/d3ra04858f (PMC10517168; doi:10.1039/d3ra04858f)
Supplement: RA-013-D3RA04858F-s001 [file RA-013-D3RA04858F-s001.pdf]

Electronic Supplementary Material (ESI) for RSC Advances.
This journal is © The Royal Society of Chemistry 2023

Exploration of Nonlinear Optical Enhancement in Acceptor-π-Donor Indacenodithiophene Based Derivatives *via* Structural Variations: A DFT Approach

Saba Abid,[1,2] Muhammad Khalid,*[1,2] Muhammad Sagir,[3] Muhammad Imran,[4] Ataualpa A. C. Braga,[5] Suvash Chandra Ojha,*[6]

[1]Institute of Chemistry, Khwaja Fareed University of Engineering & Information Technology, Rahim Yar Khan, 64200, Pakistan

[2]Centre for Theoretical and Computational Research, Khwaja Fareed University of Engineering & Information Technology, Rahim Yar Khan, 64200, Pakistan

[3]Institute of Chemical and Environmental Engineering, Khwaja Fareed University of Engineering & Information Technology, Rahim Yar Khan, 64200, Pakistan

[4]Department of Chemistry, Faculty of Science, King Khalid University, P.O. Box 9004, Abha 61413, Saudi Arabia

[5]Departamento de Química Fundamental, Instituto de Química, Universidade de São Paulo, Av. Prof. Lineu Prestes, 748, São Paulo, 05508-000, Brazil

[6]Department of Infectious Diseases, The Affiliated Hospital of Southwest Medical University, Luzhou 646000, China

*Corresponding authors E-mail addresses:

Dr. Muhammad Khalid (muhammad.khalid@kfueit.edu.pk; Khalid@iq.usp.br)

Dr. Suvash Chandra Ojha (suvash_ojha@swmu.edu.cn)

*Corresponding authors E-mail addresses:

Dr. Muhammad Khalid (muhammad.khalid@kfueit.edu.pk; Khalid@iq.usp.br)

Table S1: Computed transition energy (*eV*), maximum absorption wavelengths (λ_{max}), oscillator strengths (f_{os}) and transition natures of compound **TNPR** in chloroform at B3LYP functional with 6-311G(d,p) basis set

NO.	λ_{DFT} (*nm*)	*E*(*eV*)	f_{os}	MO contributions
1	798.404	1.553	2.624	H→L (96%), H-1→L+1 (2%)
2	740.027	1.675	0.001	H→L+1 (99%),
3	598.293	2.072	0.006	H-1→L (98%),
4	584.418	2.122	1.183	H-1→L+1 (88%), H→L (3%), H→L+2 (5%), H→L+4 (2%)
5	564.026	2.198	0.138	H→L+2 (87%), H-1→L+1 (6%), H-1→L+3 (5%)
6	554.317	2.237	0.000	H→L+3 (93%), H-1→L+2 (5%)

Table S2: Computed transition energy (*eV*), maximum absorption wavelengths (λ_{max}), oscillator strengths (f_{os}) and transition natures of compound **TNPR** in chloroform at CAM-B3LYP functional with 6-311G(d,p) basis set

NO.	λ_{DFT} (*nm*)	*E*(*eV*)	f_{os}	MO contributions
1	484.446	2.559	3.611	H-2→L (13%), H-1→L+1 (31%), H→L (25%), H-7→L+1 (2%), H-2→L+1 (3%), H→L+1 (9%), H→L+2 (2%), H→L+4 (4%)
2	472.051	2.627	0.191	H-2→L+1 (15%), H-1→L (38%), H→L+1 (23%), H-7→L (3%), H-2→L (5%), H→L (6%)
3	378.844	3.273	0.212	H-2→L (15%), H→L+4 (35%), H-7→L+1 (6%), H-1→L+1 (7%), H-1→L+3 (2%), H→L (8%), H→L+2 (9%)
4	361.186	3.433	0.019	H-2→L+1 (13%), H-1→L+2 (10%), H→L+1 (17%), H→L+3 (13%), H-7→L (8%), H-2→L (3%), H-2→L+3 (5%), H-1→L (4%), H→L (8%), H→L+5 (3%)
5	353.302	3.509	0.055	H-2→L+3 (13%), H-1→L+3 (16%), H→L+1 (23%), H-7→L+3 (3%), H-2→L+1 (3%), H-2→L+2 (5%), H-1→L+2 (7%), H→L+2 (4%), H→L+3 (8%), H→L+4 (3%)
6	352.508	3.517	0.109	H-2→L+2 (18%), H-1→L+2 (17%), H-1→L+3 (12%), H→L (16%), H→L+2 (10%), H-7→L+2 (3%), H-7→L+3 (2%), H-2→L+3 (4%), H→L+1 (3%), H→L+3 (2%)

Table S3: Computed transition energy (*eV*), maximum absorption wavelengths (λ_{max}), oscillator strengths (f_{os}) and transition natures of compound **TNPR** in chloroform at M06 functional with 6-311G(d,p) basis set

NO.	λ_{DFT} (*nm*)	*E*(*eV*)	f_{os}	MO contributions
1	706.785	1.754	2.960	H→L (86%), H-1→L+1 (8%)
2	658.895	1.882	0.016	H→L+1 (89%), H-1→L (7%)
3	538.757	2.301	0.064	H-1→L (83%), H-2→L+1 (5%), H→L+1 (7%)
4	525.513	2.359	0.921	H-1→L+1 (75%), H→L (10%), H-2→L (5%), H→L+2 (3%), H→L+4 (2%)
5	502.530	2.467	0.166	H-1→L+3 (10%), H→L+2 (77%), H-1→L+1 (6%)
6	493.214	2.514	0.002	H-1→L+2 (12%), H→L+3 (82%),

Table S4: Computed transition energy (*eV*), maximum absorption wavelengths (λ_{max}), oscillator strengths (f_{os}) and transition natures of compound **TNPR** in chloroform at MPW1PW91 functional with 6-311G(d,p) basis set

NO.	λ_{DFT} (*nm*)	*E*(*eV*)	f_{os}	MO contributions
1	782.926	1.584	3.495	H→L (93%), H-1→L+1 (5%)
2	698.660	1.775	0.001	H→L+1 (96%), H-1→L (3%)
3	573.258	2.163	0.001	H-1→L (93%), H-2→L+1 (3%), H→L+1 (3%)
4	557.583	2.224	0.432	H→L+2 (82%), H-1→L+1 (6%), H-1→L+3 (7%), H→L (3%)
5	546.668	2.268	0.001	H→L+3 (89%), H-1→L+2 (8%)
6	544.053	2.279	0.419	H-1→L+1 (82%), H→L (4%), H→L+2 (7%), H→L+4 (2%)

Table S5: Computed transition energy (*eV*), maximum absorption wavelengths (λ_{max}), oscillator strengths (f_{os}) and transition natures of compound **TNPR** in chloroform at ωB97XD functional with 6-311G(d,p) basis set

NO.	λ_{DFT} (*nm*)	*E*(*eV*)	f_{os}	MO contributions

1	454.638	2.727	3.145	H-2→L (15%), H-1→L+1 (33%), H→L (11%), H→L+1 (10%), H-7→L+1 (5%), H-2→L+1 (8%), H-1→L+3 (2%), H→L+4 (4%)
2	444.420	2.790	0.607	H-2→L (12%), H-2→L+1 (18%), H-1→L (38%), H-7→L (5%), H-1→L+2 (2%), H→L (4%), H→L+1 (9%)
3	362.527	3.420	0.560	H→L+4 (52%), H-7→L+1 (5%), H-2→L (7%), H-2→L+4 (2%), H-1→L+1 (3%), H-1→L+3 (2%), H-1→L+5 (3%), H→L (2%), H→L+2 (6%)
4	342.706	3.618	0.029	H-2→L+3 (20%), H-1→L+2 (23%), H→L+3 (12%), H-24→L+2 (3%), H-23→L+3 (4%), H-20→L+1 (3%), H-7→L (3%), H-7→L+2 (3%), H-2→L+1 (3%), H-1→L+3 (3%)
5	338.477	3.663	0.054	H-2→L+2 (24%), H-1→L+3 (21%), H-24→L+3 (4%), H-23→L+2 (4%), H-23→L+3 (2%), H-20→L (2%), H-7→L+3 (5%), H-1→L+2 (4%), H→L+2 (5%), H→L+4 (7%)
6	335.655	3.694	0.007	H-24→L+2 (24%), H-24→L+3 (12%), H-23→L+2 (11%), H-26→L+2 (2%), H-24→L (5%), H-23→L+3 (5%), H-21→L+2 (7%), H-21→L+3 (4%)

Table S6: Cartesian coordinates of **TNPR**

Atom	X-axis	Y-axis	Z-axis
C	-0.77926	-1.23708	0.545443
C	0.59375	-1.36381	0.50919
C	1.344184	-0.2005	0.352007
C	0.732246	1.062195	0.235569
C	-0.63951	1.191972	0.303464
C	-1.38987	0.028736	0.462267
H	1.076002	-2.33631	0.573084
H	-1.12284	2.162399	0.219167
C	2.764793	0.017902	0.239744
C	3.054492	1.33837	0.017987
C	4.433136	1.607114	-0.14494
C	5.178168	0.446155	-0.02148
S	4.169785	-0.96411	0.289877
C	-5.23504	-0.6436	0.610001
C	-4.49275	-1.81171	0.673955
C	-3.10828	-1.53009	0.616518
C	-2.81269	-0.19579	0.517943
S	-4.21696	0.786814	0.467677
C	-6.66059	-0.43459	0.643906
C	-7.33176	0.681212	1.098364
S	-7.78459	-1.59541	-0.00379
C	-8.71962	0.603712	0.943977
H	-6.82376	1.519592	1.56112
C	-9.15284	-0.57289	0.369073
H	-9.40232	1.377059	1.278014
C	6.602044	0.235529	-0.10091

C	7.259496	-0.90405	-0.51408
S	7.742881	1.427892	0.44934
C	8.650855	-0.81733	-0.4108
H	6.737008	-1.77238	-0.89907
C	9.1035	0.388197	0.08688
H	9.319248	-1.61875	-0.70535
C	-1.82798	-2.35268	0.599817
C	1.774239	2.160017	-0.01055
C	-10.5077	-0.9423	0.072357
C	-11.0567	-2.20527	-0.15765
S	-11.6956	0.323279	-0.01926
C	-12.4283	-2.12051	-0.37209
C	-12.9695	-0.83737	-0.32968
H	-13.055	-2.99081	-0.54596
C	10.46897	0.79071	0.265655
C	10.98644	1.940359	0.86815
S	11.72192	-0.26644	-0.31894
C	12.37684	1.93244	0.849891
C	12.96818	0.821337	0.25422
H	12.97841	2.731421	1.274262
C	-10.3129	-3.50019	-0.14767
H	-9.65163	-3.59911	-1.01559
H	-9.69443	-3.60801	0.749154
H	-11.0144	-4.3372	-0.17461
C	10.19287	3.049333	1.475991
H	9.587099	3.576201	0.730597
H	9.512851	2.686983	2.25449
H	10.8609	3.782205	1.933968
C	-5.06432	-3.18203	0.834866
H	-6.02309	-3.16199	1.359368
H	-5.22352	-3.66952	-0.13459
H	-4.38271	-3.8157	1.409187
C	4.992742	2.952488	-0.47073
H	5.153966	3.554003	0.431938
H	5.946706	2.877555	-0.9983
H	4.300848	3.50774	-1.11049
C	-1.8212	-3.17805	-0.69435
C	-2.34741	-4.46378	-0.73389
C	-1.36515	-2.5941	-1.8788
C	-2.40357	-5.15636	-1.93993
H	-2.70506	-4.93807	0.173967
C	-1.4193	-3.29217	-3.07967
H	-0.96242	-1.58804	-1.85579

C	-1.94297	-4.58485	-3.10936
H	-2.81058	-6.16273	-1.96127
H	-1.9715	-5.1147	-4.05604
C	-1.59222	-3.16767	1.86668
C	-0.64192	-4.19367	1.888331
C	-2.23853	-2.82944	3.044759
C	-0.36915	-4.85722	3.070847
H	-0.1269	-4.47986	0.976571
C	-1.96278	-3.50256	4.236535
H	-2.97437	-2.03006	3.073402
C	-1.02209	-4.52685	4.253551
H	0.367091	-5.65525	3.080443
C	1.462534	2.742233	-1.38956
C	0.523172	3.767335	-1.5384
C	2.008067	2.165137	-2.52577
C	0.161931	4.194641	-2.80352
H	0.079602	4.233447	-0.66418
C	1.636322	2.59383	-3.80113
H	2.731595	1.357063	-2.45506
C	0.708646	3.620082	-3.94591
H	-0.56483	4.994086	-2.91188
H	0.407535	3.975209	-4.92425
C	1.827436	3.189354	1.119535
C	2.334502	4.474896	0.909661
C	1.463998	2.818601	2.405462
C	2.457771	5.352777	1.972343
H	2.624235	4.793436	-0.08599
C	1.591329	3.70424	3.475817
H	1.072393	1.82752	2.616914
C	2.089934	4.985211	3.26143
H	2.847431	6.351459	1.799448
O	-0.98051	-2.79963	-4.26078
O	2.230933	1.945489	-4.82847
C	-0.42811	-1.50052	-4.27949
H	-1.16246	-0.7449	-3.97356
H	0.453954	-1.42486	-3.63022
C	1.889126	2.3308	-6.14234
H	2.468342	1.694287	-6.81044
H	0.820511	2.180566	-6.3401
C	14.372	0.714781	0.186056
C	15.23871	-0.22326	-0.31747
H	14.8296	1.586956	0.644562
C	-14.3532	-0.64188	-0.51567

C	-15.1694	0.461941	-0.53504
H	-14.842	-1.59813	-0.68111
C	16.09885	-2.1673	-1.34476
C	16.23926	-3.38156	-1.98467
C	17.526	-3.84386	-2.22815
C	18.62594	-3.0891	-1.83066
C	18.47835	-1.86541	-1.18625
C	17.19191	-1.39621	-0.93876
H	15.35969	-3.94441	-2.28113
H	17.67916	-4.79411	-2.72864
H	19.62601	-3.46147	-2.02679
H	19.36194	-1.31324	-0.89546
C	-18.2825	2.486128	-0.93698
C	-17.0386	1.8884	-0.75782
C	-15.9198	2.700325	-0.55078
C	-15.9939	4.077418	-0.5123
C	-17.2385	4.667175	-0.69076
C	-18.363	3.874256	-0.90119
H	-19.1826	1.910291	-1.10493
H	-15.0965	4.665376	-0.34731
H	-17.3393	5.747106	-0.66801
H	-19.3293	4.347321	-1.04162
C	16.69107	-0.16498	-0.2914
C	14.84877	-1.47787	-0.98133
C	-14.7217	1.85728	-0.39752
C	-16.6096	0.473666	-0.7335
C	17.49788	0.824232	0.223744
C	-17.4631	-0.59919	-0.85523
O	13.72424	-1.88355	-1.19798
O	-13.5909	2.258128	-0.20549
C	17.01787	2.010891	0.840564
N	16.66979	2.989347	1.348564
C	18.91756	0.770614	0.195485
N	20.0733	0.775766	0.198388
C	-17.0551	-1.95866	-0.78875
N	-16.7655	-3.07671	-0.73749
C	-18.8647	-0.46626	-1.04731
N	-20.0083	-0.41202	-1.20515
H	-0.12806	-1.30944	-5.30944
H	2.146062	3.379813	-6.33385
H	-0.79245	-5.06716	5.164217
O	-2.66272	-3.08471	5.315143
C	-2.42544	-3.72631	6.549192

H	-3.07876	-3.24585	7.276739
H	-2.66774	-4.79505	6.500112
H	-1.38309	-3.60842	6.869939
H	2.193597	5.693962	4.074375
O	1.201086	3.223905	4.678323
C	1.305358	4.075613	5.798111
H	0.685913	4.973313	5.680233
H	0.946183	3.504789	6.653798
H	2.34441	4.37748	5.97901

Table S7: Cartesian coordinates of **TNPD1**

Atom	X-axis	Y-axis	Z-axis
C	-0.16107	-0.59366	1.714775
C	1.216395	-0.64691	1.698085
C	1.895584	0.453056	1.178532
C	1.2061	1.581706	0.698193
C	-0.1696	1.661179	0.777658
C	-0.84936	0.559835	1.292949
H	1.752925	-1.53645	2.020286
H	-0.70875	2.531069	0.409846
C	3.294912	0.681803	0.916978
C	3.494204	1.856209	0.240136
C	4.839209	2.087992	-0.12916
C	5.655648	1.060098	0.313219
S	4.751783	-0.18622	1.17035
C	-4.6477	-0.31058	1.60091
C	-3.83417	-1.36904	1.960837
C	-2.46778	-1.01626	1.841576
C	-2.25691	0.267095	1.412061
S	-3.725	1.103576	1.105679
C	-6.08918	-0.21056	1.562006
C	-6.87045	0.884117	1.838191
S	-7.08358	-1.53905	1.021651
C	-8.24622	0.660411	1.648971
H	-6.45512	1.817993	2.201144
C	-8.54867	-0.61006	1.219571
H	-9.00797	1.405212	1.851984
C	7.077994	0.869856	0.179724
C	7.762599	-0.32674	0.139616
S	8.192817	2.206099	0.151367
C	9.150539	-0.17725	0.067414

H	7.261026	-1.28777	0.145108
C	9.574286	1.136113	0.051871
H	9.836098	-1.01584	0.016289
C	-1.13629	-1.73375	2.013005
C	2.166795	2.567222	0.021389
C	-9.84972	-1.15412	0.909653
C	-10.2976	-2.45763	0.882381
S	-11.1344	-0.04383	0.49197
C	-11.6652	-2.54328	0.528497
C	-12.2746	-1.34192	0.286507
H	-12.1818	-3.49413	0.440119
C	10.92442	1.605543	-0.07309
C	11.43337	2.900165	0.047144
S	12.17075	0.439546	-0.41488
C	12.81385	2.915377	-0.13027
C	13.40299	1.681527	-0.38586
H	13.40761	3.823056	-0.07005
C	-9.47638	-3.66898	1.186448
H	-8.8201	-3.93908	0.350779
H	-8.8395	-3.52225	2.064324
H	-10.1248	-4.5272	1.380958
C	10.64164	4.131561	0.340894
H	9.943326	4.372341	-0.46829
H	10.05504	4.030677	1.260448
H	11.30886	4.987531	0.464415
C	-4.32877	-2.68815	2.458365
H	-5.31911	-2.59994	2.912025
H	-4.3963	-3.42637	1.650194
H	-3.64803	-3.09349	3.212817
C	5.293865	3.247928	-0.95197
H	5.491539	4.132648	-0.33492
H	6.20546	3.01715	-1.50847
H	4.522452	3.523959	-1.67677
C	-1.05025	-2.79986	0.908673
C	-1.56229	-4.07756	1.104676
C	-0.53332	-2.46227	-0.34484
C	-1.54928	-5.00044	0.063138
H	-1.96266	-4.36411	2.071646
C	-0.51001	-3.39234	-1.37745
H	-0.14014	-1.46633	-0.51265
C	-1.027	-4.67135	-1.17267
H	-1.94778	-5.9973	0.22596
H	-0.99741	-5.38447	-1.99024

C	-0.87028	-2.25874	3.416962
C	0.112533	-3.22633	3.646996
C	-1.52495	-1.69709	4.501831
C	0.409542	-3.61352	4.941712
H	0.634878	-3.68272	2.811796
C	-1.22487	-2.09138	5.806529
H	-2.28635	-0.93366	4.363301
C	-0.25095	-3.05899	6.032449
H	1.171274	-4.3678	5.114649
C	1.737656	2.589293	-1.44749
C	0.768311	3.487206	-1.90324
C	2.186309	1.596339	-2.3058
C	0.276935	3.377182	-3.19235
H	0.39527	4.268147	-1.24825
C	1.67766	1.479432	-3.59901
H	2.931367	0.871523	-1.98675
C	0.717076	2.377681	-4.05277
H	-0.47416	4.079463	-3.54106
H	0.310106	2.311455	-5.05466
C	2.211089	3.932465	0.704481
C	2.653984	5.071876	0.026184
C	1.90497	4.037668	2.052818
C	2.772128	6.275748	0.697682
H	2.897393	5.020473	-1.02961
C	2.027937	5.251851	2.729109
H	1.563384	3.177055	2.621095
C	2.462709	6.384551	2.048702
H	3.111392	7.156985	0.161564
O	0.00335	-3.14262	-2.60427
O	2.171928	0.450584	-4.32788
C	0.62972	-1.89721	-2.83224
H	-0.08109	-1.06153	-2.76878
H	1.449442	-1.71971	-2.12257
C	1.667008	0.262959	-5.63251
H	2.174918	-0.61278	-6.03646
H	0.584875	0.077747	-5.61993
C	14.79608	1.578353	-0.58711
C	15.64695	0.537795	-0.84868
H	15.25908	2.559559	-0.51151
C	16.52652	-1.60907	-1.25304
C	16.91167	-2.95365	-1.48314
C	18.30839	-3.03364	-1.67955
C	17.56177	-0.7209	-1.27301

C	17.08816	0.638788	-1.03158
C	15.27355	-0.888	-0.99137
C	17.91456	1.736486	-1.00545
O	14.1658	-1.37766	-0.91192
C	17.47662	3.067482	-0.78015
N	17.1497	4.161103	-0.5999
C	19.31253	1.600623	-1.21338
N	20.45069	1.491407	-1.38217
H	1.040347	-1.93921	-3.84036
H	1.876601	1.127109	-6.27429
H	-0.00186	-3.38507	7.035242
O	-1.93479	-1.47558	6.778906
C	-1.67469	-1.83419	8.118308
H	-2.34096	-1.23055	8.733744
H	-1.88356	-2.89596	8.29854
H	-0.63575	-1.62005	8.397852
H	2.561577	7.340751	2.548539
O	1.698209	5.22323	4.040379
C	1.801464	6.422117	4.77665
H	1.139324	7.199382	4.375501
H	1.496267	6.185224	5.795361
H	2.831539	6.799286	4.789333
C	18.94032	-4.24994	-1.92485
C	16.13778	-4.11395	-1.53429
H	20.01267	-4.30489	-2.07473
H	15.06491	-4.06247	-1.38477
S	19.09544	-1.46901	-1.57748
C	18.16658	-5.38837	-1.97309
C	16.77193	-5.32151	-1.77883
C	-13.6589	-1.0871	-0.08978
C	-14.653	-2.03563	0.179151
C	-14.0484	0.092021	-0.73293
C	-15.9677	-1.82423	-0.18567
H	-14.3957	-2.94464	0.71489
C	-15.3614	0.309391	-1.10442
H	-13.3041	0.84608	-0.97546
C	-16.3433	-0.64912	-0.84295
H	-16.7206	-2.56912	0.052229
H	-15.6353	1.22693	-1.61736
N	-17.6779	-0.40745	-1.21382
C	-18.199	0.90808	-1.05066
C	-18.5926	1.658441	-2.19345
C	-18.3083	1.446739	0.206546

C	-19.1426	2.956292	-1.99527
C	-18.8315	2.739348	0.392394
H	-17.9907	0.854044	1.060164
C	-19.2527	3.472416	-0.68328
H	-18.9123	3.142866	1.39654
H	-19.6703	4.466566	-0.54811
C	-18.5399	-1.43914	-1.63674
C	-18.0558	-2.50569	-2.40033
C	-19.9032	-1.3853	-1.34189
C	-18.9146	-3.50125	-2.83466
H	-17.0015	-2.54457	-2.65781
C	-20.7569	-2.38023	-1.79488
H	-20.2915	-0.55816	-0.75528
C	-20.2713	-3.44699	-2.5375
H	-18.5198	-4.32127	-3.42701
H	-21.8143	-2.32099	-1.555
H	-20.9418	-4.22567	-2.88576
C	-18.4317	1.178382	-3.51405
C	-19.544	3.708581	-3.12397
H	-17.9866	0.200088	-3.66893
H	-19.9703	4.69557	-2.96395
C	-19.3918	3.211377	-4.38913
C	-18.8211	1.937463	-4.58462
H	-19.7015	3.799061	-5.24755
H	-18.6874	1.55926	-5.59326
C	16.00474	-6.52362	-1.83582
N	15.38945	-7.49857	-1.88276
H	18.62725	-6.35166	-2.16214

Table S8: Cartesian coordinates of **TNPD2**

**Atom**	**X-axis**	**Y-axis**	**Z-axis**
C	0.579365	0.282831	1.705966
C	-0.79901	0.290462	1.734736
C	-1.4611	-0.74311	1.075562
C	-0.75383	-1.76203	0.411033
C	0.625542	-1.8052	0.440788
C	1.287856	-0.77154	1.098931
H	-1.35165	1.103038	2.200889
H	1.178562	-2.59084	-0.06886
C	-2.86096	-0.98131	0.82485
C	-3.04697	-2.04937	-0.01264
C	-4.39652	-2.27421	-0.3702

C	-5.22859	-1.35285	0.243612
S	-4.33483	-0.21412	1.248406
C	5.067734	0.152517	1.46828
C	4.234973	1.126872	1.986216
C	2.875788	0.758059	1.835709
C	2.689393	-0.45478	1.227406
S	4.171928	-1.19911	0.784853
C	6.51061	0.091769	1.405567
C	7.316584	-1.0111	1.540876
S	7.471743	1.497799	1.02459
C	8.684975	-0.73677	1.369944
H	6.923709	-1.99082	1.790097
C	8.958192	0.582489	1.09412
H	9.462598	-1.48548	1.474459
C	-6.65995	-1.19325	0.180591
C	-7.37794	-0.0254	0.32764
S	-7.73799	-2.54798	0.001232
C	-8.7634	-0.20772	0.277402
H	-6.90261	0.940919	0.452056
C	-9.15059	-1.51905	0.092694
H	-9.47389	0.606675	0.36559
C	1.529817	1.398724	2.144098
C	-1.70607	-2.66662	-0.38097
C	10.24724	1.182365	0.846139
C	10.65761	2.497782	0.902198
S	11.57449	0.134532	0.399707
C	12.02999	2.641907	0.587749
C	12.6802	1.473883	0.294626
H	12.51952	3.61069	0.565151
C	-10.4909	-2.00974	-0.06072
C	-10.965	-3.32211	-0.06405
S	-11.7705	-0.8483	-0.26704
C	-12.3485	-3.3555	-0.21932
C	-12.9717	-2.1183	-0.34042
H	-12.9191	-4.27967	-0.23798
C	9.794165	3.667221	1.249675
H	9.11339	3.931546	0.431935
H	9.17847	3.474095	2.134067
H	10.41109	4.545437	1.45615
C	-10.1375	-4.5549	0.09349
H	-9.46809	-4.71056	-0.75979
H	-9.51612	-4.51773	0.994592
H	-10.7818	-5.43375	0.16925

C	4.70702	2.372075	2.663705
H	5.707031	2.243934	3.085652
H	4.744073	3.220753	1.97022
H	4.030301	2.646587	3.478686
C	-4.84213	-3.31843	-1.33983
H	-4.96582	-4.29526	-0.8571
H	-5.79128	-3.05492	-1.81248
H	-4.09836	-3.44142	-2.13274
C	1.37643	2.612144	1.21271
C	1.857064	3.8645	1.578898
C	0.826446	2.444591	-0.06066
C	1.778249	4.929266	0.686082
H	2.28193	4.02081	2.564999
C	0.736589	3.514638	-0.94289
H	0.459148	1.471313	-0.36366
C	1.221316	4.767287	-0.56771
H	2.152113	5.904763	0.982134
H	1.139902	5.591481	-1.26905
C	1.294842	1.699877	3.617934
C	0.298663	2.594633	4.019965
C	1.995612	0.997865	4.586232
C	0.034018	2.772572	5.366491
H	-0.26029	3.157815	3.279062
C	1.727945	1.18093	5.943479
H	2.768991	0.28485	4.312022
C	0.740368	2.07669	6.3412
H	-0.73858	3.471124	5.673472
C	-1.33169	-2.44141	-1.84795
C	-0.33799	-3.20822	-2.46334
C	-1.85929	-1.35657	-2.53216
C	0.099501	-2.88017	-3.73447
H	0.09561	-4.05796	-1.94566
C	-1.40606	-1.021	-3.80787
H	-2.62626	-0.72856	-2.0855
C	-0.42043	-1.78809	-4.42041
H	0.870299	-3.48111	-4.20743
H	-0.05457	-1.55112	-5.41226
C	-1.6778	-4.12301	0.077064
C	-2.09813	-5.15887	-0.76239
C	-1.32488	-4.4262	1.383299
C	-2.14825	-6.45769	-0.28807
H	-2.37665	-4.95192	-1.79017
C	-1.37823	-5.7362	1.860484

H	-0.99884	-3.65187	2.072038
C	-1.79091	-6.76493	1.019826
H	-2.4704	-7.2571	-0.94855
O	0.185256	3.428534	-2.17562
O	-1.97806	0.076326	-4.35803
C	-0.40941	2.207337	-2.56371
H	0.330258	1.399308	-2.65124
H	-1.19268	1.894922	-1.85927
C	-1.53939	0.478356	-5.63794
H	-2.10663	1.374035	-5.89087
H	-0.46819	0.719065	-5.63661
C	-14.3721	-2.03098	-0.5008
C	-15.2513	-0.99163	-0.64206
H	-14.8108	-3.02618	-0.51039
C	-16.1841	1.161391	-0.84053
C	-16.6022	2.510313	-0.94577
C	-18.0023	2.573869	-1.10395
C	-17.202	0.253962	-0.91379
C	-16.6954	-1.10894	-0.79688
C	-14.9116	0.449791	-0.6685
C	-17.4981	-2.22404	-0.83484
O	-13.8122	0.955319	-0.57153
C	-17.028	-3.55813	-0.72084
N	-16.6753	-4.65493	-0.63071
C	-18.9035	-2.10382	-0.99657
N	-20.0476	-2.00681	-1.12844
H	-0.86293	2.382151	-3.53863
H	-1.73346	-0.29428	-6.39156
H	0.515413	2.239839	7.388461
O	2.481345	0.443544	6.790449
C	2.252968	0.58441	8.175467
H	2.951451	-0.08786	8.672721
H	2.441927	1.611177	8.512362
H	1.228223	0.300663	8.445296
H	-1.83605	-7.79141	1.363703
O	-1.00682	-5.90126	3.15039
C	-1.03583	-7.20556	3.687123
H	-0.35725	-7.87798	3.147816
H	-0.70504	-7.12035	4.721706
H	-2.04872	-7.6265	3.667965
C	-18.6581	3.79039	-1.22948
C	-15.8557	3.69074	-0.91392
H	-19.7334	3.850491	-1.35172

H	-14.7789	3.655201	-0.79273
S	-18.7581	0.988033	-1.11849
C	-17.9047	4.948	-1.1959
C	-16.5055	4.897915	-1.03837
Cl	-18.7255	6.467777	-1.35289
Cl	-15.5668	6.358183	-0.99792
C	14.07955	1.276314	-0.05904
C	15.03884	2.241771	0.2692
C	14.51871	0.133338	-0.73521
C	16.36807	2.079745	-0.06771
H	14.74251	3.123309	0.830068
C	15.84636	-0.03454	-1.07852
H	13.80281	-0.63144	-1.02483
C	16.7936	0.939337	-0.75489
H	17.09326	2.835789	0.216694
H	16.15986	-0.92453	-1.61679
N	18.14455	0.744887	-1.09442
C	18.7005	-0.55688	-0.93724
C	19.16914	-1.26716	-2.07741
C	18.77157	-1.12142	0.311418
C	19.75244	-2.55105	-1.88305
C	19.32791	-2.40082	0.492102
H	18.39749	-0.55953	1.162981
C	19.8205	-3.0945	-0.5793
H	19.37701	-2.8251	1.489723
H	20.26399	-4.07783	-0.4476
C	18.98946	1.813527	-1.45545
C	18.49964	2.887252	-2.20523
C	20.34229	1.79196	-1.11275
C	19.34197	3.920403	-2.5798
H	17.45443	2.901971	-2.49949
C	21.18037	2.824775	-1.50647
H	20.735	0.959956	-0.53602
C	20.68846	3.897851	-2.23567
H	18.94312	4.745474	-3.16234
H	22.22994	2.7902	-1.23023
H	21.34643	4.706081	-2.53737
C	19.05233	-0.76135	-3.39302
C	20.22928	-3.26297	-3.00853
H	18.58247	0.20546	-3.54651
H	20.68044	-4.23923	-2.85094
C	20.1184	-2.74094	-4.26794
C	19.51495	-1.48185	-4.46115

H	20.48613	-3.29774	-5.12405
H	19.41455	-1.08428	-5.4662

Table S9: Cartesian coordinates of **TNPD3**

Atom	X-axis	Y-axis	Z-axis
C	1.237842	0.120906	1.669051
C	-0.14127	0.137101	1.662916
C	-0.79433	-0.82623	0.896235
C	-0.07947	-1.78615	0.155774
C	1.299696	-1.82048	0.185273
C	1.952435	-0.85747	0.951448
H	-0.70258	0.889146	2.21234
H	1.861207	-2.55863	-0.38261
C	-2.19327	-1.06649	0.640151
C	-2.37224	-2.09343	-0.24894
C	-3.72449	-2.34801	-0.57266
C	-4.56559	-1.49061	0.117126
S	-3.67722	-0.37512	1.15166
C	5.730764	-0.04504	1.535117
C	4.894696	0.848386	2.179169
C	3.536056	0.524584	1.943938
C	3.352465	-0.5776	1.15294
S	4.837308	-1.2644	0.633022
C	7.173496	-0.11966	1.490047
C	7.954356	-1.24697	1.417552
S	8.172743	1.309933	1.416637
C	9.332033	-0.97785	1.330921
H	7.537066	-2.24717	1.459766
C	9.636424	0.362016	1.333381
H	10.09406	-1.74889	1.299951
C	-6.00363	-1.39292	0.115598
C	-6.77159	-0.27127	0.348099
S	-7.01906	-2.79129	-0.08581
C	-8.14737	-0.52227	0.343312
H	-6.33887	0.710626	0.50259
C	-8.47572	-1.84268	0.112399
H	-8.89378	0.248555	0.500186
C	2.19104	1.119823	2.334285
C	-1.02732	-2.67524	-0.65771
C	10.94159	0.971867	1.229299
C	11.40996	2.181615	1.693782
S	12.20219	0.089481	0.399747

C	12.77656	2.378921	1.380271
C	13.36227	1.352983	0.689554
H	13.31083	3.283536	1.6545
C	-9.79232	-2.39983	-0.01161
C	-10.1936	-3.73731	-0.01957
S	-11.1378	-1.30786	-0.16778
C	-11.5759	-3.84493	-0.13539
C	-12.2695	-2.6416	-0.22358
H	-12.0962	-4.79845	-0.14927
C	10.60905	3.191968	2.450121
H	9.985999	3.800561	1.784155
H	9.941489	2.721827	3.178649
H	11.27084	3.874514	2.989724
C	-9.2959	-4.92443	0.0998
H	-8.6561	-5.04434	-0.78146
H	-8.64019	-4.85479	0.974079
H	-9.88918	-5.83616	0.199826
C	5.356273	1.971442	3.049393
H	6.348985	1.777822	3.463326
H	5.402884	2.917852	2.497281
H	4.666306	2.11769	3.885832
C	-4.16929	-3.3619	-1.57459
H	-4.27038	-4.35792	-1.12694
H	-5.13072	-3.0961	-2.02075
H	-3.43759	-3.44305	-2.38361
C	2.085209	2.503539	1.680249
C	2.487302	3.653906	2.348167
C	1.667815	2.600876	0.350311
C	2.458832	4.883313	1.69589
H	2.814583	3.601773	3.381318
C	1.637352	3.831409	-0.29474
H	1.362619	1.704523	-0.17744
C	2.035819	4.982682	0.385249
H	2.769372	5.778387	2.226346
H	1.999985	5.934648	-0.13463
C	1.918251	1.108674	3.8345
C	0.878892	1.871168	4.377702
C	2.626861	0.256006	4.665677
C	0.58054	1.771766	5.724539
H	0.313188	2.550431	3.747491
C	2.324647	0.159096	6.025262
H	3.434064	-0.36153	4.280572
C	1.294583	0.922754	6.563622

H	-0.22524	2.36886	6.140828
C	-0.68498	-2.43315	-2.12766
C	0.329315	-3.16253	-2.75585
C	-1.28365	-1.3898	-2.81674
C	0.711356	-2.84355	-4.04659
H	0.815471	-3.98179	-2.2355
C	-0.89119	-1.06609	-4.11654
H	-2.06612	-0.78942	-2.35963
C	0.111621	-1.79955	-4.74231
H	1.496973	-3.41634	-4.53006
H	0.430728	-1.57184	-5.75237
C	-0.97795	-4.14151	-0.22433
C	-1.38416	-5.16761	-1.08244
C	-0.62243	-4.46388	1.076752
C	-1.4181	-6.4754	-0.63147
H	-1.66393	-4.94604	-2.10681
C	-0.65957	-5.78272	1.530172
H	-0.30669	-3.69892	1.780591
C	-1.05818	-6.80166	0.670943
H	-1.72929	-7.26675	-1.3068
O	1.229655	4.005204	-1.57328
O	-1.54236	-0.0215	-4.67734
C	0.8073	2.870248	-2.29893
H	1.61711	2.138431	-2.41052
H	-0.05266	2.381408	-1.82278
C	-1.18545	0.354794	-5.98967
H	-1.81564	1.205041	-6.24848
H	-0.13251	0.656932	-6.04916
C	-13.6737	-2.63233	-0.35174
C	-14.616	-1.64454	-0.46871
H	-14.0551	-3.65081	-0.36151
C	-15.6785	0.449448	-0.65024
C	-16.1845	1.768038	-0.75561
C	-17.5819	1.742605	-0.90556
C	-16.6402	-0.51728	-0.71461
C	-16.05	-1.84944	-0.60412
C	-14.3621	-0.18617	-0.49249
C	-16.7873	-3.00849	-0.63232
O	-13.2945	0.383743	-0.4045
C	-16.2384	-4.31297	-0.52594
N	-15.8203	-5.3869	-0.44182
C	-18.1995	-2.97024	-0.77393
N	-19.3491	-2.93992	-0.88929

H	0.511796	3.228065	-3.28491
H	-1.36694	-0.45714	-6.70458
H	1.042692	0.868808	7.616054
O	3.090663	-0.7033	6.731091
C	2.83039	-0.84497	8.110363
H	3.54402	-1.57814	8.484843
H	2.973779	0.101446	8.646288
H	1.812	-1.20993	8.292317
H	-1.09039	-7.83463	0.996404
O	-0.28731	-5.96592	2.817499
C	-0.29988	-7.27975	3.330903
H	0.388257	-7.93356	2.780914
H	0.028303	-7.20865	4.367393
H	-1.30709	-7.71365	3.302602
C	-18.3098	2.918314	-1.03513
C	-15.5236	2.998903	-0.73284
H	-19.3859	2.890931	-1.15324
H	-14.4471	3.018937	-0.61525
S	-18.239	0.117744	-0.90963
C	-17.648	4.130964	-1.01011
C	-16.2372	4.171009	-0.86258
C	14.74036	1.236784	0.230285
C	15.76434	1.94501	0.869774
C	15.09357	0.427666	-0.85415
C	17.07558	1.855394	0.44563
H	15.53249	2.551092	1.740869
C	16.40205	0.336958	-1.28811
H	14.32398	-0.122	-1.38983
C	17.41648	1.052804	-0.64724
H	17.85203	2.398154	0.97538
H	16.64757	-0.28673	-2.14282
N	18.74601	0.930285	-1.08658
C	19.20188	-0.34859	-1.51843
C	19.58146	-0.54024	-2.8758
C	19.26195	-1.38864	-0.6256
C	20.06502	-1.8197	-3.27001
C	19.72013	-2.65644	-1.02813
H	18.95615	-1.21523	0.402618
C	20.12573	-2.86381	-2.31823
H	19.76304	-3.46303	-0.30339
H	20.49297	-3.8364	-2.63481
C	19.66916	1.99298	-1.00681
C	19.263	3.311054	-1.23572

C	21.0166	1.737982	-0.7476
C	20.18201	4.345311	-1.18072
H	18.22193	3.518268	-1.46501
C	21.9315	2.779844	-0.71005
H	21.34444	0.717277	-0.57535
C	21.52291	4.089232	-0.9191
H	19.84767	5.362501	-1.36149
H	22.97554	2.561574	-0.50651
H	22.24086	4.901965	-0.88404
C	19.46933	0.478722	-3.85019
C	20.45152	-2.0195	-4.61597
H	19.07455	1.446992	-3.55739
H	20.827	-2.99693	-4.90767
C	20.34787	-1.00998	-5.53318
C	19.84285	0.24802	-5.14679
H	20.64555	-1.17614	-6.56367
H	19.74721	1.037092	-5.88605
C	-15.4533	5.45641	-0.77435
C	-18.4923	5.364919	-1.21093
F	-14.1445	5.24199	-0.92375
F	-15.8076	6.337565	-1.71096
F	-15.6124	6.04598	0.413766
F	-18.1877	6.338033	-0.35186
F	-18.3525	5.857188	-2.44439
F	-19.791	5.100726	-1.04838

Table S10: Cartesian coordinates of **TNPD4**

Atom	X-axis	Y-axis	Z-axis
C	-0.35046	-0.55719	1.603884
C	1.027157	-0.60497	1.586142
C	1.701218	0.493785	1.05732
C	1.006345	1.615016	0.56736
C	-0.36966	1.689004	0.646437
C	-1.04406	0.589673	1.172836
H	1.567633	-1.48907	1.916651
H	-0.91273	2.553829	0.272538
C	3.10006	0.729977	0.799505
C	3.294139	1.901583	0.116238
C	4.640538	2.145181	-0.24005
C	5.462744	1.129585	0.218928
S	4.563218	-0.12116	1.073707

C	-4.83863	-0.2816	1.524574
C	-4.02015	-1.33631	1.886279
C	-2.6554	-0.98459	1.747589
C	-2.44985	0.294271	1.302435
S	-3.92118	1.126567	1.00196
C	-6.27962	-0.17494	1.510253
C	-7.04475	0.942025	1.741439
S	-7.30037	-1.52252	1.074508
C	-8.42667	0.722154	1.599815
H	-6.61302	1.891124	2.039962
C	-8.75059	-0.56821	1.255583
H	-9.1775	1.483996	1.77875
C	6.890368	0.961483	0.112968
C	7.59592	-0.22282	0.080182
S	7.981689	2.316729	0.127004
C	8.982839	-0.04895	0.04819
H	7.110332	-1.19195	0.064777
C	9.383798	1.271664	0.057648
H	9.684199	-0.87513	0.010998
C	-1.32084	-1.69798	1.914644
C	1.962849	2.601264	-0.11425
C	-10.0646	-1.11709	1.013808
C	-10.5408	-2.4035	1.145992
S	-11.327	-0.03503	0.473761
C	-11.9142	-2.49898	0.815654
C	-12.4979	-1.3211	0.435684
H	-12.4553	-3.43986	0.847114
C	10.72892	1.766356	-0.01449
C	11.21061	3.065817	0.155925
S	12.00608	0.630186	-0.34154
C	12.59636	3.107321	0.033803
C	13.21565	1.889665	-0.22905
H	13.17245	4.021706	0.143937
C	-9.74247	-3.5865	1.590472
H	-9.13374	-3.99786	0.776639
H	-9.06169	-3.33569	2.409625
H	-10.4058	-4.3841	1.935106
C	10.38762	4.275908	0.451878
H	9.711845	4.523948	-0.37397
H	9.773229	4.14248	1.348834
H	11.03484	5.140065	0.617623
C	-4.5071	-2.65086	2.403176
H	-5.47529	-2.5527	2.900885

H	-4.61994	-3.38663	1.59776
H	-3.7987	-3.06537	3.126173
C	5.093165	3.304666	-1.06468
H	5.265066	4.197933	-0.45231
H	6.018938	3.082869	-1.60105
H	4.331749	3.562091	-1.80682
C	-1.2378	-2.76889	0.814452
C	-1.75118	-4.04519	1.016311
C	-0.72121	-2.43774	-0.44088
C	-1.73998	-4.97258	-0.02117
H	-2.15094	-4.32736	1.984793
C	-0.69948	-3.37232	-1.46943
H	-0.32678	-1.44323	-0.61365
C	-1.21804	-4.64968	-1.25883
H	-2.13961	-5.9682	0.146321
H	-1.18989	-5.36651	-2.07319
C	-1.04417	-2.21494	3.31942
C	-0.06248	-3.18397	3.547706
C	-1.68709	-1.64294	4.406032
C	0.245124	-3.56237	4.842647
H	0.450965	-3.64806	2.711245
C	-1.3764	-2.02834	5.71079
H	-2.44728	-0.87811	4.268607
C	-0.40354	-2.9975	5.935098
H	1.005998	-4.3178	5.014317
C	1.538473	2.607793	-1.58462
C	0.562264	3.493295	-2.05006
C	1.997993	1.611914	-2.43368
C	0.075068	3.368555	-3.33935
H	0.180528	4.276102	-1.4024
C	1.493107	1.479724	-3.72697
H	2.748334	0.896148	-2.10673
C	0.525895	2.365842	-4.19043
H	-0.68129	4.061358	-3.69555
H	0.121844	2.287634	-5.19264
C	1.995404	3.972448	0.557557
C	2.427551	5.110362	-0.13003
C	1.689547	4.085404	1.905361
C	2.535513	6.320498	0.531998
H	2.670728	5.052702	-1.18556
C	1.80207	5.305864	2.571973
H	1.356334	3.22618	2.48069
C	2.226128	6.437076	1.882324

H	2.866544	7.200488	-0.01127
O	-0.18603	-3.12831	-2.6974
O	1.99728	0.44869	-4.4459
C	0.441834	-1.88459	-2.93072
H	-0.26807	-1.04782	-2.87123
H	1.261589	-1.70464	-2.22171
C	1.498097	0.247057	-5.75065
H	2.013948	-0.62792	-6.14605
H	0.417363	0.053732	-5.74016
C	14.61839	1.810661	-0.35949
C	15.49711	0.789198	-0.60502
H	15.06295	2.794662	-0.22865
C	16.42619	-1.32613	-1.06179
C	16.8425	-2.65342	-1.33385
C	18.25024	-2.70914	-1.45148
C	17.44881	-0.428	-0.97799
C	16.94508	0.912795	-0.694
C	15.15069	-0.63186	-0.83519
C	17.75576	2.013074	-0.55153
O	14.04729	-1.1367	-0.84192
C	17.29044	3.323088	-0.26604
N	16.94156	4.399878	-0.03336
C	19.1649	1.90139	-0.68495
N	20.312	1.811221	-0.79388
H	0.852778	-1.93153	-3.93853
H	1.703211	1.107786	-6.39845
H	-0.14631	-3.31682	6.938019
O	-2.07526	-1.4028	6.684972
C	-1.80331	-1.75136	8.024671
H	-2.46165	-1.14054	8.641576
H	-2.01383	-2.81092	8.215628
H	-0.76104	-1.53833	8.292393
H	2.31675	7.397997	2.374619
O	1.473655	5.284676	3.883723
C	1.566541	6.490285	4.610326
H	0.897079	7.258287	4.20352
H	1.264231	6.258776	5.631132
H	2.593162	6.876884	4.619202
C	18.9135	-3.90465	-1.71921
C	16.08851	-3.8178	-1.48698
H	19.99333	-3.93896	-1.8083
H	15.00881	-3.80591	-1.40183
S	19.00822	-1.14511	-1.22576

C	18.1614	-5.04921	-1.86929
C	16.76944	-4.98391	-1.7506
C	-13.882	-1.07638	0.051098
C	-14.9036	-1.92001	0.502201
C	-14.2438	-0.00692	-0.77403
C	-16.221	-1.71094	0.144373
H	-14.6641	-2.73636	1.177521
C	-15.5586	0.205503	-1.14145
H	-13.4769	0.658376	-1.16189
C	-16.5709	-0.64558	-0.69062
H	-16.9947	-2.36799	0.528669
H	-15.8112	1.034334	-1.79647
N	-17.9065	-0.40051	-1.0539
C	-18.3532	0.950551	-1.12236
C	-18.7697	1.49266	-2.36981
C	-18.3709	1.722016	0.012098
C	-19.2455	2.833871	-2.40084
C	-18.821	3.054251	-0.03103
H	-18.0381	1.284152	0.949289
C	-19.2617	3.593982	-1.20845
H	-18.8299	3.644003	0.879882
H	-19.6232	4.61807	-1.24852
C	-18.8452	-1.4387	-1.22598
C	-18.467	-2.65581	-1.80068
C	-20.181	-1.24692	-0.87065
C	-19.4017	-3.65982	-1.98971
H	-17.4356	-2.80656	-2.10532
C	-21.1122	-2.25376	-1.07854
H	-20.4871	-0.30293	-0.43002
C	-20.731	-3.46766	-1.63197
H	-19.0892	-4.59829	-2.43777
H	-22.1469	-2.08585	-0.79518
H	-21.4613	-4.25465	-1.78843
C	-18.7022	0.762764	-3.57939
C	-19.6697	3.380529	-3.63462
H	-18.3132	-0.25083	-3.56245
H	-20.0391	4.40269	-3.64878
C	-19.6091	2.644755	-4.78612
C	-19.1113	1.326377	-4.75792
H	-19.9354	3.075995	-5.72722
H	-19.0496	0.757008	-5.68002
N	15.99397	-6.22604	-1.91548
O	16.60794	-7.24649	-2.14944

O	14.78751	-6.15714	-1.80772
H	18.62821	-6.00336	-2.07826

Table S11: Cartesian coordinates of **TNPD5**

Atom	X-axis	Y-axis	Z-axis
C	-0.41998	-0.38734	1.714931
C	0.958285	-0.40228	1.73556
C	1.621448	0.655459	1.116895
C	0.915416	1.704357	0.499361
C	-0.46352	1.752614	0.538961
C	-1.12682	0.695159	1.157117
H	1.509762	-1.23561	2.164869
H	-1.01557	2.56195	0.066774
C	3.020855	0.898745	0.870466
C	3.207826	2.002276	0.080029
C	4.556037	2.236849	-0.27351
C	5.387131	1.284425	0.294074
S	4.492264	0.105802	1.251303
C	-4.90889	-0.22294	1.513806
C	-4.07857	-1.2218	1.987247
C	-2.71813	-0.85468	1.842953
C	-2.52895	0.380882	1.28285
S	-4.00987	1.150213	0.87967
C	-6.35171	-0.1518	1.460821
C	-7.15017	0.949962	1.642068
S	-7.32305	-1.53583	1.028658
C	-8.52089	0.691122	1.465285
H	-6.75015	1.916696	1.927917
C	-8.80314	-0.61457	1.139078
H	-9.29361	1.439822	1.601437
C	6.815667	1.11842	0.213147
C	7.526628	-0.0591	0.325843
S	7.901574	2.467904	0.047156
C	8.911099	0.113032	0.259477
H	7.04563	-1.02428	0.43578
C	9.307168	1.425716	0.096717
H	9.615575	-0.70906	0.319997
C	-1.37347	-1.51572	2.111789
C	1.86761	2.637509	-0.25894
C	-10.0963	-1.19569	0.867974
C	-10.5197	-2.5078	0.88873
S	-11.4092	-0.12409	0.436255

C	-11.8907	-2.63064	0.559345
C	-12.5269	-1.44936	0.289084
H	-12.3894	-3.59367	0.509013
C	10.64826	1.905672	-0.06434
C	11.13386	3.216437	-0.0585
S	11.91547	0.736233	-0.296
C	12.51366	3.240266	-0.22632
C	13.12721	1.997791	-0.36781
H	13.09186	4.159759	-0.24026
C	-9.67005	-3.69333	1.215034
H	-8.99746	-3.955	0.389678
H	-9.04705	-3.52067	2.098421
H	-10.2974	-4.56628	1.41246
C	10.31711	4.453277	0.120228
H	9.634626	4.618084	-0.7208
H	9.71097	4.41453	1.031563
H	10.96835	5.327346	0.190365
C	-4.55428	-2.49025	2.617473
H	-5.55152	-2.37331	3.049082
H	-4.59923	-3.31079	1.891388
H	-3.8753	-2.80038	3.417735
C	5.001232	3.322526	-1.19648
H	5.157795	4.268973	-0.66512
H	5.933384	3.065959	-1.70564
H	4.242244	3.503513	-1.96301
C	-1.23537	-2.68823	1.126869
C	-1.72688	-3.94999	1.44263
C	-0.68777	-2.4726	-0.14035
C	-1.66175	-4.97732	0.506281
H	-2.14902	-4.14318	2.423345
C	-0.61135	-3.50623	-1.06643
H	-0.31174	-1.49076	-0.403
C	-1.10747	-4.76848	-0.74182
H	-2.0439	-5.96072	0.762977
H	-1.03632	-5.56346	-1.47718
C	-1.12599	-1.88623	3.567502
C	-0.12616	-2.79961	3.915205
C	-1.81761	-1.23335	4.575703
C	0.151632	-3.04393	5.24842
H	0.42472	-3.32591	3.141833
C	-1.53717	-1.48396	5.919708
H	-2.5935	-0.50797	4.344398
C	-0.54571	-2.39773	6.263033

H	0.927055	-3.75656	5.512985
C	1.489995	2.468628	-1.733
C	0.496407	3.258332	-2.31876
C	2.015456	1.409125	-2.45752
C	0.05895	2.978756	-3.60153
H	0.062754	4.08789	-1.76941
C	1.561518	1.121531	-3.74456
H	2.781107	0.762881	-2.03539
C	0.577584	1.912705	-4.32833
H	-0.71119	3.597967	-4.05143
H	0.211635	1.713905	-5.32852
C	1.843163	4.074428	0.257136
C	2.250393	5.145436	-0.54351
C	1.506969	4.321827	1.579527
C	2.303543	6.42355	-0.01585
H	2.515582	4.982679	-1.58263
C	1.563445	5.610722	2.110515
H	1.191853	3.518412	2.239472
C	1.962573	6.674871	1.308159
H	2.615146	7.250797	-0.64642
O	-0.06295	-3.3753	-2.29655
O	2.131004	0.043765	-4.33442
C	0.55057	-2.14846	-2.63397
H	-0.17573	-1.3254	-2.68576
H	1.340096	-1.87936	-1.9188
C	1.694277	-0.30797	-5.62976
H	2.259104	-1.19547	-5.91491
H	0.622302	-0.54487	-5.64031
C	14.52131	1.90159	-0.54269
C	15.39224	0.854066	-0.7079
H	14.96991	2.892469	-0.5434
C	16.30224	-1.30287	-0.95432
C	16.70719	-2.65188	-1.08811
C	18.10757	-2.72482	-1.26003
C	17.32797	-0.40632	-1.02076
C	16.83236	0.962528	-0.87443
C	15.03668	-0.58082	-0.75537
C	17.64953	2.066962	-0.90169
O	13.93585	-1.08127	-0.65553
C	17.19625	3.404326	-0.7601
N	16.85515	4.502548	-0.64746
C	19.05122	1.929975	-1.08012
N	20.19201	1.815094	-1.22579

H	0.999515	-2.28899	-3.61655
H	1.893026	0.492025	-6.35298
H	-0.31102	-2.61276	7.298711
O	-2.2828	-0.79046	6.809483
C	-2.043	-1.00204	8.183556
H	-2.73683	-0.35591	8.720346
H	-2.2299	-2.04467	8.468958
H	-1.01582	-0.73286	8.459041
H	2.009733	7.686175	1.694136
O	1.208923	5.720855	3.410923
C	1.242057	7.0018	4.001062
H	0.554944	7.694158	3.499065
H	0.925013	6.872673	5.035391
H	2.25371	7.425886	3.986359
C	18.75581	-3.94262	-1.41443
C	15.94666	-3.82344	-1.0719
H	19.82992	-4.00339	-1.54671
H	14.87155	-3.77438	-0.941
S	18.87475	-1.14947	-1.25239
C	17.99334	-5.09975	-1.39667
C	16.58473	-5.0393	-1.22527
C	-13.9206	-1.22916	-0.07334
C	-14.8933	-2.19133	0.223802
C	-14.3408	-0.06723	-0.7288
C	-16.217	-2.00829	-0.1239
H	-14.6121	-3.08787	0.768457
C	-15.6628	0.121837	-1.08281
H	-13.6141	0.696258	-0.99397
C	-16.6234	-0.84918	-0.7912
H	-16.9529	-2.76277	0.136023
H	-15.961	1.026605	-1.60486
N	-17.9681	-0.63448	-1.14229
C	-18.5108	0.671808	-0.97562
C	-18.9467	1.405951	-2.11363
C	-18.6005	1.21744	0.280174
C	-19.5177	2.693997	-1.91089
C	-19.1446	2.500825	0.46986
H	-18.251	0.637407	1.130018
C	-19.6062	3.217543	-0.6002
H	-19.2091	2.90995	1.472917
H	-20.0401	4.204236	-0.46178
C	-18.8193	-1.68599	-1.53738
C	-18.3303	-2.75148	-2.29937

C	-20.1773	-1.6539	-1.2166
C	-19.1789	-3.76678	-2.7072
H	-17.2807	-2.77383	-2.57693
C	-21.0213	-2.66858	-1.64335
H	-20.5694	-0.82798	-0.63078
C	-20.5305	-3.73389	-2.38467
H	-18.7806	-4.5856	-3.29884
H	-22.0748	-2.62601	-1.38371
H	-21.1932	-4.528	-2.71235
C	-18.8085	0.919525	-3.43447
C	-19.9618	3.429723	-3.03449
H	-18.3478	-0.05079	-3.59362
H	-20.4039	4.409099	-2.87075
C	-19.8308	2.92632	-4.29955
C	-19.2393	1.66274	-4.50032
H	-20.1733	3.501295	-5.15411
H	-19.1227	1.279876	-5.50932
C	15.81992	-6.24295	-1.21195
N	15.20091	-7.21595	-1.20134
C	18.63801	-6.36084	-1.55386
N	19.16204	-7.38053	-1.68134

Table S12: Cartesian coordinates of **TNPD6**

Atom	X-axis	Y-axis	Z-axis
C	0.591239	-1.62557	0.606787
C	1.964408	-1.6977	0.499497
C	2.684793	-0.512	0.630255
C	2.041312	0.716573	0.86947
C	0.670257	0.783045	1.011207
C	-0.05027	-0.40181	0.878048
H	2.468057	-2.63792	0.287357
H	0.164658	1.727939	1.196114
C	4.094283	-0.22476	0.52229
C	4.343799	1.117199	0.636596
C	5.704772	1.467538	0.477644
C	6.479893	0.341894	0.257156
S	5.520985	-1.13585	0.244659
C	-3.87598	-1.20642	0.919925
C	-3.10584	-2.32251	0.650532
C	-1.72854	-1.99251	0.642272
C	-1.46664	-0.67393	0.900505

S	-2.899	0.240824	1.144603
C	-5.30947	-1.05823	1.032389
C	-6.00593	-0.22309	1.870623
S	-6.41063	-1.90642	-0.02353
C	-7.4003	-0.27694	1.694092
H	-5.51578	0.389723	2.619292
C	-7.80204	-1.1529	0.714455
H	-8.10382	0.290087	2.293851
C	7.90118	0.211234	0.049817
C	8.557473	-0.7496	-0.68819
S	9.047685	1.252141	0.844877
C	9.952076	-0.64349	-0.63523
H	8.032171	-1.50275	-1.26456
C	10.40538	0.398375	0.146365
H	10.62088	-1.31342	-1.16415
C	-0.43114	-2.74503	0.386029
C	3.046545	1.874552	0.877512
C	-9.14917	-1.42337	0.269438
C	-9.69726	-2.54494	-0.31442
S	-10.3553	-0.17561	0.480634
C	-11.0792	-2.38613	-0.57848
C	-11.5996	-1.17464	-0.2122
H	-11.6732	-3.16273	-1.05073
C	11.77	0.799706	0.349317
C	12.30856	1.676172	1.290255
S	12.98951	0.118513	-0.68865
C	13.69363	1.761671	1.161414
C	14.25308	0.988096	0.151812
H	14.31038	2.380384	1.807224
C	-8.96083	-3.80397	-0.64137
H	-8.37754	-3.70823	-1.56479
H	-8.26463	-4.08852	0.153565
H	-9.66388	-4.62859	-0.785
C	11.5445	2.42689	2.330677
H	10.89869	3.194819	1.890517
H	10.90691	1.765323	2.926632
H	12.2329	2.930345	3.013346
C	-3.64391	-3.70131	0.448124
H	-4.62767	-3.82133	0.908308
H	-3.73962	-3.94464	-0.61695
H	-2.9728	-4.44405	0.890368
C	6.215243	2.870841	0.47846
H	6.412644	3.230789	1.495355

H	7.140215	2.965486	-0.0956
H	5.476248	3.544227	0.03429
C	-0.4405	-3.20273	-1.07847
C	-0.94383	-4.44552	-1.4448
C	-0.02829	-2.31595	-2.0764
C	-1.02094	-4.79634	-2.78961
H	-1.26941	-5.14979	-0.6862
C	-0.10332	-2.67318	-3.41743
H	0.35551	-1.34168	-1.79633
C	-0.60386	-3.92495	-3.77632
H	-1.41027	-5.77112	-3.06747
H	-0.64947	-4.18667	-4.82858
C	-0.14791	-3.85429	1.393118
C	0.818009	-4.83134	1.13091
C	-0.76791	-3.84254	2.632431
C	1.131708	-5.76911	2.098187
H	1.313602	-4.86387	0.165582
C	-0.45026	-4.79087	3.606142
H	-1.51494	-3.09283	2.879714
C	0.505796	-5.76553	3.340195
H	1.879666	-6.52748	1.887272
C	2.653035	2.780991	-0.28913
C	1.700583	3.791374	-0.12516
C	3.129968	2.512233	-1.56289
C	1.257939	4.507727	-1.22298
H	1.307766	4.017263	0.861204
C	2.672868	3.228608	-2.66968
H	3.860129	1.725753	-1.73568
C	1.732502	4.239963	-2.50237
H	0.521091	5.293815	-1.08814
H	1.365645	4.814893	-3.34429
C	3.131683	2.574822	2.23422
C	3.609306	3.883553	2.349688
C	2.82912	1.87264	3.391144
C	3.763473	4.457165	3.599501
H	3.851052	4.458387	1.462202
C	2.987792	2.451693	4.650368
H	2.462033	0.850972	3.350226
C	3.456256	3.757027	4.760453
H	4.129511	5.476368	3.679349
O	0.291905	-1.87489	-4.43615
O	3.199052	2.857887	-3.85968
C	0.833521	-0.60978	-4.12136

H	0.103932	0.0254	-3.60353
H	1.734265	-0.69924	-3.50024
C	2.759464	3.533464	-5.0179
H	3.291488	3.085331	-5.8565
H	1.679932	3.409533	-5.16984
C	15.65035	0.992136	-0.06628
C	16.47847	0.361147	-0.95181
H	16.1366	1.655435	0.645442
C	17.3097	-0.97489	-2.71003
C	17.66702	-1.80818	-3.78269
C	19.01936	-1.78706	-3.97351
C	18.37066	-0.34124	-2.10785
C	17.93059	0.500958	-1.01006
C	16.06891	-0.57612	-2.02565
C	18.78148	1.247283	-0.22807
O	14.9443	-0.94206	-2.2941
C	18.37351	2.071939	0.851377
N	18.07157	2.751726	1.736174
C	20.17973	1.229454	-0.46993
N	21.31778	1.210733	-0.67205
H	1.100847	-0.14454	-5.06981
H	2.995536	4.603793	-4.97413
H	0.767555	-6.51552	4.077061
O	-1.12713	-4.67713	4.771347
C	-0.84729	-5.60745	5.794232
H	-1.48789	-5.34301	6.634926
H	-1.07474	-6.63317	5.478594
H	0.201494	-5.55358	6.111352
H	3.58295	4.232127	5.725976
O	2.656839	1.664037	5.698937
C	2.794253	2.196844	6.997763
H	2.154706	3.076422	7.141995
H	2.482127	1.412864	7.686994
H	3.834674	2.470177	7.213017
S	19.86053	-0.76264	-2.85946
C	-12.9724	-0.70122	-0.33376
C	-14.0342	-1.61077	-0.39951
C	-13.2834	0.660819	-0.39217
C	-15.3417	-1.18374	-0.52387
H	-13.833	-2.67488	-0.31583
C	-14.5883	1.095998	-0.52188
H	-12.4853	1.39804	-0.36459
C	-15.6408	0.180082	-0.59427

H	-16.1465	-1.91157	-0.55016
H	-14.8018	2.159401	-0.58057
N	-16.9646	0.640187	-0.70404
C	-17.3385	1.807111	0.022874
C	-17.7222	2.982764	-0.67994
C	-17.3168	1.792309	1.39464
C	-18.1232	4.121957	0.073674
C	-17.6937	2.929413	2.132322
H	-17.01	0.882948	1.904348
C	-18.101	4.064619	1.486409
H	-17.6727	2.892872	3.216698
H	-18.4057	4.943716	2.047936
C	-17.9566	-0.07325	-1.40725
C	-17.643	-0.76429	-2.5816
C	-19.281	-0.05841	-0.96707
C	-18.6295	-1.44094	-3.27911
H	-16.6209	-0.76091	-2.9482
C	-20.2639	-0.72697	-1.68187
H	-19.5374	0.480555	-0.06005
C	-19.9471	-1.4275	-2.83687
H	-18.3665	-1.97183	-4.18915
H	-21.2885	-0.70413	-1.32311
H	-20.7179	-1.95338	-3.39045
C	-17.6922	3.07212	-2.09112
C	-18.5125	5.294719	-0.61471
H	-17.3607	2.213134	-2.66676
H	-18.8247	6.157747	-0.0323
C	-18.489	5.346783	-1.98137
C	-18.0655	4.226654	-2.72493
H	-18.7878	6.253236	-2.49832
H	-18.033	4.28394	-3.80841
H	16.97512	-2.38877	-4.37915
H	19.59672	-2.32083	-4.7164

Table S13: Natural bond orbitals (NBOs) analysis of **TNPR**

**Donor(*i*)**	**Type**	**Acceptor(*j*)**	**Type**	***E*(2) [*kcal/mol*]**	***E*(*j*)-*E*(*i*) [*a.u*]**	***F*(*i,j*) [*a.u*]**
C38-C39	π	C112-C113	π*	31.17	0.31	0.088
C44-C45	π	C109-C110	π*	31.13	0.31	0.088
C109C110	π	C135-C139	π*	26.97	0.3	0.081
C78-C80	π	C74-C76	π*	25.23	0.31	0.079
C41-C42	π	C44-C45	π*	24.78	0.29	0.078
C66-C70	π	C65-C68	π*	24.04	0.29	0.077

C73-C75	π	C78-C80	π*	23.91	0.3	0.077
C112-C113	π	C137-O142	π*	23.72	0.31	0.078
C117-C118	π	C119-C120	π*	22.29	0.3	0.073
C127-C128	π	C125-C126	π*	21.92	0.3	0.073
C119-C120	π	C117-C118	π*	20.85	0.3	0.072
C26-C27	π	C29-C31	π*	19.61	0.3	0.072
C119-C120	π	C135-C139	π*	18.59	0.29	0.066
C74-C76	π	C78-C80	π*	17.57	0.29	0.065
C136-O141	π	C115-C116	π*	4.09	0.44	0.041
C82-C84	π	C4-C5	π*	1.16	0.31	0.017
C16-C17	π	C63-C64	π*	0.79	0.33	0.015
C109-H111	σ	S43-C45	σ*	10.29	0.73	0.077
C140-C149	σ	C149-N150	σ*	8.17	1.61	0.103
C143-N144	σ	C139-C143	σ*	7.99	1.57	0.101
C109-H111	σ	C110-C136	σ*	6.82	0.99	0.074
C11-C12	σ	C12-C26	σ*	6.27	1.25	0.079
C29-C31	σ	C31-C41	σ*	5.98	1.27	0.078
C44-H46	σ	S43-C45	σ*	5.18	0.74	0.055
C76-C80	σ	C78-O154	σ*	4.98	1.09	0.066
C70-H72	σ	C64-C66	σ*	3.98	1.11	0.059
C115-C120	σ	C136-O141	σ*	2.94	1.29	0.055
C92-C94	σ	C94-H98	σ*	1.99	1.12	0.042
C29-H32	σ	C29-C31	σ*	1.69	1.11	0.039
C125C126	σ	C125-H131	σ*	1.66	1.15	0.039
C95-C99	σ	C99-H159	σ*	1.57	1.13	0.038
C103-H105	σ	C68-O101	σ*	0.52	0.91	0.020
C59-H60	σ	C10-C11	σ*	0.50	1.07	0.021
O154	LP(2)	C78-C80	π*	33.27	0.36	0.104
O102	LP(2)	C87-C89	π*	33.13	0.36	0.104
S37	LP(2)	C35-C36	π*	26.52	0.27	0.076
S18	LP(2)	C14-C15	π*	21.11	0.29	0.07
O141	LP(2)	C115-C136	σ*	20.82	0.76	0.114
N148	LP(1)	C140-C147	σ*	12.64	1.04	0.103
S28	LP(1)	C51-H54	σ*	0.51	1.06	0.021

LP = lone pair, (i) donor; (j) acceptor; *E*(2) means energy of hyper conjugative interaction (stabilization energy), Unit in *kcal/mol*; *E*(j) - *E*(i) is the energy difference between donor and acceptor i and j NBO orbitals; *F*(i, j) is the Fock matrix element between i and j NBO orbitals

Table S14: Natural bond orbitals analysis for **TNPD1** with its representative values

**Donor(*i*)**	**Type**	**Acceptor(*j*)**	**Type**	***E*(2) [*kcal/mol*]**	***E*(*j*)-*E*(*i*) [*a.u*]**	***F*(*i,j*) [*a.u*]**
C44-C45	π	C109-C110	π*	30.87	0.31	0.088
C5-C6	π	C3-C4	π*	25.63	0.3	0.079
C63-C64	π	C66-C70	π*	24.63	0.3	0.077
C150-C152	π	C145-C147	π*	23.95	0.3	0.077

C1-C2	π	C3-C4	π*	22.05	0.3	0.075
C112-C115	π	C116-C118	π*	21.56	0.32	0.075
C5-C6	π	C1-C2	π*	20.68	0.3	0.072
C9-C10	π	C3-C4	π*	14.09	0.32	0.063
C38-C39	π	C145-C147	π*	12.03	0.32	0.058
C14-C15	π	C19-C20	π*	10.74	0.31	0.053
C116-C118	π	C109-C110	π*	7.79	0.34	0.046
C117-O119	π	C112-C115	π*	4.86	0.42	0.044
C117-O119	π	C109-C110	π*	3.62	0.43	0.038
C63-C64	π	C16-C17	π*	1.32	0.29	0.018
C73-C75	π	C1-C2	π*	0.84	0.3	0.014
C122-N123	π	C120-N121	π*	0.64	0.47	0.015
C120-N121	π	C122-N123	π*	0.63	0.47	0.015
C109-H111	σ	S43-C45	σ*	10.01	0.73	0.076
C144-C184	σ	C184-N185	σ*	8.88	1.61	0.107
C3-C4	σ	C9-S13	σ*	7.39	0.91	0.073
C14-C15	σ	C16-C33	σ*	6.69	1.14	0.078
C14-C19	σ	C14-C15	σ*	6.55	1.31	0.083
C29-C31	σ	C31-C41	σ*	5.98	1.27	0.078
C27-H30	σ	C26-S28	σ*	5.68	0.74	0.058
C65-C68	σ	C68-C70	σ*	5.29	1.29	0.074
C139-H141	σ	C113-C114	σ*	4.99	1.06	0.065
C55-H56	σ	C15-C16	σ*	4.46	1.07	0.062
C74-H77	σ	C76-C80	σ*	3.99	1.11	0.059
C44-H46	σ	C41-C42	σ*	3.38	1.1	0.055
C51-H53	σ	C42-C44	σ*	2.44	1.08	0.046
C12-C26	σ	C10-C11	σ*	2.05	1.3	0.046
C115-S142	σ	C110-C116	σ*	1.44	1.2	0.037
C97-O133	σ	C95-C99	σ*	1.03	1.52	0.035
C150-H154	σ	C152-N155	σ*	0.93	0.95	0.027
C12-S13	σ	C10-C34	σ*	0.57	1.07	0.022
C4-C34	σ	C82-C84	σ*	0.52	1.21	0.022
C120-N121	σ	C116-C118	σ*	0.5	1.65	0.026
O127	LP(2)	C78-C80	π*	33.18	0.36	0.104
S43	LP(2)	C41-C42	π*	26.06	0.27	0.076
N155	LP(1)	C156-C158	π*	5.27	0.3	0.036
O119	LP(2)	C112-C117	σ*	21.58	0.75	0.116
O102	LP(1)	C87-C89	σ*	7.36	1.14	0.082
S43	LP(1)	C45-C109	σ*	0.55	1.21	0.023

LP = lone pair, (i) donor; (j) acceptor; $E^{(2)}$ means energy of hyper conjugative interaction (stabilization energy), Unit in *kcal/mol*; *E*(j) - *E*(i) is the energy difference between donor and acceptor i and j NBO orbitals; *F*(i, j) is the Fock matrix element between i and j NBO orbitals

Table S15: Natural bond orbitals analysis for **TNPD2** with its representative values

**Donor(*i*)**	**Type**	**Acceptor(*j*)**	**Type**	***E*(2)**	***E*(*j*)-*E*(*i*)**	***F*(*i,j*)**

				**[*kcal/mol*]**	**[*a.u*]**	**[*a.u*]**
C44-C45	π	C109-C110	π*	30.53	0.31	0.087
C113-C139	π	C143-C144	π*	26.26	0.25	0.074
C78-C80	π	C74-C76	π*	25.35	0.31	0.079
C63-C64	π	C66-C70	π*	24.64	0.3	0.077
C152-C154	π	C147-C149	π*	23.93	0.3	0.076
C147-C149	π	C148-C150	π*	21.97	0.3	0.073
C112-C115	π	C117-O119	π*	20.84	0.33	0.075
C3-C4	π	C1-C2	π*	19.89	0.3	0.07
C179-C182	π	C178-C183	π*	18.75	0.31	0.069
C63-C64	π	C65-C68	π*	17.98	0.29	0.066
C11-C12	π	C26-C27	π*	13.22	0.3	0.059
C14-C15	π	C19-C20	π*	10.41	0.31	0.053
C117-O119	π	C112-C115	π*	4.84	0.42	0.044
C117-O119	π	C109-C110	π*	3.63	0.43	0.038
C1-C2	π	C73-C75	π*	0.88	0.31	0.015
C167-C169	π	C158-C160	π*	0.66	0.3	0.013
C120-N121	π	C122-N123	π*	0.63	0.47	0.015
C109-H111	σ	S43-C45	σ*	9.97	0.73	0.076
C118-C120	σ	C120-N121	σ*	8.34	1.62	0.104
C109-H111	σ	C110-C117	σ*	7.19	0.97	0.075
C36-C38	σ	C24-C35	σ*	6.07	1.21	0.077
C41-S43	σ	S28-C31	σ*	5.87	0.87	0.064
C47-H50	σ	C35-C36	σ*	5.41	1.1	0.069
C95-C99	σ	C97-O133	σ*	4.98	1.09	0.066
C59-H61	σ	C10-C11	σ*	4.44	1.07	0.062
C70-H72	σ	C64-C66	σ*	3.98	1.11	0.059
C115-S142	σ	C114-C138	σ*	3.66	1.28	0.061
C112-C115	σ	C117-O119	σ*	2.95	1.33	0.056
C182-C183	σ	C178-H180	σ*	2.84	1.12	0.05
C14-C15	σ	C19-C20	σ*	2.15	1.31	0.048
S43-C45	σ	C109-H111	σ*	1.95	1.07	0.041
N157-C167	σ	C168-C170	σ*	1.38	1.38	0.039
C34-C82	σ	C3-C4	σ*	1.07	1.19	0.032
C1-C2	σ	C16-C33	σ*	0.81	1.14	0.027
C1-C33	σ	C73-C75	σ*	0.58	1.21	0.024
C10-C34	σ	C92-C94	σ*	0.52	1.22	0.023
C120-N121	σ	C116-C118	σ*	0.5	1.65	0.026
O127	LP(2)	C78-C80	π*	33.17	0.36	0.078
S28	LP(2)	C29-C31	π*	23.35	0.28	0.104
N157	LP(1)	C158-C160	π*	5.52	0.3	0.082
O119	LP(2)	C112-C117	σ*	21.51	0.76	0.115
O101	LP(2)	C103-H105	σ*	5.76	0.69	0.058
Cl145	LP(2)	C114-C138	σ*	0.53	0.9	0.02

LP = lone pair, (i) donor; (j) acceptor; *E*(2) means energy of hyper conjugative interaction (stabilization energy), Unit in *kcal/mol*; *E*(j) - *E*(i) is the energy difference between donor and acceptor i and j NBO orbitals; *F*(i, j) is the Fock matrix element between i and j NBO orbitals

Table S16: Natural bond orbitals analysis for **TNPD3** with its representative values

**Donor(*i*)**	**Type**	**Acceptor(*j*)**	**Type**	***E*(2) [*kcal/mol*]**	***E*(*j*)-*E*(*i*) [*a.u*]**	***F*(*i,j*) [*a.u*]**
C44-C45	π	C109-C110	π*	31.36	0.31	0.088
C109-C110	π	C116-C118	π*	26.83	0.29	0.080
C5-C6	π	C3-C 4	π*	25.61	0.30	0.078
C87-C89	π	C83-C85	π*	25.21	0.31	0.079
C41-C42	π	C44-C45	π*	24.70	0.29	0.078
C66-C70	π	C65-C68	π*	23.98	0.3	0.077
C166-C168	π	C165-C167	π*	22.92	0.29	0.075
C146-C148	π	C150-C152	π*	21.99	0.30	0.074
C170-C172	π	C165-C167	π*	20.63	0.29	0.070
C3-C4	π	C1-C2	π*	19.83	0.30	0.070
C113-C114	π	C139-C144	π*	18.75	0.31	0.070
C156-C158	π	C160-C162	π*	17.97	0.32	0.068
C109-C110	π	C44-C45	π*	11.13	0.30	0.054
C116-C118	π	C112-C115	π*	9.36	0.32	0.050
C117-O119	π	C112-C115	π*	4.83	0.42	0.044
C122-N123	π	C120-N121	π*	0.64	0.47	0.015
C65-C68	π	C65-C68	π*	0.62	0.30	0.012
C109-H111	σ	S43-C45	σ*	10.06	0.73	0.077
C118-C120	σ	C120-N121	σ*	8.35	1.62	0.104
C109-H111	σ	C110-C117	σ*	7.18	0.97	0.075
C24-C35	σ	C35-C36	σ*	6.88	1.32	0.085
C31-C41	σ	C41-C42	σ*	6.00	1.31	0.079
C19-C20	σ	C14-C19	σ*	5.96	1.26	0.077
C29-H32	σ	S28-C31	σ*	5.67	0.74	0.058
C41-S43	σ	C42-C51	σ*	5.31	1.11	0.069
C139-H141	σ	C143-C144	σ*	5.28	1.06	0.067
C39-C145	σ	C38-C39	σ*	4.99	1.30	0.072
C55-H56	σ	C15-C16	σ*	4.47	1.07	0.062
C70-H72	σ	C64-C66	σ*	3.98	1.11	0.059
C114-S142	σ	C113-C139	σ*	3.94	1.25	0.063
C181-H183	σ	C177-C180	σ*	3.61	1.14	0.057
C19-C20	σ	C14-C15	σ*	2.99	1.32	0.056
C73-C75	σ	C75-H79	σ*	2.02	1.12	0.043
C65-C68	σ	C70-H72	σ*	1.98	1.14	0.042
C120-N121	σ	C116-C118	σ*	0.50	1.65	0.026
O127	LP(2)	C78-C80	π*	33.20	0.36	0.104
S142	LP(2)	C112-C115	π*	27.02	0.28	0.078
S13	LP(2)	C9-C10	π*	26.52	0.29	0.078
F187	LP(2)	C139-C144	π*	0.51	0.48	0.015

O119	LP(2)	C112-C117	σ*	21.74	0.75	0.116
O101	LP(1)	C65-C68	σ*	7.46	1.15	0.083
F186	LP(2)	C113-C139	σ*	0.50	1.02	0.020

LP = lone pair, (i) donor; (j) acceptor; *E*(2) means energy of hyper conjugative interaction (stabilization energy), Unit in *kcal/mol*; *E*(j) - *E*(i) is the energy difference between donor and acceptor i and j NBO orbitals; *F*(i, j) is the Fock matrix element between i and j NBO orbitals

Table S17: Natural bond orbitals analysis for **TNPD4** with its representative values

**Donor(*i*)**	**Type**	**Acceptor(*j*)**	**Type**	***E*(2) [*kcal/mol*]**	***E*(*j*)-*E*(*i*) [*a.u*]**	***F*(*i,j*) [*a.u*]**
C44-C45	π	C109-C110	π*	31.01	0.31	0.088
C143-C144	π	N184-O186	π*	30.58	0.15	0.065
C109-C110	π	C116-C118	π*	26.48	0.30	0.080
C143-C144	π	C113-C139	π*	25.78	0.31	0.079
C83-C85	π	C82-C84	π*	24.75	0.30	0.078
C165-C167	π	C170-C172	π*	23.80	0.30	0.076
C113-C139	π	C114-C138	π*	22.99	0.28	0.072
C145-C147	π	C150-C152	π*	21.83	0.29	0.071
C5-C6	π	C1-C2	π*	20.68	0.30	0.072
C3-C4	π	C1-C2	π*	19.87	0.30	0.070
C66-C70	π	C63-C64	π*	18.34	0.30	0.068
C156-C158	π	C160-C162	π*	17.98	0.32	0.068
C11-C12	π	C9-C10	π*	16.71	0.32	0.068
C35-C36	π	C38-C 39	π*	15.67	0.31	0.065
C22-C24	π	C35-C36	π*	11.78	0.32	0.058
C92-C94	π	C9-C10	π*	1.37	0.29	0.018
C120-N121	π	C122-N123	π*	0.63	0.47	0.015
C109-H111	σ	S43-C45	σ*	10.01	0.73	0.076
C118-C120	σ	C120-N121	σ*	8.34	1.62	0.104
C3-C4	σ	C9-S13	σ*	7.39	0.91	0.073
C1-C6	σ	C17-S18	σ*	7.36	0.91	0.073
C109-H111	σ	C110-C117	σ*	7.18	0.97	0.075
C11-C12	σ	C10-C34	σ*	6.64	1.15	0.078
C158-H161	σ	C156-C157	σ*	5.84	1.08	0.071
C39-C145	σ	C38-C39	σ*	4.99	1.30	0.072
C139-C144	σ	C113-C139	σ*	3.89	1.31	0.064
C94-C97	σ	O133-C134	σ*	3.64	1.01	0.054
C22-C24	σ	C20-C22	σ*	3.63	1.29	0.061
C181-H183	σ	C177-C180	σ*	3.61	1.14	0.057
C170-C172	σ	C167-C170	σ*	3.47	1.30	0.060
C4-C34	σ	C4-C5	σ*	2.99	1.21	0.054
C148-C152	σ	N155-C156	σ*	2.92	1.12	0.051
C165-C166	σ	N155-C165	σ*	2.01	1.14	0.043
C112-C117	σ	C117-O119	σ*	1.99	1.26	0.045

C120-N121	σ	C116-C118	σ*	0.50	1.65	0.026
O127	LP(2)	C78-C80	π*	33.17	0.36	0.104
O102	LP(2)	C87-C89	π*	32.36	0.36	0.103
N155	LP(1)	C156-C158	π*	5.24	0.30	0.036
O119	LP(2)	C112-C117	σ*	21.69	0.75	0.116
O186	LP(2)	N184-O185	σ*	20.60	0.76	0.113
S43	LP(1)	C45-C109	σ*	0.55	1.21	0.023

LP = lone pair, (i) donor; (j) acceptor; *E*(2) means energy of hyper conjugative interaction (stabilization energy), Unit in *kcal/mol*; *E*(j) - *E*(i) is the energy difference between donor and acceptor i and j NBO orbitals; *F*(i, j) is the Fock matrix element between i and j NBO orbitals

Table S18: Natural bond orbitals analysis for **TNPD5** with its representative values

**Donor(*i*)**	**Type**	**Acceptor(*j*)**	**Type**	***E*(2) [*kcal/mol*]**	***E*(*j*)-*E*(*i*) [*a.u*]**	***F*(*i,j*) [*a.u*]**
C44-C45	π	C109-C110	π*	32.04	0.31	0.089
C109-C110	π	C116-C118	π*	27.30	0.29	0.081
C5-C6	π	C3-C4	π*	25.70	0.30	0.079
C83-C85	π	C82-C84	π*	24.71	0.30	0.078
C150-C152	π	C145-C147	π*	23.96	0.30	0.077
C113-C114	π	C138-C143	π*	22.77	0.30	0.075
C145-C147	π	C146-C148	π*	21.98	0.30	0.073
C170-C172	π	C165-C167	π*	20.56	0.29	0.070
C3-C4	π	C1-C2	π*	19.88	0.30	0.070
C177-C180	π	C176-C181	π*	18.75	0.31	0.069
C156-C158	π	C160-C162	π*	17.99	0.32	0.068
C44-C45	π	C41-C42	π*	17.05	0.30	0.065
C29-C31	π	C26-C27	π*	16.97	0.31	0.067
C41-C42	π	C29-C31	π*	15.34	0.30	0.061
C35-C36	π	C22-C24	π*	14.45	0.31	0.062
C117-O119	π	C112-C115	π*	4.84	0.41	0.043
C117-O119	π	C109-C110	π*	3.58	0.43	0.038
C122-N123	π	C120-N121	π*	0.63	0.47	0.015
C109-H111	σ	S43-C45	σ*	10.08	0.73	0.077
C143-C186	σ	C186-N187	σ*	8.96	1.62	0.108
C118-C122	σ	C122-N123	σ*	8.13	1.61	0.103
C3-C4	σ	C9-S13	σ*	7.40	0.91	0.073
C118-C120	σ	C116-C118	σ*	6.21	1.34	0.081
C19-C20	σ	C14-C19	σ*	5.99	1.26	0.078
C39-C145	σ	C38-C39	σ*	4.98	1.30	0.072
C10-C11	σ	C9-C10	σ*	4.89	1.27	0.071
C180-H182	σ	C159-C177	σ*	4.64	1.08	0.063
C15-C55	σ	C14-S18	σ*	3.99	0.85	0.052
C143-C144	σ	C184-N185	σ*	3.76	1.60	0.070
C165-C166	σ	N155-C156	σ*	3.16	1.12	0.053
C180-C181	σ	C177-H179	σ*	2.88	1.11	0.051
C82-C84	σ	C83-H86	σ*	2.64	1.13	0.049

C166-C168	σ	C172-H175	σ*	2.40	1.14	0.047
C2-C3	σ	C4-C34	σ*	2.37	1.11	0.046
C78-O127	σ	C78-C80	σ*	1.21	1.51	0.038
C120-N121	σ	C116-C118	σ*	0.50	1.64	0.026
O127	LP(2)	C78-C80	π*	33.18	0.36	0.104
S142	LP(2)	C112-C115	π*	26.91	0.28	0.078
N155	LP(1)	C156-C158	π*	5.38	0.30	0.036
O119	LP(2)	C112-C117	σ*	21.74	0.75	0.116
O119	LP(2)	C112-C117	σ*	21.85	0.75	0.116
N121	LP(1)	C118-C120	σ*	12.64	1.04	0.103
S43	LP(1)	C45-C109	σ*	0.55	1.21	0.023

LP = lone pair, (i) donor; (j) acceptor; *E*(2) means energy of hyper conjugative interaction (stabilization energy), Unit in *kcal/mol*; *E*(j) - *E*(i) is the energy difference between donor and acceptor i and j NBO orbitals; *F*(i, j) is the Fock matrix element between i and j NBO orbitals

Table S19: Natural bond orbitals analysis for **TNPD6** with its representative values

**Donor(*i*)**	**Type**	**Acceptor(*j*)**	**Type**	***E*(2) [*kcal/mol*]**	***E*(*j*)-*E*(*i*) [*a.u*]**	***F*(*i,j*) [*a.u*]**
C44-C45	π	C109–C110	π*	29.78	0.31	0.087
C5-C6	π	C3-C4	π*	25.53	0.30	0.078
C83-C85	π	C82-C84	π*	24.59	0.30	0.078
C66-C70	π	C65-C68	π*	23.99	0.30	0.077
C160-C162	π	C159-C161	π*	22.92	0.29	0.075
C140-C142	π	C144-C146	π*	21.98	0.30	0.074
C5-C6	π	C1-C2	π*	20.81	0.30	0.072
C112-C115	π	C117-O119	π*	19.88	0.32	0.073
C171-C174	π	C170-C 75	π*	18.75	0.31	0.069
C150-C152	π	C154-C156	π*	17.96	0.32	0.068
C29-C31	π	C26-C27	π*	16.96	0.31	0.067
C35-C36	π	C38-C39	π*	15.68	0.31	0.065
C5-C6	π	C16-C17	π*	14.55	0.30	0.059
C117-O119	π	C112-C115	π*	4.65	0.42	0.044
C1-C2	π	C73-C75	π*	1.06	0.31	0.016
C122-N123	π	C120-N121	π*	0.62	0.47	0.015
C109-H111	σ	S43-C45	σ*	9.94	0.73	0.076
C118-C120	σ	C120-N	σ*	8.33	1.62	0.104
C3-C4	σ	C9-S13	σ*	7.4	0.90	0.073
C24-C35	σ	C35-C36	σ*	6.87	1.32	0.085
C19-C20	σ	C14-C9	σ*	5.97	1.26	0.077
C44-H46	σ	S43-C45	σ*	5.23	0.74	0.056
C39-C139	σ	C38-C39	σ*	4.99	1.30	0.072
C166-H169	σ	C161-C164	σ*	3.99	1.11	0.060
C44-H46	σ	C41-C42	σ*	3.33	1.11	0.054
C35-C36	σ	C38-H40	σ*	2.99	1.14	0.052
C1-C33	σ	C16-C33	σ*	1.99	1.03	0.040

C146-N149	σ	C159-C161	σ*	1.90	1.37	0.046
C36-C38	σ	C47-H49	σ*	0.61	1.07	0.023
C120-N121	σ	C116-C118	σ*	0.50	1.64	0.026
O127	LP(2)	C78-C80	π*	33.19	0.36	0.104
S138	LP(2)	C112-C115	π*	27.31	0.28	0.078
N149	LP(1)	C159-C161	π*	25.84	0.30	0.081
N149	LP(1)	C 150-C152	π*	5.07	0.30	0.035
O119	LP(2)	C112-C117	σ*	21.56	0.76	0.116
N123	LP(1)	C118-C122	σ*	12.59	1.04	0.102
S43	LP(1)	C45-C109	σ*	0.55	1.21	0.023

LP = lone pair, (i) donor; (j) acceptor; *E*(2) means energy of hyper conjugative interaction (stabilization energy), Unit in *kcal/mol*; *E*(j) - *E*(i) is the energy difference between donor and acceptor i and j NBO orbitals; *F*(i, j) is the Fock matrix element between i and j NBO orbitals

Table S20: Calculated energies (*E*) and energy gap (Δ*E*) of HOMO-1, LUMO+1, HOMO-2 and LUMO+2 for **TNPR**, **TNPD1-TNPD6**.

**Compounds**	E_{HOMO-1}	E_{LUMO+1}	**Δ*E***	E_{HOMO-2}	E_{LUMO+2}	**Δ*E***
**TNPR**	-5.988	-3.26	2.728	-6.483	-2.611	3.872
**TNPD1**	-5.621	-2.815	2.806	-6.004	-2.047	3.957
**TNPD2**	-5.621	-2.787	2.834	-6.003	-2.043	3.96
**TNPD3**	-5.634	-3.501	2.133	-6.02	-2.05	3.97
**TNPD4**	-5.627	-2.870	2.757	-6.004	-2.591	3.413
**TNPD5**	-5.639	-2.92	2.719	-6.03	-2.273	3.757
**TNPD6**	-5.616	-2.645	2.971	-5.997	-2.016	3.981

Table S21: Wavelength, excitation energy and oscillator strength of **TNPR** in gas phase

**NO**	λ_{DFT}	***E* (*eV*)**	f_{os}	**MO contributions**
1	671.128	1.847	2.637	H→L (86%), H-1→L+1 (5%), H→L+1 (5%)
2	621.693	1.994	0.029	H→L+1 (91%), H-1→L (2%), H→L (5%)
3	518.090	2.393	0.217	H-1→L (89%), H→L (3%)
4	507.466	2.443	0.949	H-1→L+1 (73%), H→L (5%), H→L+1 (2%), H→L+2 (6%), H→L+4 (4%)
5	485.831	2.552	0.077	H-1→L+1 (10%), H→L+2 (70%), H-1→L+3 (8%), H→L+3 (5%)
6	478.205	2.593	0.002	H-1→L+2 (10%), H→L+3 (79%), H→L+2 (6%)

MO=molecular orbital, H=HOMO, L=LUMO, f_{os}= oscillator strength, wavelength= λ (*nm*)

Table S22: Wavelength, excitation energy and oscillator strength of investigated compound (**TNPD1**) in gas phase

**NO**	**DFT λ (*nm*)**	***E*(*eV*)**	f_{OS}	**MO contributions**
1	807.241	1.536	0.387	H→L (93%), H-1→L (5%)
2	666.045	1.862	0.430	H-1→L (82%), H-2→L (9%), H→L (7%)
3	601.165	2.062	0.658	H-1→L+1 (11%), H→L+1 (83%), H-2→L+1 (2%)

4	561.243	2.209	0.239	H-2→L (74%), H-1→L (12%), H-4→L (7%)
5	518.459	2.391	0.560	H-2→L+1 (12%), H-1→L+1 (66%), H→L+1 (16%), H→L+2 (2%)
6	479.537	2.586	0.446	H-4→L (27%), H-3→L (36%), H-2→L (13%), H→L+2 (13%), H-9→L (5%)

MO=molecular orbital, H=HOMO, L=LUMO, f_{os}= oscillator strength, wavelength= λ (*nm*)

Table S23: Wavelength, excitation energy and oscillator strength of investigated compound (**TNPD2**) in gas phase

NO	DFT λ (*nm*)	$E(eV)$	f_{OS}	MO contributions
1	783.668	1.582	0.407	H→L (92%), H-1→L (6%)
2	652.034	1.902	0.464	H-1→L (80%), H-2→L (9%), H→L (8%)
3	587.854	2.109	0.699	H-1→L+1 (12%), H→L+1 (82%), H-2→L+1 (2%)
4	550.527	2.252	0.235	H-2→L (71%), H-1→L (13%), H-4→L (7%), H-3→L (4%)
5	509.468	2.434	0.596	H-2→L+1 (12%), H-1→L+1 (63%), H→L+1 (17%), H→L+2 (3%)
6	474.908	2.611	0.842	H-4→L (17%), H-3→L (22%), H-2→L (14%), H→L+2 (33%), H-9→L (4%), H→L+3 (2%)

MO=molecular orbital, H=HOMO, L=LUMO, f_{os}= oscillator strength, wavelength= λ (*nm*)

Table S24: Wavelength, excitation energy and oscillator strength of investigated compound (**TNPD3**) in gas phase

NO	DFT λ (*nm*)	$E(eV)$	f_{OS}	MO contributions
1	843.602	1.470	0.339	H→L (94%), H-1→L (5%)
2	687.693	1.803	0.384	H-1→L (84%), H-2→L (8%), H→L (6%)
3	608.900	2.036	0.626	H-1→L+1 (10%), H→L+1 (85%), H-2→L+1 (2%)
4	577.772	2.146	0.266	H-2→L (77%), H-1→L (10%), H-4→L (7%)
5	522.831	2.371	0.514	H-2→L+1 (12%), H-1→L+1 (68%), H→L+1 (14%),
6	488.974	2.536	0.281	H-4→L (43%), H-3→L (28%), H-2→L (12%), H-9→L (6%), H→L+2 (4%)

MO=molecular orbital, H=HOMO, L=LUMO, f_{os}= oscillator strength, wavelength= λ (*nm*)

Table S25: Wavelength, excitation energy and oscillator strength of investigated compound (**TNPD4**) in gas phase

NO	λ_{DFT}	E (eV)	f_{os}	MO contributions
1	816.814	1.518	0.369	H→L (94%), H-1→L (5%)
2	668.523	1.855	0.407	H-1→L (82%), H-2→L (9%), H→L (6%)
3	610.309	2.032	0.594	H→L+1 (85%), H-2→L+1 (2%), H-1→L+1 (9%)
4	566.837	2.187	0.247	H-2→L (74%), H-1→L (13%), H-4→L (7%)
5	523.914	2.367	0.608	H-2→L+1 (12%), H-1→L+1 (63%), H→L+1 (14%), H→L+2 (4%), H→L+3 (3%)
6	501.981	2.470	0.041	H→L+2 (89%), H-1→L+1 (6%), H-1→L+2 (3%)

MO=molecular orbital, H=HOMO, L=LUMO, f_{os}= oscillator strength, wavelength= λ (*nm*)

Table S26: Wavelength, excitation energy and oscillator strength of investigated compound (**TNPD5**) in gas phase

NO	DFT λ (*nm*)	*E*(*eV*)	f_{OS}	MO contributions
1	931.302	1.331	0.264	H→L (96%), H-1→L (3%)
2	742.376	1.670	0.377	H-1→L (88%), H-2→L (6%), H→L (4%)
3	642.372	1.930	0.563	H→L+1 (88%), H-1→L+1 (8%)
4	610.459	2.031	0.246	H-2→L (80%), H-4→L (7%), H-1→L (8%)
5	548.821	2.259	0.564	H-2→L+1 (10%), H-1→L+1 (73%), H→L+1 (11%),
6	517.982	2.394	0.120	H-4→L (18%), H-3→L (67%), H-9→L (2%), H-2→L (8%)

MO=molecular orbital, H=HOMO, L=LUMO, f_{os}= oscillator strength, wavelength= λ (*nm*)

Table S27: Wavelength, excitation energy and oscillator strength of investigated compound (**TNPD6**) in gas phase

NO	DFT λ (*nm*)	*E*(*eV*)	f_{OS}	MO contributions
1	697.442	1.778	0.653	H→L (90%), H-1→L (7%)
2	588.272	2.108	0.600	H-1→L (77%), H→L (10%), H-2→L (9%)
3	542.316	2.286	0.607	H-1→L+1 (12%), H→L+1 (78%), H-2→L+1 (3%), H→L+2 (3%)
4	506.017	2.450	0.410	H-2→L (72%), H-1→L (13%), H-4→L (4%), H-3→L (5%), H→L+2 (2%)
5	474.199	2.615	0.618	H-2→L+1 (11%), H-1→L+1 (51%), H→L+1 (21%), H→L+2 (11%),
6	463.111	2.677	0.910	H-1→L+1 (13%), H→L+2 (67%), H-2→L (6%), H-2→L+1 (2%), H-1→L+3 (3%), H→L+3 (5%)

MO=molecular orbital, H=HOMO, L=LUMO, f_{os}= oscillator strength, wavelength= λ (*nm*)

Table S28: Wavelength, excitation energy and oscillator strength of reference compound (**TNPR**) in chloroform

NO	DFT λ (*nm*)	*E* (*eV*)	f_{os}	MO contributions
1	706.785	1.754	2.960	H→L (86%), H-1→L+1 (8%)
2	658.895	1.882	0.016	H→L+1 (89%), H-1→L (7%)
3	538.757	2.301	0.064	H-1→L (83%), H-2→L+1 (5%), H→L+1 (7%)
4	525.513	2.359	0.921	H-1→L+1 (75%), H→L (10%), H-2→L (5%), H→L+2 (3%), H→L+4 (2%)
5	502.530	2.467	0.166	H-1→L+3 (10%), H→L+2 (77%), H-1→L+1 (6%)
6	493.214	2.514	0.002	H-1→L+2 (12%), H→L+3 (82%),

MO=molecular orbital, H=HOMO, L=LUMO, f_{os}= oscillator strength, wavelength= λ (*nm*)

Table S29: Wavelength, excitation energy and oscillator strength of investigated compound (**TNPD1**) in chloroform

NO	DFT λ (*nm*)	*E*(*eV*)	f_{os}	MO contributions
1	774.127	1.602	0.704	H-1→L (13%), H→L (83%), H-2→L (3%)
2	652.720	1.900	0.382	H-2→L (11%), H-1→L (68%), H→L (17%),

3	583.537	2.125	0.969	H-1→L+1 (19%), H→L+1 (70%), H-2→L+1 (5%), H→L+2 (3%)
4	550.112	2.254	0.165	H-2→L (68%), H-1→L (18%), H-3→L (9%)
5	503.285	2.464	0.242	H-2→L+1 (12%), H-1→L+1 (55%), H→L+1 (27%),
6	478.168	2.593	1.561	H→L+2 (79%), H-1→L+4 (2%), H→L+3 (4%), H→L+4 (2%)

MO=molecular orbital, H=HOMO, L=LUMO, f_{os}= oscillator strength, wavelength= λ (*nm*)

Table S30: Wavelength, excitation energy and oscillator strength of investigated compound (**TNPD2**) in chloroform

NO	DFT λ (*nm*)	*E*(*eV*)	f_{os}	MO contributions
1	766.754	1.617	0.690	H-1→L (13%), H→L (82%), H-2→L (3%)
2	649.574	1.909	0.395	H-2→L (11%), H-1→L (67%), H→L (18%),
3	576.939	2.149	1.007	H-1→L+1 (20%), H→L+1 (68%), H-2→L+1 (4%), H→L+2 (3%)
4	546.836	2.267	0.170	H-3→L (11%), H-2→L (66%), H-1→L (18%),
5	499.332	2.483	0.243	H-2→L+1 (12%), H-1→L+1 (54%), H→L+1 (28%),
6	479.018	2.588	1.572	H→L+2 (80%), H-1→L+3 (3%), H→L+3 (6%)

MO=molecular orbital, H=HOMO, L=LUMO, f_{os}= oscillator strength, wavelength= λ (*nm*)

Table S31: Wavelength, excitation energy and oscillator strength of investigated compound (**TNPD3**) in chloroform

NO	DFT λ (*nm*)	*E*(*eV*)	f_{os}	MO contributions
1	799.279	1.551	0.627	H-1→L (11%), H→L (85%), H-2→L (2%)
2	667.910	1.856	0.386	H-2→L (11%), H-1→L (71%), H→L (14%),
3	585.632	2.117	0.953	H-1→L+1 (17%), H→L+1 (72%), H-2→L+1 (5%), H→L+2 (2%)
4	564.463	2.197	0.172	H-2→L (70%), H-1→L (17%), H-3→L (9%)
5	503.755	2.461	0.263	H-2→L+1 (13%), H-1→L+1 (56%), H→L+1 (26%),
6	478.039	2.594	1.035	H-3→L (33%), H-2→L (11%), H→L+2 (39%), H-9→L (4%), H-4→L (3%), H→L+3 (2%)

MO=molecular orbital, H=HOMO, L=LUMO, f_{os}= oscillator strength, wavelength= λ (*nm*)

Table S32: Wavelength, excitation energy and oscillator strength of investigated compound (**TNPD4**) in chloroform

NO	DFT λ (*nm*)	*E* (*eV*)	f_{os}	MO contributions
1	780.315	1.589	0.665	H-1→L (11%), H→L (84%), H-2→L (3%)
2	654.236	1.895	0.384	H-2→L (12%), H-1→L (68%), H→L (16%),
3	592.121	2.094	0.868	H-1→L+1 (16%), H→L+1 (72%), H-2→L+1 (4%), H→L+3 (2%)
4	555.186	2.233	0.161	H-2→L (68%), H-1→L (19%), H-3→L (8%)
5	512.925	2.417	0.430	H-2→L+1 (10%), H-1→L+1 (39%), H→L+1 (26%), H→L+2 (15%), H-1→L+2 (4%)
6	498.008	2.490	0.077	H-1→L+1 (20%), H→L+2 (67%), H-2→L+1 (3%), H-1→L+2 (2%), H→L+3 (5%)

Table S33: Wavelength, excitation energy and oscillator strength of investigated compound (**TNPD5**) in chloroform

NO	DFT λ (*nm*)	*E*(*eV*)	f_{os}	MO contributions
1	852.242	1.455	0.553	H-1→L (10%), H→L (87%),
2	709.413	1.748	0.408	H-1→L (75%), H→L (13%), H-2→L (9%)
3	604.241	2.052	0.966	H-1→L+1 (18%), H→L+1 (73%), H-2→L+1 (4%)
4	586.796	2.113	0.144	H-2→L (73%), H-1→L (14%), H-4→L (2%), H-3→L (8%)
5	521.402	2.378	0.298	H-2→L+1 (11%), H-1→L+1 (58%), H→L+1 (25%),
6	495.145	2.504	0.178	H-3→L (61%), H-2→L (15%), H-9→L (6%), H-4→L (9%)

MO=molecular orbital, H=HOMO, L=LUMO, f_{os}= oscillator strength, wavelength= λ (*nm*)

Table S34: Wavelength, excitation energy and oscillator strength of investigated compound (**TNPD6**) in chloroform

NO	DFT λ (*nm*)	*E*(*eV*)	f_{os}	MO contributions
1	694.745	1.785	0.997	H-1→L (14%), H→L (79%), H-2→L (3%)
2	590.204	2.101	0.482	H-2→L (11%), H-1→L (64%), H→L (20%),
3	540.802	2.293	0.786	H-1→L+1 (18%), H→L+1 (67%), H-2→L+1 (5%), H→L+2 (4%)
4	506.948	2.446	0.260	H-3→L (10%), H-2→L (67%), H-1→L (19%),
5	472.645	2.623	1.075	H→L+2 (73%), H-2→L+1 (3%), H-1→L+1 (9%), H-1→L+3 (3%), H→L+3 (5%)
6	470.368	2.636	0.414	H-2→L+1 (11%), H-1→L+1 (45%), H→L+1 (31%), H→L+2 (7%)

MO=molecular orbital, H=HOMO, L=LUMO, f_{os}= oscillator strength, wavelength= λ (*nm*)

Table S35: Percentages of donor, π-spacer and acceptor for HOMOs and LUMOs of **TNPR** and **TNPD1-TNPD6**

Compounds	HOMO			LUMO		
	Acceptor	Donor	π-spacer	Acceptor	Donor	π-spacer
**TNPR**	7.6	-----	92.4	62.6	------	37.4
**TNPD1**	1.1	20.8	78.1	82.0	0.0	18.0
**TNPD2**	1.0	21.3	77.7	82.5	0.0	17.5
**TNPD3**	1.0	20.3	78.7	82.9	0.0	17.1
**TNPD4**	1.1	19.5	79.4	82.7	0.0	17.3
**TNPD5**	1.0	22.7	76.3	84.0	0.0	16.0
**TNPD6**	1.0	19.4	79.6	75.4	0.0	24.6

Table S36: Dipole polarizability (*D*) of the studied compounds (**TNPR** and **TNPD1-TNPD6**).

Dipole Moment	TNPR	TNPD1	TNPD2	TNPD3	TNPD4	TNPD5	TNPD6
μ_x	0.8570	-7.9798	10.3447	11.3761	-8.2252	-11.7648	-9.4745
μ_y	2.3446	4.0619	-1.9208	-4.9929	4.0918	9.6547	-0.5668

μ_z	0.6720	1.2212	0.7554	0.4143	1.0095	1.5017	-0.4329
μ_{total}	2.5852	9.0370	10.5486	12.4305	9.2421	15.2931	9.5013
Polarizability							
α_{xx}	6.525×10^{-22}	5.628×10^{-22}	$5.638x10^{-22}$	5.672×10^{-22}	5.653×10^{-22}	5.898×10^{-22}	5.281×10^{-22}
α_{yy}	2.464×10^{-22}	2.897×10^{-22}	$2.967x10^{-22}$	2.811×10^{-22}	2.873×10^{-22}	2.998×10^{-22}	2.294×10^{-22}
α_{zz}	1.605×10^{-22}	1.948×10^{-22}	$5.859x10^{-22}$	1.998×10^{-22}	1.930×10^{-22}	1.942×10^{-22}	2.179×10^{-22}
α_{total}	3.532×10^{-22}	3.491×10^{-22}	$3.510x10^{-22}$	3.494×10^{-22}	3.485×10^{-22}	3.613×10^{-22}	3.251×10^{-22}
2nd Hyper pol.							
γ_X	7.977×10^{-32}	6.057×10^{-32}	5.725×10^{-32}	6.831×10^{-32}	6.216×10^{-32}	9.307×10^{-32}	4.099×10^{-32}
γ_Y	1.620×10^{-34}	4.220×10^{-34}	6.275×10^{-34}	6.512×10^{-34}	4.562×10^{-34}	8.709×10^{-34}	2.990×10^{-34}
γ_Z	2.055×10^{-34}	6.896×10^{-34}	4.780×10^{-34}	4.799×10^{-34}	5.030×10^{-34}	7.766×10^{-34}	2.675×10^{-34}
Average<γ>	8.014×10^{-32}	6.168×10^{-32}	5.835×10^{-32}	6.944×10^{-32}	6.312×10^{-32}	9.472×10^{-32}	4.157×10^{-32}
Magnitude of γ	7.977×10^{-32}	6.058×10^{-32}	5.725×10^{-32}	6.831×10^{-32}	6.216×10^{-32}	9.308×10^{-32}	4.100×10^{-32}

Table S37: Frequency dependent second hyperpolarizability (*esu*) of studied compounds (**TNPR** and **TNPD1-TNPD6**).

**Parameters**	**Frequency** ω	**TNPR**	**TNPD1**	**TNPD2**	**TNPD3**
$\gamma(-\omega,\omega,0,0)$	0.000	8.014×10^{-32}	6.168×10^{-32}	$5.835x10^{-32}$	6.944×10^{-32}
	1907.21*nm*	1.084×10^{-31}	9.584×10^{-32}	$8.970x10^{-32}$	1.148×10^{-31}
$\gamma(-2\omega,\omega,\omega,0)$	0.000	8.014×10^{-32}	6.168×10^{-32}	$5.835x10^{-32}$	6.944×10^{-32}
	1907.21*nm*	3.716×10^{-31}	-1.689×10^{-28}	$5.788x10^{-30}$	-1.112×10^{-30}

Continued…

**TNPD4**	**TNPD5**	**TNPD6**
6.312×10^{-32}	9.471×10^{-32}	4.156×10^{-32}
9.937×10^{-32}	1.804×10^{-31}	5.673×10^{-32}
6.312×10^{-32}	9.472×10^{-32}	4.156×10^{-32}
-4.438×10^{-30}	-4.235×10^{-31}	2.270×10^{-31}

Table S38: The computed first hyperpolarizability (β_{tot}) and major contributing tensors (*esu*) of **TNPR** and **TNPD1-TNPD6**.

**Polarizability**	**TNPR**	**TNPD1**	**TNPD2**	**TNPD3**
$\boldsymbol{\beta_{xxx}}$	-2.968×10^{-30}	-3.421×10^{-27}	$3.243x10^{-27}$	3.719×10^{-27}
$\boldsymbol{\beta_{xxy}}$	-8.803×10^{-29}	-5.478×10^{-30}	$4.765x10^{-29}$	1.963×10^{-28}
$\boldsymbol{\beta_{xyy}}$	4.350×10^{-30}	5.916×10^{-29}	$-7.975x10^{-29}$	-3.971×10^{-29}
$\boldsymbol{\beta_{yyy}}$	3.627×10^{-30}	-5.220×10^{-29}	$1.025x10^{-28}$	2.843×10^{-29}

β_{xxz}	2.756×10^{-28}	3.248×10^{-28}	2.434x10^{-28}	2.405×10^{-28}
β_{yyz}	-6.078×10^{-30}	-1.173×10^{-29}	-9.759x10^{-30}	-6.791×10^{-31}
β_{xzz}	2.119×10^{-29}	-4.306×10^{-29}	2.700x10^{-29}	2.275×10^{-29}
β_{yzz}	-3.105×10^{-30}	1.686×10^{-30}	-2.885x10^{-30}	2.436×10^{-30}
β_{zzz}	1.552×10^{-30}	3.578×10^{-30}	4.897x10^{-31}	-5.767×10^{-32}
β_{total}	2.858×10^{-28}	3.420×10^{-27}	3.202x10^{-27}	3.717×10^{-27}

Continued…

TNPD4	TNPD5	TNPD6
-3.478×10^{-27}	-4.620×10^{-27}	-2.526×10^{-27}
-5.501×10^{-29}	1.363×10^{-29}	-9.989×10^{-29}
5.923×10^{-29}	2.945×10^{-29}	1.188×10^{-29}
-3.211×10^{-29}	-2.362×10^{-29}	-9.186×10^{-30}
2.544×10^{-28}	3.968×10^{-28}	1.864×10^{-28}
-7.260×10^{-30}	-2.302×10^{-30}	-9.600×10^{-30}
-2.575×10^{-29}	-4.512×10^{-29}	1.134×10^{-30}
5.744×10^{-32}	4.233×10^{-30}	-1.066×10^{-29}
1.650×10^{-30}	2.695×10^{-30}	-1.104×10^{-29}
3.455×10^{-27}	4.653×10^{-27}	2.522×10^{-27}

Table S39: Frequency dependent first hyperpolarizability (*esu*) of studied compounds (**TNPR** and **TNPD1-TNPD6**).

	Parameters	Frequency ω	TNPR	TNPD1	TNPD2	TNPD3
Static	β $(-\omega;\omega,0,)$	0.000	2.858×10^{-28}	3.420×10^{-27}	3.202x10^{-27}	3.717×10^{-27}
	β $(-2, \omega;\omega,\omega)$	0.000	2.858×10^{-28}	3.420×10^{-27}	3.202x10^{-27}	3.717×10^{-27}
Specific	β $(-\omega;\omega,0)$	1907.21*nm*	3.248×10^{-28}	4.315×10^{-27}	4.039x10^{-27}	4.786×10^{-27}
	β $(-2\omega;\omega,\omega)$	1907.21*nm*	6.696×10^{-28}	1.189×10^{-26}	1.079 x10^{-26}	1.492×10^{-26}

Continued…

TNPD4	TNPD5	TNPD6
3.455×10^{-27}	4.653×10^{-27}	2.522×10^{-27}
3.455×10^{-27}	4.653×10^{-27}	2.522×10^{-27}
4.382×10^{-27}	6.233×10^{-27}	3.024×10^{-27}
1.238×10^{-26}	2.776×10^{-26}	6.337×10^{-27}

Table S40: IUPAC names of reference and derivative compounds.

**TNPR**	2,2'-((2*Z*,2'*Z*)-5',5'''-4(4,4,9,9-tetrakis(3-isobutoxyphenyl)-3,8-dimethyl-4,9-dihydro-*s*-indaceno[1,2-*b*:5,6-*b*']dithiophene-2,7-diyl)bis(3-methyl-[2,2'-bithiophene]-5',5-diyl))bis(methanylylidene))bis(3-oxo-2,3-dihydro-1*H*-indene-2,1-diylidene))dimalononitrile
**TNPD1**	(*Z*)-2-(7-cyano-2-((3-methyl-5'-(4,4,9,9-tetrakis(3-isobutoxyphenyl)-3,8-dimethyl-7-(3'-methyl-5'-(4-naphthalen-1-yl(phenyl)amino)phenyl)-[2,2'-bithiophene]-5-yl)-4,9-dihydro-*s*-indaceno[1,2-*b*:5,6-*b*']dithiophene-2-yl)-[2,2'-bithiophene]-5-yl] methylene)-1-oxo-1*H*-benzo[*b*]cyclopenta[*d*]thiophene-3(2*H*)-ylidene)malonitrile
**TNPD2**	(*Z*)-2-(6,7-dichloro-2-((3-methyl-5'-(4,4,9,9-tetrakis(3-isobutoxyphenyl)-3,8-dimethyl-7-(3'-methyl-5'-(4-naphthalen-1-yl(phenyl)amino)phenyl)-[2,2'-bithiophene]-5-yl)-4,9-dihydro-*s*-indaceno[1,2-*b*:5,6-*b*']dithiophene-2-yl)-[2,2'-bithiophene]-5-yl] methylene)-1-oxo-1*H*-benzo[*b*]cyclopenta[*d*]thiophen-3(2*H*)-ylidene)malonitrile
**TNPD3**	(*Z*)-2-(2-((3-methyl-5'-(4,4,9,9-tetrakis(3-isobutoxyphenyl)-3,8-dimethyl-7-(3'-methyl-5'-(4-naphthalen-1-yl(phenyl)amino)phenyl)-[2,2'-bithiophene]-5-yl)-4,9-dihydro-*s*-indaceno[1,2-*b*:5,6-*b*']dithiophene-2-yl) -[2,2'-bithiophene]-5-yl] methylene)-1-oxo-6,7-bis(trifluoromethyl)-1*H*-benzo[*b*]cyclopenta[*d*]thiophen-3(2*H*)-ylidene)malonitrile
**TNPD4**	(*Z*)-2-(2-((3-methyl-5'-(4,4,9,9-tetrakis(3-isobutoxyphenyl)-3,8-dimethyl-7-(3'-methyl-5'-(4-naphthalen-1-yl(phenyl)amino)phenyl)-[2,2'-bithiophene]-5-yl)-4,9-dihydro-*s*-indaceno[1,2-*b*:5,6-*b*']dithiophene-2-yl)-[2,2'-bithiophen]-5-yl] methylene)-7-nitro-1-oxo-1*H*-benzo[*b*]cyclopenta[*d*]thiophen-3(2*H*)-ylidene)malonitrile
**TNPD5**	(*Z*)-1-(dicyanomethylene)-2-((3-methyl-5'-(4,4,9,9-tetrakis(3-isobutoxyphenyl)-3,8-dimethyl-7-(3'-methyl-5'-(4-naphthalen-1-yl(phenyl)amino)phenyl)-[2,2'-bithiophene]-5-yl)-4,9-dihydro-*s*-indaceno[1,2-*b*:5,6-*b*']dithiophene-2-yl)-[2,2'-bithiophen]-5-yl] methylene)-3-oxo-2,3-dihydro-1*H*-benzo[*b*]cyclopenta[*d*]thiophene-6,7-dicarbonitrile
**TNPD6**	(*Z*)-2-(253-methyl-5'-(4,4,9,9-tetrakis(3-isobutoxyphenyl)-3,8-dimethyl-7-(3'-methyl-5'-(4-naphthalen-1-yl(phenyl)amino)phenyl)-[2,2'-bithiophen]-5-yl)-4,9-dihydro-*s*-indaceno[1,2-*b*:5,6-*b*']dithiophen-2-yl)-[2,2'-bithiophen]-5-yl] methylene)-6-oxo-5,6-dihydro-4*H*-cyclopenta[*b*]thiophen-4-ylidene)malonitrile

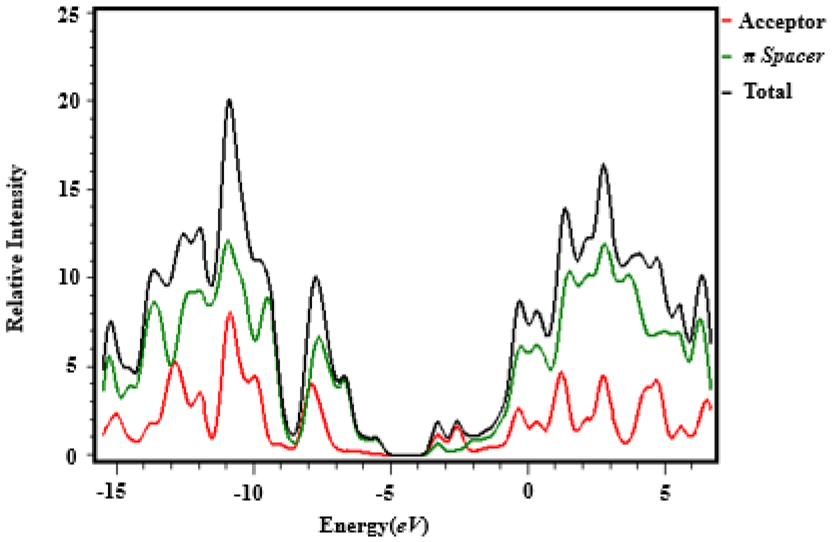

TNPR

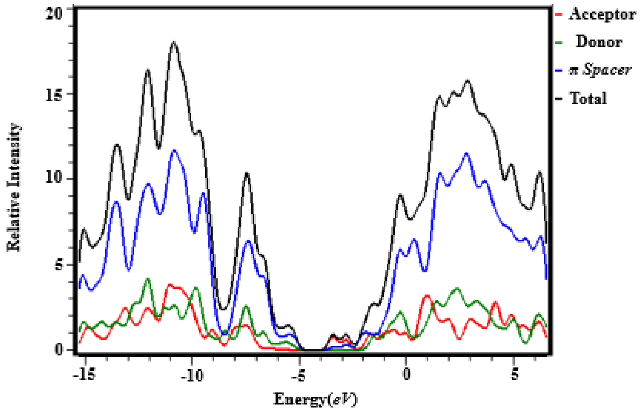

TNPD1

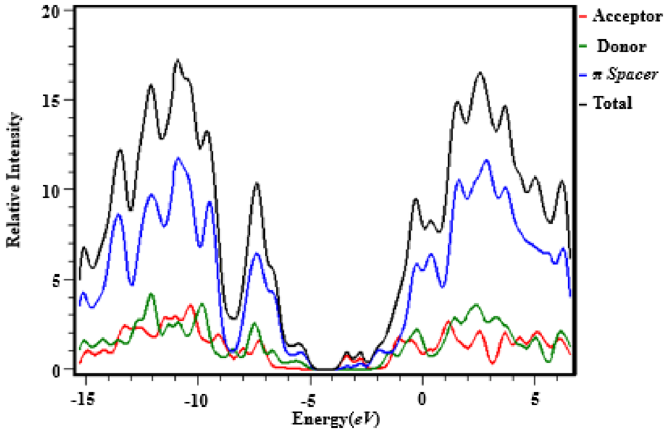

TNPD2

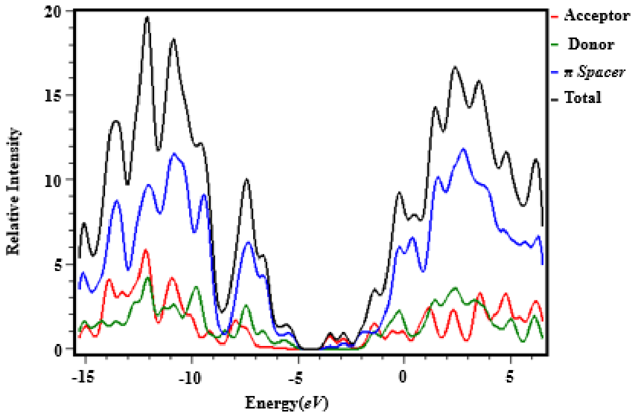

TNPD3

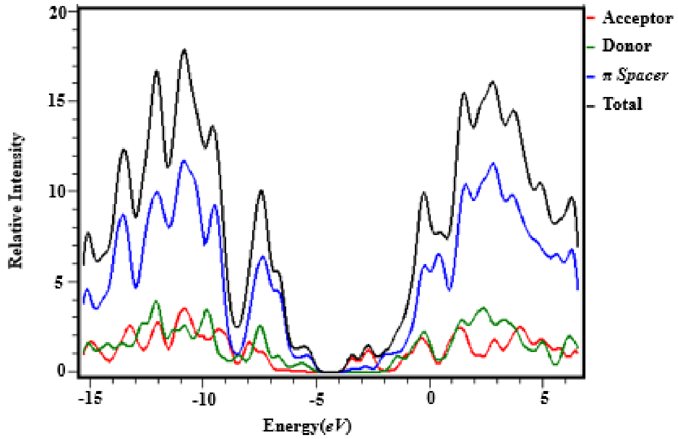

TNPD4

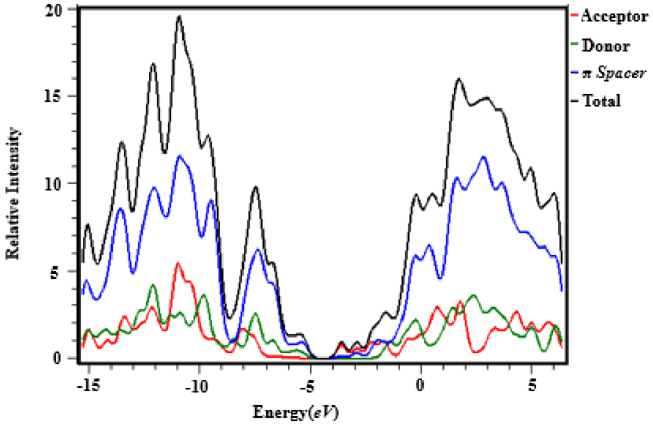

TNPD5

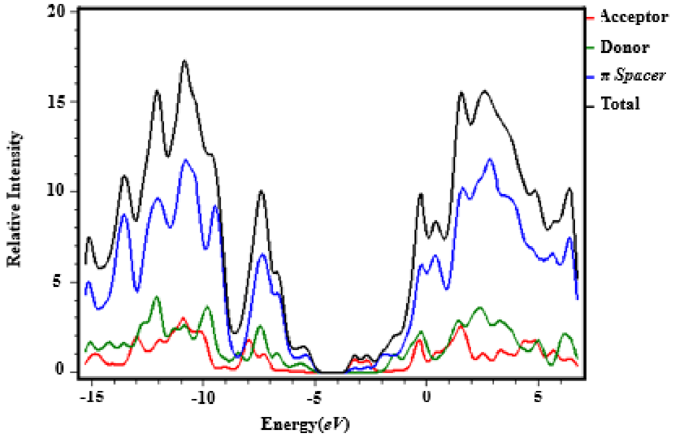

TNPD6

Figure S1: DOS plots of reference (**TNPR**) and designed compounds (**TNPD1** to **TNPD6**)

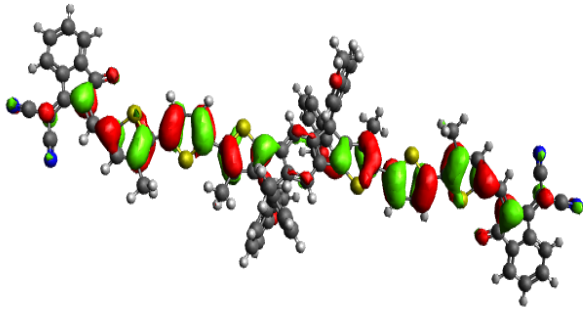

HOMO-1

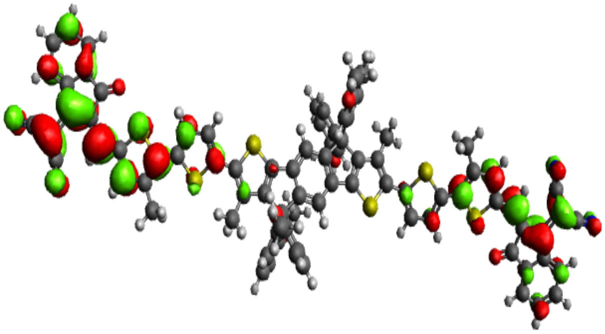

LUMO+1

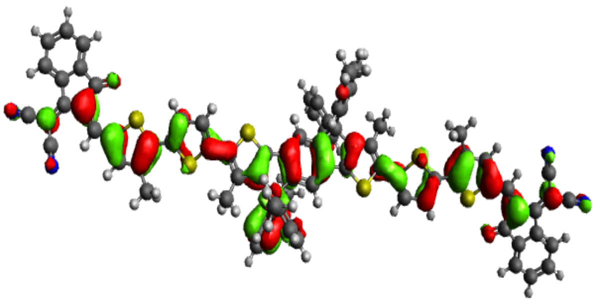

HOMO-2

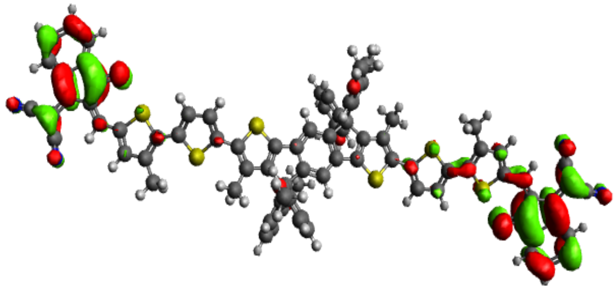

LUMO+2

TNPR

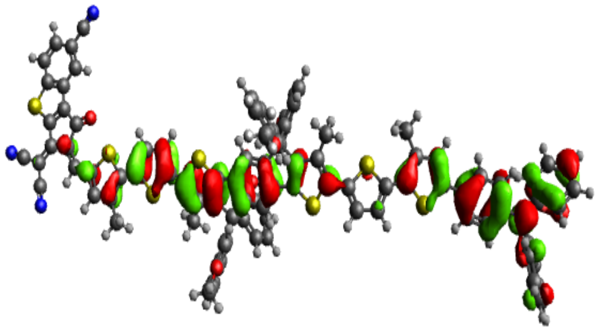

HOMO-1

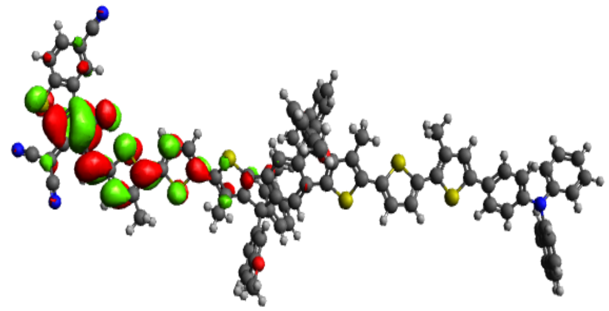

LUMO+1

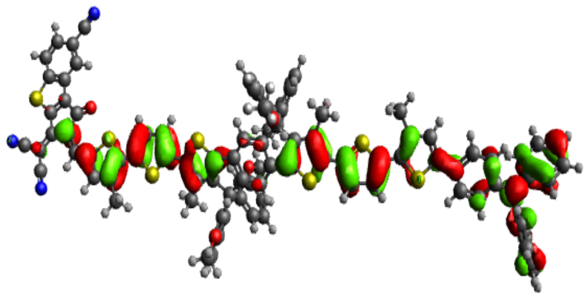

HOMO-2

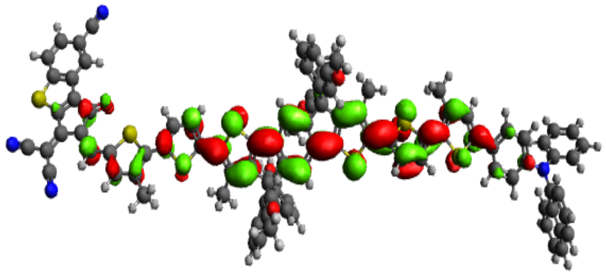

LUMO+2

TNPD1

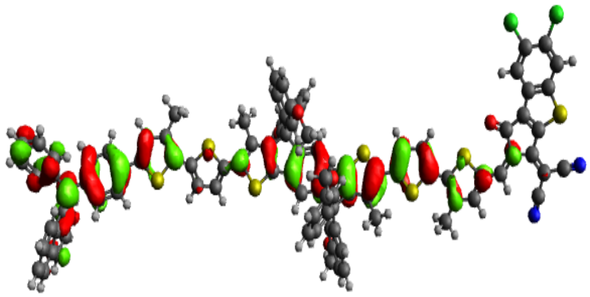

HOMO-1

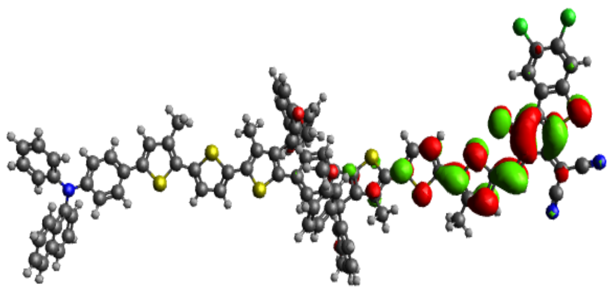

LUMO+1

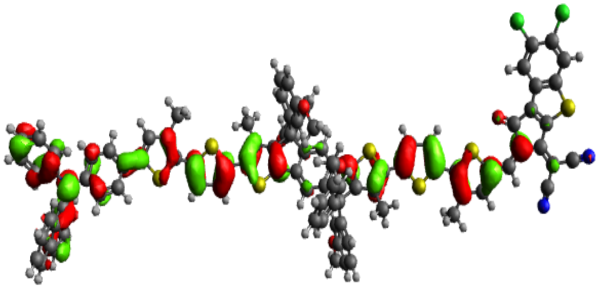

HOMO-2

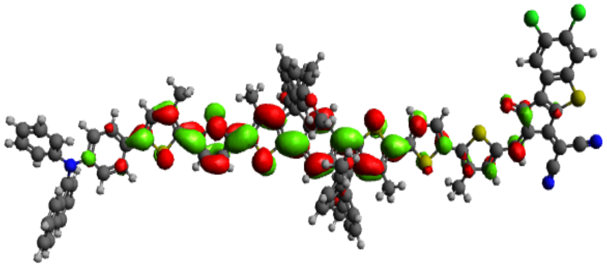

LUMO+2

TNPD2

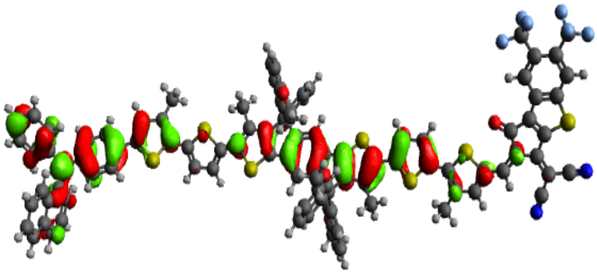

HOMO-1

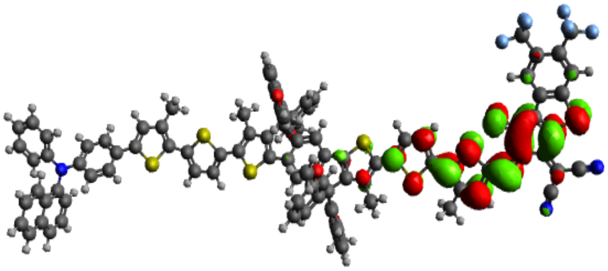

LUMO+1

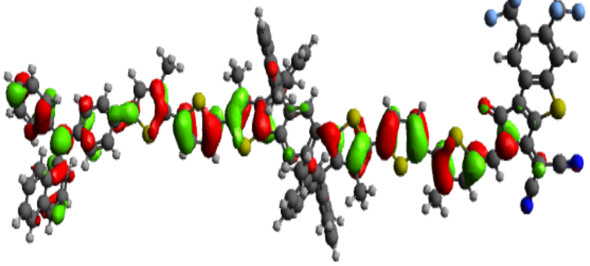

HOMO-2

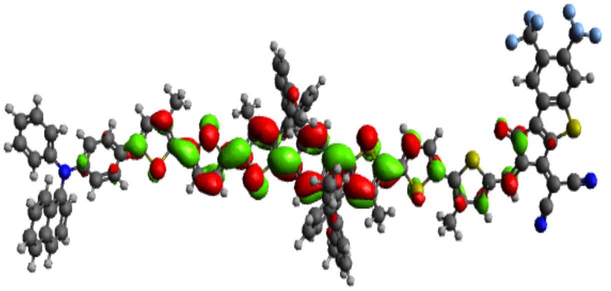

LUMO+2

TNPD3

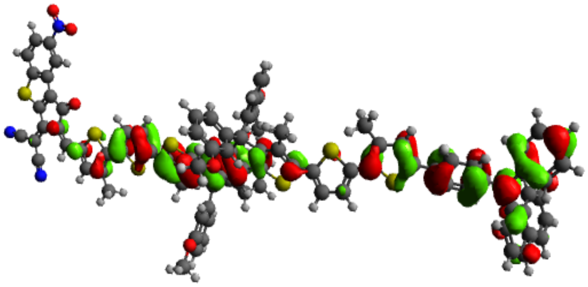

HOMO-1

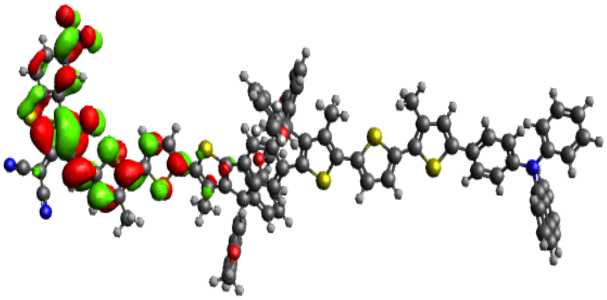

LUMO+1

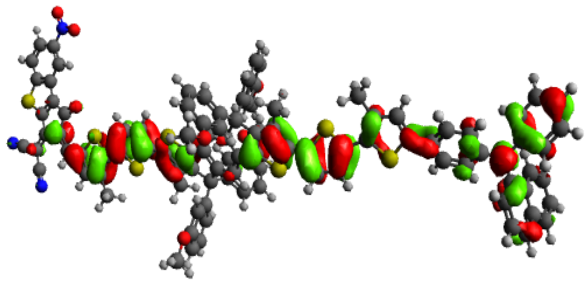

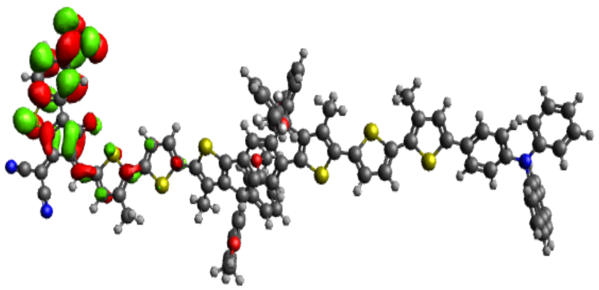

HOMO-2 **LUMO+2**

TNPD4

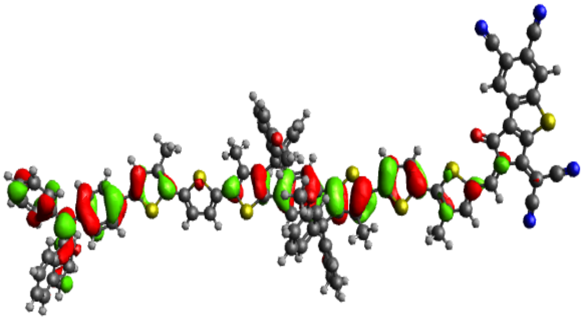

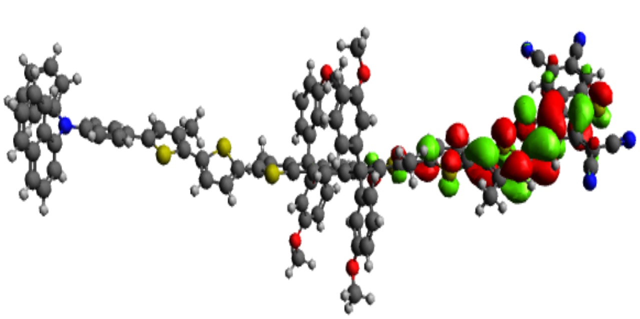

HOMO-1 **LUMO+1**

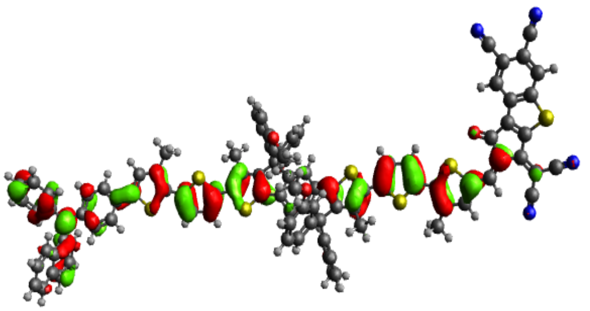

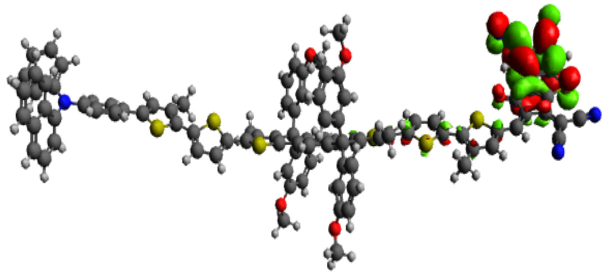

HOMO-2 **LUMO+2**

TNPD5

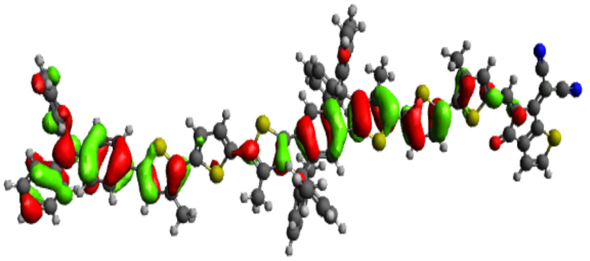

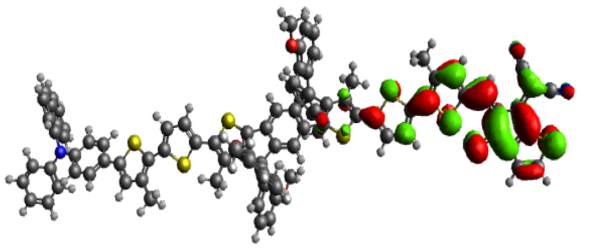

HOMO-1 **LUMO+1**

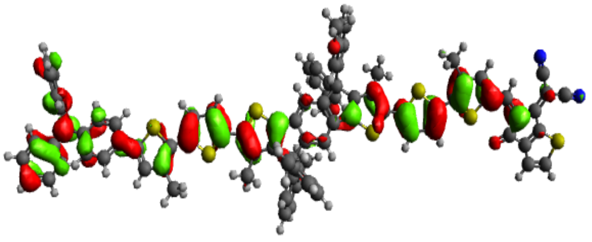

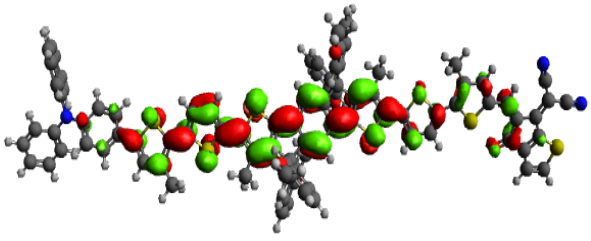

HOMO-2 LUMO+2

TNPD6

Figure S2: E_{HOMO-1}, E_{LUMO+1}, E_{HOMO-2} and E_{LUMO+2} contoured structures of reference (**TNPR**) and designed compounds (**TNPD1-TNPD6**)

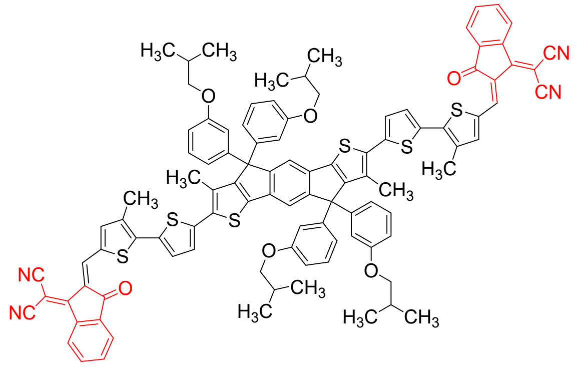

TNPR

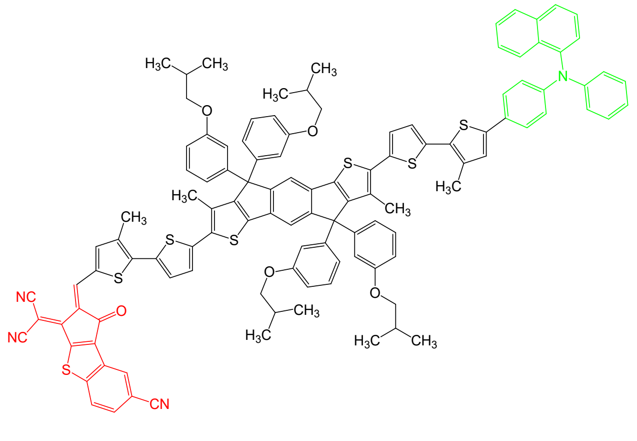

TNPD1

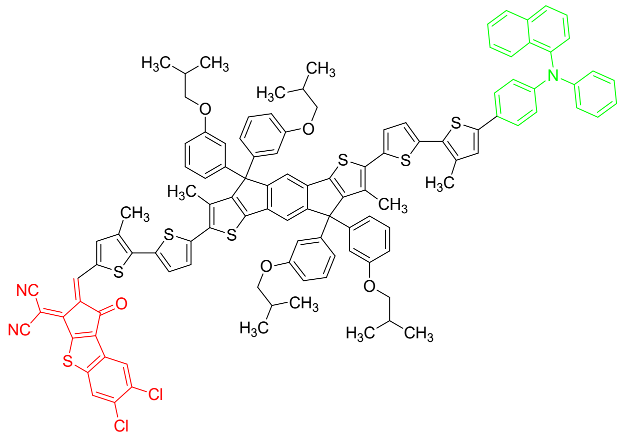

TNPD2

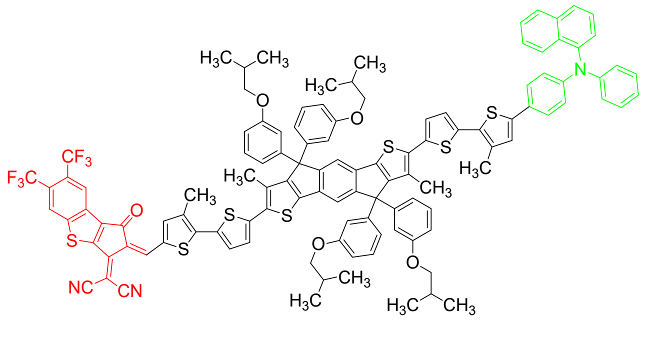

TNPD3

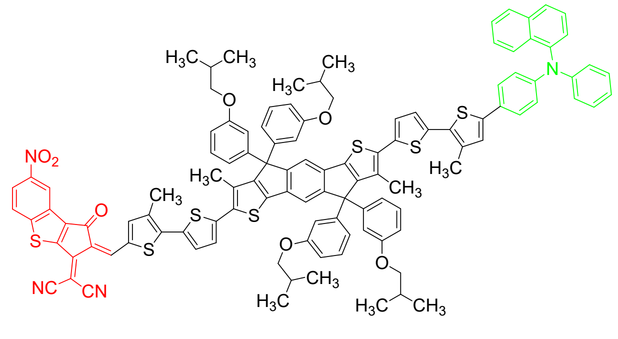

TNPD4

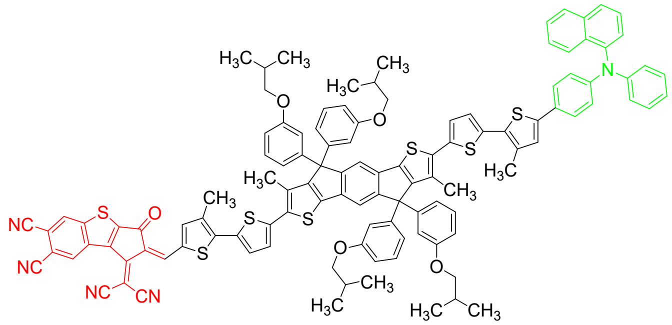

TNPD5

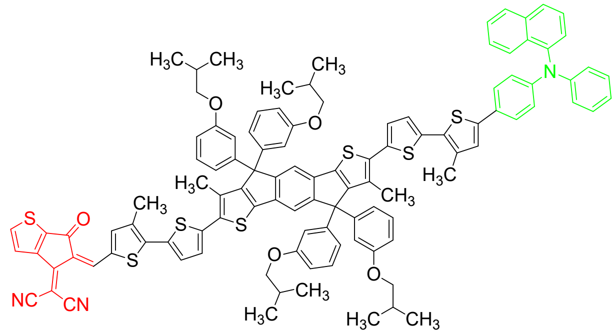

TNPD6

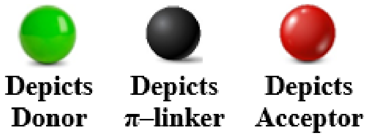

Figure S3: Structures of reference (**TNPR**) and designed compounds (**TNPD1-TNPD6**)

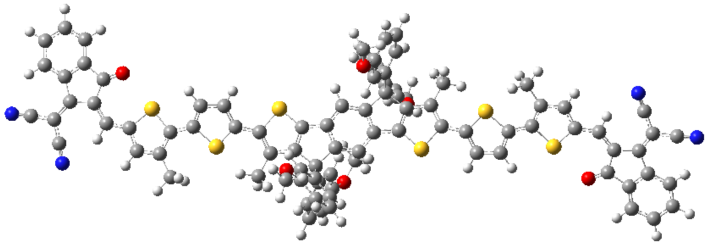

TNPR

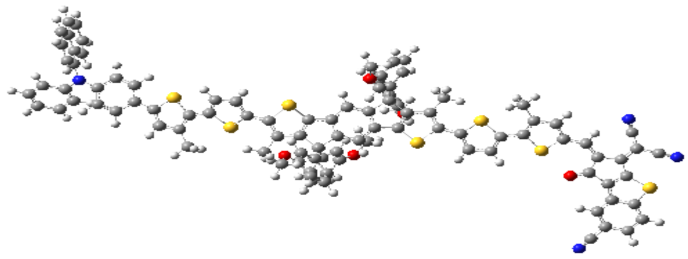

TNPD1

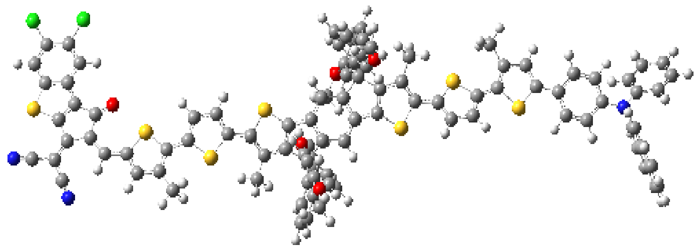

TNPD2

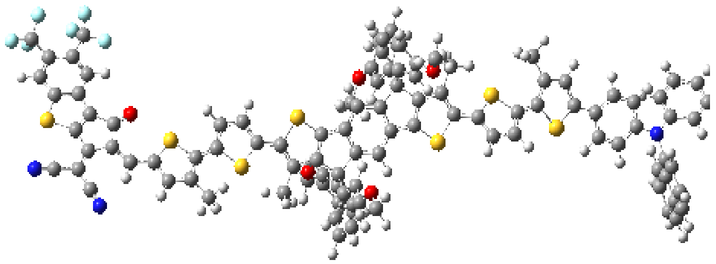

TNPD3

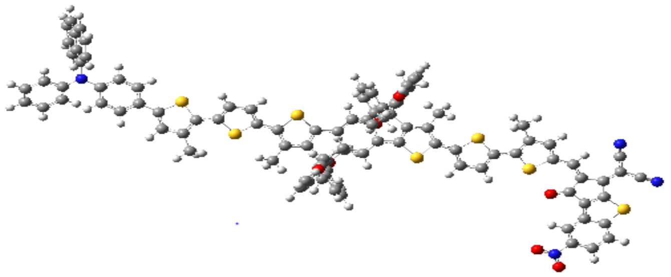

TNPD4

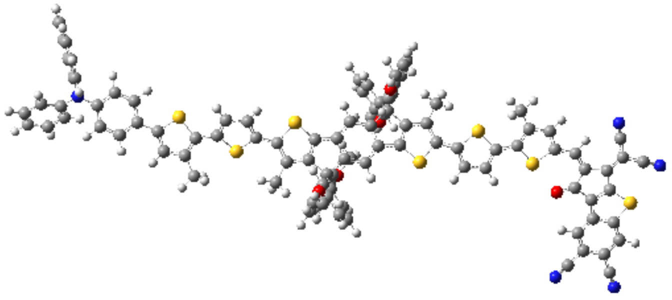

TNPD5

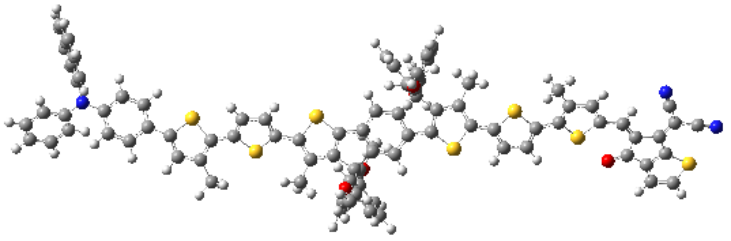

TNPD6

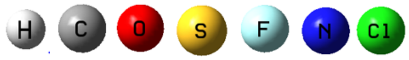

Figure S4: Optimized structures of investigated molecules (**TNPR** and **TNPD1-TNPD6**)
